# Supplementary material for: V-pipe 3.0: a sustainable pipeline for within-sample viral genetic diversity estimation
Source: Gigascience. 2024 Sep 30;13:giae065. doi: 10.1093/gigascience/giae065 (PMC11440432; doi:10.1093/gigascience/giae065)
Supplement: giae065_GIGA-D-23-00330_Revision_1 [file giae065_giga-d-23-00330_revision_1.pdf]

GigaScience

V-pipe 3.0: a sustainable pipeline for within-sample viral genetic diversity estimation

--Manuscript Draft--

**Manuscript Number:**	GIGA-D-23-00330R1
**Full Title:**	V-pipe 3.0: a sustainable pipeline for within-sample viral genetic diversity estimation
**Article Type:**	Research
**Funding Information:**	H2020 Marie Skłodowska-Curie Actions (955974) — Mrs Lara Fuhrmann
**Abstract:**	The large amount and diversity of viral genomic datasets generated by next-generation sequencing technologies poses a set of challenges for computational data analysis workflows, including rigorous quality control, scaling to large sample sizes, and tailored steps for specific applications.<br>Here, we present V-pipe 3.0, a computational pipeline designed for analyzing next-generation sequencing data of short viral genomes. It is developed to enable reproducible, scalable, adaptable, and transparent inference of genetic diversity of viral samples. By presenting two large-scale data analysis projects, we demonstrate the effectiveness of V-pipe 3.0 in supporting sustainable viral genomic data science.
**Corresponding Author:**	Niko Beerenwinkel<br>ETH Zurich D-BSSE: Eidgenossische Technische Hochschule Zurich Department of Biosystems Science and Engineering<br>Basel, SWITZERLAND
**Corresponding Author Secondary Information:**	
**Corresponding Author's Institution:**	ETH Zurich D-BSSE: Eidgenossische Technische Hochschule Zurich Department of Biosystems Science and Engineering
**Corresponding Author's Secondary Institution:**	
**First Author:**	Lara Fuhrmann
**First Author Secondary Information:**	
**Order of Authors:**	Lara Fuhrmann
	Kim Philipp Jablonski
	Ivan Topolsky
	Aashil A Batavia
	Nico Borgsmüller
	Pelin Icer Baykal
	Matteo Carrara
	Chaoran Chen
	Arthur Dondi
	Monica Dragan
	David Dreifuss
	Anika John
	Benjamin Langer
	Michal Okoniewski
	Louis Du Plessis
	Uwe Schmitt

Powered by Editorial Manager® and ProduXion Manager® from Aries Systems Corporation

	Franziska Singer
	Tanja Stadler
	Niko Beerenwinkel
**Order of Authors Secondary Information:**	
**Response to Reviewers:**	We are first addressing the editor's comments, and then the specific reviewer's comments. Our answers are marked with “A:”.<br><br># Editor's comments<br>– The 2 synthetic benchmark datasets generated should be made be made available.<br><br>A: The synthetic benchmark datasets can be reproduced using the Snakemake workflow available on GitHub [cite]. To ensure full reproducibility we have fixed the random seed for the generation of the synthetic benchmark datasets. We have added a step-by-step guide (https://github.com/cbg-ethz/V-pipe/tree/master/resources/auxiliary_workflows/benchmark/resources/multi_setup) to the GitHub repository that is explaining how to reproduce those datasets.<br><br>– The relevant command line scripts (or suitable methods document) used to run the comparative workflow/software tools including all parameters and versions should be made be made available.<br><br>A: The scripts to run the methods including all parameters and versions can be found on GitHub. We have added a README.md document (https://github.com/cbg-ethz/V-pipe/tree/master/resources/auxiliary_workflows/benchmark/resources/multi_setup) explaining the exact location of those scripts, including a step-by-step guide on how to reproduce the analysis including Figure 3 from the manuscript.<br><br>– For full transparency it would be very useful to provide a direct link to the 5-virus-mix sequence data mentioned here "We test the global haplotype reconstruction methods on sequencing reads from the 5-virus-mix presented in [15]." This would enable users to more easily locate and download the correct sequence data (I was unable to find it).<br><br>A: We have added the accession number of the 5-virus-mix sequencing data to the main text (line 504) and also to the ‘Availability of data and materials’ section (line 564).<br><br>– register V-pipe 3.0 in the bio.tools and SciCrunch.org databases to receive RRID (Research Resource Identification Initiative ID) and biotoolsID identifiers, and include these in your manuscript.<br><br>A: We have added the biotoolsID and the RRID to the manuscript in the section “Availability of data and materials”. While trying to update the entry on bio.tools the “Save” button gave us a “403 Request Blocked” error, we have contacted the developers but have not yet received an answer. We will update as soon as possible.<br><br>– Computational workflows should be registered in workflowhub.eu and the DOIs cited in the relevant places in the manuscript.<br><br>A: We have updated our entry on workflowhub.eu and have added the DOI in the section “Availability of data and materials”.<br><br><br># Reviewers comments<br><br>Reviewer #1:<br>The manuscript showcases a computational pipeline designed for analyzing next generation sequencing data of short viral genomes, namely V-pipe 3.0. After an overview of the challenge the tool is addressing, i.e. the necessity of continuous benchmarking of various methods due to their diverse performance across different scenarios,the paper continues with a detailed listing of the results, highlighting the key elements of Reproducibility, Scalability, Adaptability and Transparency. The next section provides some details on the three applications / demonstrations of V-Pipe 3.0, i.e the Swiss SARS-CoV-2 Sequencing Consortium, the Swiss surveillance of SARS-

Powered by Editorial Manager® and ProduXion Manager® from Aries Systems Corporation

	CoV-2 genomic variants in wastewater and the Global haplotype reconstruction benchmark. This is followed by a comprehensive comparison of V-Pipe 3.0 то other relevant viral bioinformatics pipelines for within sample diversity estimation, focusing on functionalities and sustainability, and specifically nf-core/viralrecon, HAPHPIPE and ViralFlow, as well as a section discussing the main advantages of V-Pipe 3.0 as well as the rationale for some of the identified drawbacks. The paper concludes with a thorough description of the underlying methods of V-Pipe 3.0 as well as on the data used.<br><br>Overall the paper gives a very good presentation of V-Pipe, and makes a strong case about its use and value in a real-world challenge. An overall comment is that there is some confusion on the role of V-Pipe 3.0 as a workflow - i.e. whether it's a dynamic system that uses different tools per step based on user input, or if it's an automated systems that benchmarks the analysis using (e.g.) synthetic data as the baseline.<br><br>A: Thank you for your comment. Indeed, the role of V-pipe 3.0 as a workflow and the new benchmarking module should be differentiated better. The benchmarking module is a new feature that was introduced in V-pipe 3.0. We have added the following sentence about V-pipe 3.0 to lines 113-116: “Depending on the user input, V-pipe 3.0 dynamically utilizes different tools for each processing step. In addition to the NGS data processing pipeline, we have incorporated a sub-workflow for benchmarking. This benchmarking module allows users to compare viral diversity estimation methods using synthetic and experimental data.”<br><br>In either case, there are also a few unclear points in the manuscript itself that could be further improved. Specifically:<br>-- It is not clear how V-pipe 3.0 differs from V-pipe. Although there is an indication of significant differences, an overview of the new features implemented in this version and/or a small introductory paragraph would be useful.<br><br>A: We have extended Table 1 to include V-pipe and highlight the new features. Additionally, we also include V-pipe in Table 2 and highlight the differences in terms of sustainability. Thank you for your suggestions, we believe they improve the clarity of the manuscript significantly.<br><br>-- In the "Results" section, lines 130 - 225 appear to refer to the implemented methodology and might be better served as part of the "Methods" section<br><br>A: While we acknowledge that this section of the Results may appear technical, we believe that its inclusion in the Results section is essential in this case as it reflects the outcomes of our development choice for the pipeline.<br><br>-- In the "Results" section, lines 135 - 138 implied that GitHub Actions are used to ensure Reproducibility of the workflow. Some more elaboration on this would be very useful, as GitHub actions are commonly used to automate processes (such as testing, conflict resolution etc). In particular, an reproducibility issue that might not be resolvable by GitHub actions are dependency conflicts that are specific to the particular system that is being tested.<br><br>A: Thank you for this comment. We have written workflow scripts that are executed via GitHub Actions to perform installation and execution tests. To improve clarity, we have added the following sentence in lines 142-147 including a direct reference to the definition of those workflow scripts:<br>“To ensure successful installation and reproducible execution on different systems, we have written GitHub Actions [19], workflow scripts [20] which automatically perform test installations of V-pipe 3.0 on Mac OS and Linux systems and run end-to-end tests by executing tutorials with real example data. For each update of V-pipe 3.0 these workflow scripts are automatically executed and report about installation problems or issues on the test data.”<br><br>-- In the "Results" section, lines 139 - 146, it's not clear how the benchmark study contributes to the overall reproducibility of V-pipe 3.0. Some further explanation of the rationale would be very useful here.

	A: Thank you for this comment. We agree that this paragraph can be improved. We have rewritten the paragraph (lines 148-158):<br>“Additionally, V-pipe 3.0 enables reproducible benchmarking of viral diversity estimation methods using the newly integrated benchmarking module, a critical capability considering the constant evolution of new methods and the availability of new datasets. The benchmarking module is a Snakemake based workflow which automatically applies a set of selected tools to various synthetic and real data sets, computes their respective performances in terms of precision and recall, and summarizes the results. The benchmarking workflow is itself sustainably implemented. Adding new tools and data sets to this benchmark is very easy and only requires the addition of a single file and no further modifications of the workflow. As a concrete demonstration of the effectiveness of the benchmarking module, we conducted a benchmarking study focused on global haplotype reconstruction (Section 3.3 below).”<br><br>-- In the "Results" section, lines 179 - 183, it is not clear how Git and GitHub ensure adaptability of any new features that are implemented. Usually a version control system/automation system, can facilitate the integration of new features, but it's not readily evident how it supports/ensures/facilitates adaptability. Maybe a definition of "adaptability" in this particular context could also help.<br><br>A: Thank you for this comment! We agree that the mentioning of version control at this point is rather confusing. We have removed lines 179-183 and added a compromised version in lines 194-198 to make the point that the integration of new features is tracked and tested to ensure smooth integration of new features. Below is the sentence we added to lines 190-194:<br>“Additionally, we run automated integration and unit tests using GitHub Actions workflows [19, 20] on every commit submitted to the repository. This verifies the reliability, consistency and correctness of the overall workflow. We use datasets from different viruses in our tests to make sure that V-pipe 3.0 and the newly added features are running successfully from start to end.”<br><br>-- In the "Applications" section, it is not clear which version of V-Pipe was used for the overall analysis (V-pipe or V-pipe 3.0), especially in the wastewater use case.<br><br>A: This comment is very valid. Indeed V-pipe 3.0 was developed in the course of the SARS-CoV-2 pandemic to address the new challenges not only in terms of scalability and the need of new features, but also the need to produce reproducible and transparent results. To clarify this we have added a sentence to the introduction lines 102-105:<br>“V-pipe 3.0 builds upon the foundation of V-pipe [8], but has undergone significant extensions and refinements to increase functionality and to adhere to sustainable data processing standards [16]. The development of V-pipe 3.0 was primarily driven to address the new demands and challenges that became evident in the SARS-CoV-2 pandemic.”<br>Additionally, we clarify in the section “Applications” that the whole cohort of samples was reanalysed upon each update of V-pipe. Hence, the results presented were generated with the newest release of V-pipe 3.0. We have added the following sentences to the manuscript for clarification (lines 228-233):<br>“The development of V-pipe 3.0 was primarily driven by two large-scale SARS-CoV-2 surveillance projects. Continuous updates and extensions of the pipeline have been motivated by the evolving demands of the pandemic. With each workflow update, the entire sample cohort underwent reanalysis using the latest pipeline version. This approach guaranteed consistent results that align with the most recent advancements in the pipeline.”<br><br>-- In the section "Comparison to other workflows" it is not very clear which tools are implemented within V-pipe 3.0, which differences there are with previous version (V-pipe) and how these differ to other pipelines. A table that is summarizing these details and highlighting the differences would be very useful here.<br><br>A: Thank you for your comment. We have included a column listing the tools from the previous V-pipe in Table 1, with new features added in V-pipe 3.0 highlighted in bold. Furthermore, the original V-pipe has been incorporated into the comparison table (Table 2) to emphasize distinctions in reproducibility, scalability, adaptability, and

Powered by Editorial Manager® and ProduXion Manager® from Aries Systems Corporation

transparency.

Moreover, there are a few minor points that would enhance the readers' understanding:
-- (minor) In the Section "2.1 Reproducibility", it's mentioned that all software dependencies are defined in Conda environments, making V-pipe 3.0 portable between different computing platforms. Is there a particular reason why V-Pipe itself isn't implemented as a conda package directly?

A: As V-pipe 3.0 is a Snakemake workflow it is rather unintuitive to package it as a Conda package. Snakemake itself manages the workflow's execution and dependencies, and it is primarily focused on defining and running workflows rather than serving as a distributable package. Hence, we found that packaging it into a Conda environment may introduce unnecessary complexity without significant advantages in our specific context.

-- (minor) More often that not, the pandemic is named as COVID19, in contrast to the virus that is named "SARS-CoV-2". It may be useful to amend/update the references to the "SARS-CoV-2 pandemic" accordingly.

A: We agree that the use of the “SARS-CoV-2 pandemic” is more appropriate.

Reviewer #2:
V-pipe 3.0 is introduced as an advanced computational pipeline tailored for the analysis of next-generation sequencing data from short viral genomes. Designed to meet the challenges posed by the vast and diverse datasets generated by these technologies, V-pipe 3.0 emphasizes reproducibility, scalability, adaptability, and transparency. It achieves this by adhering to Snakemake's best practices, allowing easy swapping of virus-specific configuration files, and providing thoroughly tested examples online. The utility of V-pipe 3.0 is showcased through its application in two extensive data analysis projects, proving its efficacy in sustainable viral genomic data science.
Central to V-pipe 3.0 is its capacity for estimating viral diversity from sequencing data. A versatile benchmarking module has been developed to continuously assess various diversity estimation methods, accommodating the rapid advancements within this field. The pipeline simplifies the inclusion of new tools and datasets, supporting both synthetic and real experimental data. However, challenges in global haplotype reconstruction highlight the need for scalable methods that can accurately reflect the complex population structures of viruses and manage the uncertainties in the results. Some additional clarification in the manuscript would be appreciated.

1) I'm curious about how the efficiency is attained.

A: To achieve scalability on extremely large cohorts, efficient resource usage (memory, cpu) per sample is necessary. To achieve this several measures were implemented, firstly, V-pipe 3.0 dynamically specifies cluster resources to adapt to the specific data requirements, second, V-pipe 3.0 enables the parallel execution of unrelated data analysis steps, third, V-pipe 3.0 validates user configuration files using JSON Schema during startup to identify potential runtime errors early and lastly, V-pipe 3.0 splits centralized tasks among multiple compute nodes and perform per-sample distributed computation of summary statistic (see Section Results - Scalability lines 163-171).

2) Is it possible to utilize V-pipe for analyzing other microorganisms?

A: Yes, this depends on the tools selected. While bacteria may require tools different from the currently implemented ones, V-pipe 3.0 has successfully processed sequencing data from various different viruses, including, for example, the DNA virus HSV2 (Lezcano, Oscar M., et al. Parallel evolution and enhanced virulence upon in vivo passage of an RNA virus in Drosophila melanogaster. Virus Evolution 9.2 (2023)).

3) The authors might consider directing readers to the following review article for reference: https://genomebiology.biomedcentral.com/articles/10.1186/s13059-021-02328-9

A: Thank you for recommending this review article. However, we feel that directing

Powered by Editorial Manager® and ProduXion Manager® from Aries Systems Corporation

readers to chromosome-scale haplotype reconstruction methods might lead to confusion, as this topic is not addressed by V-pipe 3.0.

4) Identifying specific genes or genome regions with high polymorphism across different populations would be fascinating. How does V-pipe handle analysis in these highly variable regions?

A: V-pipe 3.0 enables the analysis of highly variable regions by integrating tools that can handle such scenarios. For example, the alignment tool ngshmmalign is especially useful if long deletions and high heterogeneity are present (Posada-Céspedes, Susana, et al. V-pipe: a computational pipeline for assessing viral genetic diversity from high-throughput data. Bioinformatics 37.12 (2021): 1673-1680).
Ultilimety, the handling of V-pipe 3.0 depends on the users tools choice, hence we provide various tools to choose from in the workflow that have their advantages and disadvantages in different settings (see section 4).
We have added a sentence in line 412-414 to emphasize this: “For example, for alignment V-pipe 3.0 supports BWA MEM, Bowtie 2, ngshmmalgin and minimap2 which allows the processing of samples with very high diversity regions.”. Further, our variety of haplotype reconstruction tools as listed in Table 1 enable the analysis of very diverse datasets.

Reviewer #3:
Review of Article: "V-pipe 3.0: A Sustainable Workflow for Diversity Estimation from Viral NGS Samples"
General Assessment
The article presents V-pipe 3.0, a Snakemake-based bioinformatics workflow designed for analyzing intra-sample variability in viral Next-Generation Sequencing (NGS) data. While the authors emphasize sustainability and scalability, several critical scientific concerns undermine the credibility and utility of the presented work.

Scientific Concerns
1. Poor Availability of Hypotheses, Results and Interpretations/Conclusions
The article lacks clarity regarding the actual results obtained from the implementation of V-pipe 3.0. While the authors mention the application of the workflow in large-scale projects ("Applications" 3.1, 3.2 and 3.3), specific outcomes, such as the accuracy of genetic evolution or diversity estimations or the identification of viral variants, are poorly presented.
Readers require concrete questions, findings and statistical analyses to assess the efficacy and reliability of the workflow. Without transparent reporting of results, the scientific value of the article diminishes significantly.
2. Ill-defined Hypotheses and Scientific Questions
The article fails to clearly articulate the underlying hypotheses or scientific questions addressed by V-pipe 3.0. While it outlines the technical aspects and functionalities of the workflow, the overarching scientific goals remain obscure.
Without well-defined research questions, it becomes challenging for readers to contextualize the significance of the presented work within the broader field of virology or computational biology.

A: Thank you for your comment. In this manuscript, we have presented V-pipe 3.0, a robust data processing pipeline for the analysis of heterogeneous viral NGS samples. Our primary objective is to establish sustainable data processing practices (section 1), resulting in the development of a versatile pipeline that caters to a comprehensive spectrum of diversity estimation tasks of mixed samples. Sustainability is demonstrated in terms of reproducibility (section 2.1), scalability which relies on the efficacy of the workflow (section 2.2), adaptability (section 2.3), and transparency (section 2.4). We demonstrate its applicability and practicalness by providing three applications (section 3). Two large-scale projects are presented where V-pipe 3.0 proved to enable sustainable and reliable data processing (see section 3.1 and 3.2), and the global haplotype reconstruction study is exemplary of the newly integrated benchmarking module (see section 3.3). We illustrate the practical utility and reliability of our pipeline, as well as the transparency of the results. These applications provide valuable insights into the pipeline's capabilities in addressing biological and virological research inquiries, solidifying the claim of a reliable solution for viral diversity estimation. Hence the results of this manuscript are not the results of the large-scale projects but rather

Powered by Editorial Manager® and ProduXion Manager® from Aries Systems Corporation

the implementation and successful application to the large-scale projects.

3. Unverifiable Conclusions
The article concludes with a comparison of V-pipe 3.0 to other workflows, highlighting its purported advantages in terms of sustainability and functionality. However, the evaluation lacks empirical evidence or comparative analyses to substantiate these claims.
Readers are unable to independently verify the superiority of V-pipe 3.0 over alternative workflows, thereby undermining the credibility of the conclusions drawn by the authors.
There is a missing mark that is difficult to understand in this comparison, which casts a shadow over its relevance. Several Galaxy workflows were used, very early during the SARS-CoV-2 pandemic (first publication in August 2020 in PLoS Pathogens), to monitor the emergence of viral variants or assess intra-host variations. These workflows are still actively used and regularly updated (https://galaxyproject.org/projects/covid19/).

A: Thank you for your comments and suggestions. In crafting the comparison table (Table 2), we meticulously applied transparent criteria as listed in the table and as further elucidated with explanations and illustrative examples in the main text, lines 355-406. These criteria can be independently checked by anyone who is interested by reading the corresponding code repositories, documentation, and research articles of the workflows included in the comparison.
To the best of our knowledge the submitted Table 2 is not missing any marks and is complete.
Thank you for making us aware of the Galaxy workflows, indeed they belong in our comparison table. Our inclusion criteria for a workflow to be integrated into the comparison is that it needs to be open source, actively maintained, and provide within-sample diversity estimates for Illumina sequencing reads. Hence, we have included the GalaxyProject SARS-CoV-2 analysis effort workflows (https://galaxyproject.org/projects/covid19/workflows/). Since they are usable together we are treating them together as one workflow. We have added also the original V-pipe pipeline to the comparison for clarification of the differences to V-pipe 3.0 as suggested by Reviewer #1. Hence, two new columns were added to Table 2: “V-pipe” and “GalaxyProject SARS-CoV-2 analysis effort”.
Additionally, we have adapted the corresponding Section 4, lines 356-414, in the manuscript to highlight the properties of the GalaxyProject workflows. We believe that these additions are substantially improving the value of the comparison. Thank you for your suggestions.

Technical Concerns
A limited number of target users
V-pipe can only be installed by researchers familiar with the use of a Bash script in a Linux terminal and the concepts of environments and dependencies. Once installed, the workflow must be configured to adapt it to specific needs, recognizing that most virologists have very different goals from national-level monitoring of intra- or inter-host variations. However, this configuration involves editing a yaml script and clearly requires relatively advanced programming skills given that V-pipe is complex software. Finally, even if a configuration file was built by a computer scientist, the correct execution of V-pipe, the access to the results as well as the verification that these results are computationally consistent can only be done by a person who is familiar with a Linux console and command lines to interact with. In the end, unfortunately, I do not believe that V-pipe will be useful to a large audience among virologists.

A: Indeed, V-pipe 3.0 requires computer literacy and basic command line knowledge. Virologists without any bioinformatics knowledge are not our target audience. However, in depth knowledge about environments and dependencies is not needed as V-pipe 3.0 is automatically generating, managing and activating the necessary environments. This is the advantage of workflow systems like Snakemeke which is the system V-pipe 3.0 is built on.
Also the yaml-file configuration does not require any advanced programming knowledge, but solely requires users to essentially be able to use a text editor. Subsequently, freely available online yaml validation tools can be used that can help users validate their modified yaml files.

Powered by Editorial Manager® and ProduXion Manager® from Aries Systems Corporation

In order to access the results no linux console or command line knowledge is needed as results and also verification of the successful execution can be accessed through simply clicking through the result directories and opening the respective error text files. We do not require users to check for “computational consistency”. We test parts of the computational consistency automatically as described in Section “Reproducibility” which is tested upstream before release (see lines 142-147).

As it stands, V-pipe workflows are difficult to reproduce.
Given the objectives stated in this work, I obviously expected to be able to reproduce the analyses presented in section 3 (Applications), or to be able to run - with V-pipe - a pilot analysis used for comparison with other workflows. Unless I am mistaken, I have found, neither in the manuscript nor in the GitHub repository, any configuration file and the associated input data to reproduce, at least partially, even one figure of the article.

A: Thank you for making us aware that the benchmark study is not easy to find on the GitHub repository. We are sorry to hear that you were unable to find the Snakemake workflow reproducing the benchmark study. In the original submission it was linked in the section “Availability of data and materials.” Now, we added the reference also to the section “Methods - Global haplotype reconstruction benchmark study“. In this GitHub repository directory, readers can find all configuration scripts needed to reproduce the analysis. This includes configuration files defining the simulation of the simulated data and scripts for the execution of the methods. Further the notebooks to generate the figures from Figure 3 in the manuscript are also available in this repository under “workflow/notebooks”, following the Snakemake workflow directory standards.
Additionally, we have added a step-by-step guide to reproduce the figures from Figure 3 in the manuscript which is also available in the GitHub repository directory (V-pipe/resources/auxiliary_workflows/benchmark/resources/multi_setup) under README.md.

If we focus only on the V-pipe GitHub repository, we find the necessary documentation to install it easily (with the caveats mentioned above) but there is no obvious example to test the use of V-pipe.

A: Thank you for your comment. We have two tutorials available on our GitHub repository that enable users to test the use of V-pipe 3.0 on two experimental datasets. We have updated the GitHub README such that the tutorials are now linked more prominently. Additionally, we have added a direct link to the two tutorials to the section “Results - Transparency” in line 216.

I'm lucky enough to be very familiar with GitHub Actions and so I looked for a testing workflow in these Actions that could serve as an example for a local test of V-pipe. I found the script "V-pipe/tests/regression_tests.sh", but its contents did not reassure me at all about the transparency of V-pipe workflows.

A: Thank you for this comment. As also reviewer #1 mentioned, the use of GitHub Actions for our testing needs to be described better. We have written workflow scripts that are executed via GitHub Actions to perform installation and execution tests. To improve clarity, we have added the following sentence in lines 142-147 including a direct reference to the definition of those workflow scripts (they are not in "V-pipe/tests/regression_tests.sh"):
“To ensure successful installation and reproducible execution on different systems, we have written GitHub Actions [19], workflow scripts [20] which automatically perform test installations of V-pipe 3.0 on Mac OS and Linux systems and run end-to-end tests by executing tutorials with real example data. For each update of V-pipe 3.0 these workflow scripts are automatically executed and report about installation problems or issues on the test data.”
Additionally, we added a README.md file to the GitHub directory "https://github.com/cbg-ethz/V-pipe/tree/master/tests” that points interested readers to the correct directory of all the workflow testing scripts. Those are in “https://github.com/cbg-ethz/V-pipe/tree/master/.github/workflows”. We have also added a README.md file for clarity to this directory: “https://github.com/cbg-ethz/V-pipe/blob/master/.github/workflows/README.md”

Powered by Editorial Manager® and ProduXion Manager® from Aries Systems Corporation

Clearly missing from the GitHub repository is a concrete example of using V-pipe with real input data and a step-by-step explanation of configuring Snakemake and running a workflow.

A: Thank you for pointing out that our tutorials with step-by-step explanations are not easily visible on our GitHub repository. We have two tutorials available on our GitHub repository that enable users to test the use of V-pipe 3.0 on two experimental datasets. These tutorials are step-by-step guides explaining the installation process, organization of the data, preparing the data, preparing the configuration, running V-pipe and analyzing the output.
We have updated the GitHub README such that the tutorials are now linked more prominently. Additionally, we have added a direct link to the two tutorials to the section "Results - Transparency" in line 216.

Recommendations
1. Enhanced Results Reporting
- The authors should provide detailed summaries of empirical findings obtained through the application of V-pipe 3.0. This includes accuracy metrics, performance benchmarks, and comparative analyses with existing methods.
- Statistical measures, such as precision, recall, and computational efficiency, should be clearly presented to enable rigorous evaluation by the scientific community.

A: In this manuscript, we have presented a workflow that enables sustainable data analysis, as detailed in the "Results" section. As V-pipe 3.0 offers various options of tools for each data processing step, the performance (like precision, recall, accuracy, etc) is given by the chosen tool. It is known that different tools excel in distinct scenarios, each with its set of advantages and limitations. Hence, it is the tools that need to prove their performance with statistical metrics such as precision and recall. The primary objective of a pipeline like V-pipe 3.0 is to ensure sustainable data processing. We have demonstrated sustainability of the pipeline by following the four aspects in Mölder's hierarchy: reproducibility (section 2.1), scalability (section 2.2), adaptability (section 2.3) and transparency (section 2.4). For each of the four aspects, we demonstrate the various developmental choices made to guarantee and achieve sustainability of V-pipe 3.0. Additionally, we prove the practicality of V-pipe 3.0 in real life scenarios by presenting three applications (section 3).

2. Clarification of Research Objectives
- Prioritize the elucidation of specific research questions or hypotheses addressed by V-pipe 3.0. Clearly articulate the scientific motivations behind the development and implementation of the workflow.
- By defining clear objectives, the authors can enhance the relevance and impact of their work within the field of viral genomics and bioinformatics.

A: Thank you for your comment. The primary motivation of the development of V-pipe 3.0 were the new challenges and demands that became evident with the onset of the SARS-CoV-2 pandemic. On the one hand this was the need of new features, like primer trimming or the frameshifts and stop codon diagnostics (Table 1). On the other hand, the large amount of newly available data and also the impact of analysis results, as we reported to public health decision makers, required sustainable data analysis. We have clarified this in the introduction in lines 102-106:
"V-pipe 3.0 builds upon the foundation of V-pipe [8], but has undergone significant extensions and refinements to increase functionality and to adhere to sustainable data processing standards [16]. The development of V-pipe 3.0 was primarily driven to address the new demands and challenges that became evident in the SARS-CoV-2 pandemic."
Additionally, we emphasize this objective by adding the following sentences to the Applications section in lines 228-233 for further clarification:
"The development of V-pipe 3.0 was primarily driven by two large-scale SARS-CoV-2 surveillance projects. Continuous updates and extensions of the pipeline have been motivated by the evolving demands of the pandemic. With each workflow update, the entire sample cohort underwent reanalysis using the latest pipeline version. This approach guaranteed consistent results that align with the most recent advancements in the pipeline."

Powered by Editorial Manager® and ProduXion Manager® from Aries Systems Corporation

	3. Empirical Validation of Conclusions<br>- Substantiate claims regarding the superiority of V-pipe 3.0 through rigorous empirical validation. Conduct thorough comparative analyses with alternative workflows, incorporating diverse datasets and performance metrics.<br>- Transparently report the results of benchmarking studies and provide access to raw data and code repositories to facilitate reproducibility and independent verification.<br><br>A: Thank you for this comment. With V-pipe 3.0 providing multiple tool options for each data processing step, the choice of tool determines performance metrics such as precision and recall. Also other workflows like HAPHPIPE (see section 4) provide various methods and tools per processing step. Hence a computational comparative analysis would not be meaningful as performance depends on the user-selected tools. In order to still provide an informative comparison, we have included the comparison table Table 2. This table allows readers to compare workflows with comparable purpose, to get an overview over the implemented features and also the sustainability of those workflows. Following your suggestion, we have also extended this table to include another relevant workflow that is hosted by Galaxy.<br>Additionally, to address your suggestion regarding the transparency of the benchmark study results, we have added a step-by-step guide on how to reproduce the benchmarking study to GitHub repository:<br>https://github.com/cbg-ethz/V-pipe/tree/master/resources/auxiliary_workflows/benchmark/resources/multi_setup<br><br>Conclusion<br>While the article introduces an ambitious bioinformatics workflow for analyzing viral NGS data, several scientific deficiencies undermine its credibility and utility. By addressing concerns related to results reporting, hypothesis formulation, and conclusion verification, the authors can enhance the scientific rigor and impact of their work.<br>In its current state, implementation of V-pipe in research projects is lacking detailed instructions if a large audience of biologists is targeted. Ultimately the question is not so much whether V-pipe workflows are reproducible, scalable, adaptable and transparent, but rather for whom. In my opinion, additional work on the general architecture of the software and its documentation can still significantly improve these 4 aspects in order to reach a wider audience of virologists.<br><br>A: We would like to thank the reviewer for their useful comments. We followed your suggestions on extending Table 2 and included the GalaxyProject SARS-CoV-2 analysis effort workflows (https://galaxyproject.org/projects/covid19/workflows/) into our comparison. Further, for transparency of the benchmark study results, we have added a step-by-step guide to the GitHub as a README in the directory https://github.com/cbg-ethz/V-pipe/tree/master/resources/auxiliary_workflows/benchmark/resources/multi_setup. This gives clear instructions on how to reproduce the analysis and the figures. All configuration files to produce the simulated data and all scripts executing the methods are available in this directory. Additionally, instead of only referring to the GitHub repository in the section “Availability of data and materials”, we have also added a direct link to the section “Methods”. Further to improve user experience we have updated the README on the V-pipe repository (https://github.com/cbg-ethz/V-pipe) with a more prominent link to the tutorials with two experimental datasets. Additionally, we have added a direct link to the two tutorials to the section “Results - Transparency” in line 216.<br>Moreover, we have improved the explanations on our testing structure using GitHub Actions. We have written workflow scripts that are executed via GitHub Actions to perform installation and execution tests, this is elaborated in lines 142-147 including a direct link to the definition of those workflow scripts (https://github.com/cbg-ethz/V-pipe/tree/master/.github/workflows). To guide interested readers better, we have also added READMEs to the GitHub directory explaining which tests are run.
**Additional Information:**	
**Question**	**Response**
Are you submitting this manuscript to a special series or article collection?	No

Powered by Editorial Manager® and ProduXion Manager® from Aries Systems Corporation

**Experimental design and statistics**<br><br>Full details of the experimental design and statistical methods used should be given in the Methods section, as detailed in our Minimum Standards Reporting Checklist. Information essential to interpreting the data presented should be made available in the figure legends.<br><br>Have you included all the information requested in your manuscript?	Yes
**Resources**<br><br>A description of all resources used, including antibodies, cell lines, animals and software tools, with enough information to allow them to be uniquely identified, should be included in the Methods section. Authors are strongly encouraged to cite Research Resource Identifiers (RRIDs) for antibodies, model organisms and tools, where possible.<br><br>Have you included the information requested as detailed in our Minimum Standards Reporting Checklist?	Yes
**Availability of data and materials**<br><br>All datasets and code on which the conclusions of the paper rely must be either included in your submission or deposited in publicly available repositories (where available and ethically appropriate), referencing such data using a unique identifier in the references and in the "Availability of Data and Materials" section of your manuscript.<br><br>Have you have met the above requirement as detailed in our Minimum Standards Reporting Checklist?	Yes

Click here to access/download;LaTeX - Main Document (TeX file);main.tex

V-pipe 3.0: a sustainable pipeline for within-sample viral genetic diversity estimation

Lara Fuhrmann [1,2†], Kim Philipp Jablonski [1,2†], Ivan Topolsky [1,2†], Aashil A Batavia [1,2], Nico Borgsmüller [1,2], Pelin Icer Baykal [1,2], Matteo Carrara [2,4], Chaoran Chen [1,2], Arthur Dondi [1,2], Monica Dragan [1,2], David Dreifuss [1,2], Anika John [1,2], Benjamin Langer[1], Michal Okoniewski [3], Louis du Plessis [1,2], Uwe Schmitt [3], Franziska Singer [4], Tanja Stadler [1,2], Niko Beerenwinkel [1,2*]

[1]Department of Biosystems Science and Engineering, ETH Zurich, Basel, 4056, Switzerland.
[2]SIB Swiss Institute of Bioinformatics, Lausanne, 1015, Switzerland.
[3]Scientific IT Services, ETH Zurich, Zurich, 8092, Swizterland.
[4]NEXUS Personalized Health Technologies, ETH Zurich, Basel, 4058, Swizterland.

*Corresponding author(s). E-mail(s): niko.beerenwinkel@bsse.ethz.ch;
Contributing authors: lara.fuhrmann@bsse.ethz.ch; kim.jablonski@bsse.ethz.ch; ivan.topolsky@bsse.ethz.ch; aashilbatavia@gmail.com; nico.borgsmueller@bsse.ethz.ch; pelin.icer@bsse.ethz.ch; carrara@nexus.ethz.ch; chaoran.chen@bsse.ethz.ch; arthur.dondi@bsse.ethz.ch; monica.dragan@bsse.ethz.ch; david.dreifuss@bsse.ethz.ch; anika.john@bsse.ethz.ch; blanger@student.ethz.ch; michal.okoniewski@id.ethz.ch; louis.duplessis@bsse.ethz.ch; uwe.schmitt@id.ethz.ch; singer@nexus.ethz.ch; tanja.stadler@bsse.ethz.ch;
[†]These authors contributed equally to this work.

Abstract

The large amount and diversity of viral genomic datasets generated by next-generation sequencing technologies poses a set of challenges for computational data analysis workflows, including rigorous quality control, scaling to large sample sizes, and tailored steps for specific applications. Here, we present V-pipe 3.0, a computational pipeline designed for analyzing next-generation sequencing data of short viral genomes. It is developed to enable reproducible, scalable, adaptable, and transparent inference of genetic diversity of viral samples. By presenting two large-scale data analysis projects, we demonstrate the effectiveness of V-pipe 3.0 in supporting sustainable viral genomic data science.

Keywords: next-generation sequencing, NGS data processing, sustainable data analysis workflow, benchmark, global haplotype reconstruction, viral genetic diversity

1 Background

With the advent of next-generation sequencing (NGS) technologies, large amounts of viral genomic data are being generated, which can no longer be easily analyzed on personal computers [1]. As this availability of high-coverage data sets brings interesting research opportunities but also computational challenges, many new processing and analysis tools are being developed. In particular, new possibilities of characterizing viral variants and analyzing the genetic diversity of viral sequencing samples have emerged [2, 3]. While inter-host variability describes how viral strains differ between separate hosts, intra-host, or within-host, variability measures the diversity of viral strains within a single host. Within-host genetic diversity is especially relevant to understanding disease progression and treatment options [4, 5]. In addition to clinical or experimental samples, there has been an increasing abundance of environmental samples also showing high within-sample variability, such as wastewater samples. These samples can contain a diverse array of viruses, enabling the monitoring of pathogens on a larger scale, encompassing cities, regions, and countries [6, 7].

For estimation of within-sample diversity from NGS samples, several data processing steps and tools are needed. Due to the complexity of the data, these tools should

be executed as part of a processing workflow. Typically they comprise tools for quality control, sequence alignment, consensus sequence assembly, diversity estimation, and result visualization. Various workflows have been proposed which try to accomplish these goals including V-pipe [8], ViralFlow [9], nf-core/viralrecon [10] and HAPHPIPE [11]. The adaptability of these workflows becomes crucial as different types of viruses require tailored analysis approaches. This need became evident during the SARS-CoV-2 pandemic, emphasizing the rapid emergence of specific requirements vital to public health [12]. For example, samples originating from diverse sources, such as clinical or wastewater settings, require application-specific processing steps that need to be supported in the same workflow.

Another effect of the SARS-CoV-2 pandemic is that a substantial increase in sequencing capacities has led to unprecedentedly large numbers of samples becoming publicly available, e.g., on the European Nucleotide Archive (ENA; [13]) or GenBank [14]. Analysis workflows need to be able to handle such large amounts of data in order to be beneficial to public health and epidemiological advances. Hence, it is critical for workflows to not only include a broad range of functionalities, but also to enable and promote sustainable data processing practices to ensure their effectiveness and long-term success.

NGS data processing workflows offer a range of diversity estimation approaches at different spatial genomic scales: mutation calling, local and global haplotype. Mutation calling refers to detecting single base pair mutations or variations at specific positions within the genome. Global haplotypes refer to the reconstruction of complete haplotypes that span the entire length of the viral genome. Midway between single mutations and global haplotypes, local haplotypes focus on identifying genomic variants spanning short genomic regions that are entirely covered by sequencing reads. The reconstruction of global haplotypes is the most challenging task as multiple reads

need to be assembled together to cover a whole genome, but it provides the most comprehensive measure of viral diversity [15]. Local haplotypes not only estimate local viral diversity directly, but they also provide the mutation calls of highest accuracy by leveraging locally co-occurring mutations.

As the methodologies for viral diversity estimation and data sources can be heterogeneous, understanding the performance of each tool and benchmarking them in a realistic way is difficult. Additionally, different methods may excel in different scenarios. Therefore, continuous benchmarking of these methods is crucial to identify the most suitable one for any given data source and scenario. Consequently, it is important to provide data analysis procedures as publicly available workflows designed in a sustainable manner. This approach facilitates continuous re-evaluation of the benchmarking workflow with new and updated parameter settings. This is needed as new methods are being developed which have to be compared to already existing ones and new test data sets become available, either new synthetic data sets with new simulation setups, or real data sets with new experimental setups. Finally, completely new application domains can appear which require adapting the existing benchmarking workflow.

Here, we present V-pipe 3.0, a sustainable data analysis workflow for diversity estimation from viral NGS samples. Sustainability comprises reproducibility, scalability, adaptability, and transparency of the workflow [16]. V-pipe 3.0 builds upon the foundation of V-pipe [8], but has undergone significant extensions and refinements to increase functionality and to adhere to sustainable data processing standards [16]. The development of V-pipe 3.0 was primarily driven to address the new demands and challenges that became evident in the SARS-CoV-2 pandemic. We highlight how the workflow has been designed to achieve these properties and describe how they have been crucial for the application of V-pipe 3.0 to large-scale data analysis projects. In

particular, we present a new and efficient workflow that enables the processing of hundreds of thousands of samples. We demonstrate how automated source code testing makes it possible to quickly make new functionalities and bug fixes available to end users and how its modular design allows to quickly implement application-specific features. Depending on the user input, V-pipe 3.0 dynamically utilizes different tools for each processing step. In addition to the NGS data processing pipeline, we have incorporated a sub-workflow for benchmarking. This benchmarking module allows users to compare viral diversity estimation methods using synthetic and experimental data. The module itself is sustainably implemented and it enables adding new methods and test data sets. We demonstrate its use by conducting a benchmarking study where we apply a set of global haplotype reconstruction methods to both synthetic and real data sets. Lastly, we compare V-pipe 3.0 to workflows for similar applications, provide an overview of their functionalities, and compare their features in terms of sustainability.

2 Results

V-pipe 3.0 is a bioinformatics workflow which combines various tools for analyzing viral NGS data (Table 1). It is based on V-pipe, a pipeline designed for analyzing NGS data of short viral genomes [8] and extends it not only in terms of functionalities (Table 1) but also by consistently implementing principles of sustainable data analysis (Table 2). In the initial step of the pipeline, the raw sequencing reads in fastq format undergo a quality control process. Following this, the reads are aligned, and subsequently, the user-specified diversity estimation methods are executed (Figure 1). To ensure sustainable data analysis, we followed the hierarchy of sustainability proposed in [16] and created a reproducible, scalable, adaptable, and transparent workflow. It has been widely recognized that these aspects are crucial to scientific progress but often lacking in current literature [17, 18]. In the following, we will provide a detailed explanation of the reimplementation and extensions that were undertaken during the development

of V-pipe 3.0. To demonstrate that V-pipe 3.0 effectively addresses the challenges of sustainable data analysis we follow the four aspects in Mölder et al.'s hierarchy [16].

2.1 Reproducibility

Reproducibility allows other researchers to execute an existing workflow and obtain the exact same results as the original workflow authors. To achieve this goal, we define all software dependencies in Conda environments, which makes V-pipe 3.0 portable between different computing platforms. That way, V-pipe 3.0 can be executed without complicated, manual installation procedures. To ensure successful installation and reproducible execution on different systems, we have written GitHub Actions [19], workflow scripts [20] which automatically perform test installations of V-pipe 3.0 on Mac OS and Linux systems and run end-to-end tests by executing tutorials with real example data. For each update of V-pipe 3.0 these workflow scripts are automatically executed and report about installation problems or issues on the test data.

Additionally, V-pipe 3.0 enables reproducible benchmarking of viral diversity estimation methods using the newly integrated benchmarking module, a critical capability considering the constant evolution of new methods and the availability of new datasets. The benchmarking module is a Snakemake based workflow which automatically applies a set of selected tools to various synthetic and real data sets, computes their respective performances in terms of precision and recall, and summarizes the results. The benchmarking workflow is itself sustainably implemented. Adding new tools and data sets to this benchmark is very easy and only requires the addition of a single file and no further modifications of the workflow. As a concrete demonstration of the effectiveness of the benchmarking module, we conducted a benchmarking study focused on global haplotype reconstruction (Section 3.3 below).

2.2 Scalability

Scalable workflows can handle and process increasing amounts of data without compromising on performance or efficiency. To achieve scalability, we utilize efficient programming techniques to execute jobs on a computing cluster, ensuring optimal performance. For example, we dynamically specify cluster resources to adapt to the specific data requirements, facilitating smoother deployment on new cluster environments and enable the parallel execution of unrelated data analysis steps. Furthermore, we validate user configuration files using JSON Schema [21] during startup to identify potential runtime errors early. Lastly, we split centralized tasks among multiple compute nodes and perform per-sample distributed computation of summary statistics. In order to make large-scale analyses of public data sets easier, V-pipe 3.0 includes an input data retrieval functionality which requires a set of SRA accession numbers [13] as input and automatically downloads all data files needed to run the whole workflow. Further, scripts are available which facilitate the unattended mass-import of raw files as produced by Illumina's demultiplexing software into the structure that V-pipe 3.0 expects as input. To help with common post-processing steps, we have added scripts to facilitate SRA and GISAID database upload of compressed raw reads and of generated consensus sequences, including the summary quality reports assessing the plausibility of frameshift-causing insertions and deletions. With these features, V-pipe 3.0 has been shown to handle more than 100,000 samples efficiently [22–25].

2.3 Adaptability

Adaptability refers to making it easy for other researchers to build upon an existing workflow and extend it for their application- and domain-specific needs. To demonstrate the ease with which new software components and scrips can be introduced we added two methods for viral diversity estimation: first, PredictHaplo [26] a well-performing global haplotype reconstruction method, and second, a script for the

computation of within-sample diversity indices [27], including Shannon Entropy and population nucleotide diversity. The indices are often applied to compare diversity between samples and have been used for the estimation of time since infection [28]. The addition of new methods requires only the definition of a Conda environment with the required software dependencies and the definition of a Snakemake rule executing the method or script. Additionally, we run automated integration and unit tests using GitHub Actions workflows [19, 20] on every commit submitted to the repository. This verifies the reliability, consistency and correctness of the overall workflow. We use data sets from different viruses in our tests to make sure that V-pipe 3.0 and the newly added features are running successfully from start to end.

Further, V-pipe 3.0 can be easily optimized for different viruses through its configuration setup. The base configuration is virus-agnostic while virus-specific settings (specific reference sequences, different alignment tools, etc.) can be easily plugged in. This allows a quick adaptation of V-pipe 3.0 to any virus, without requiring complex workflow changes. For example, we offer specific configuration setups for HIV, SARS-CoV-2, Poliovirus, Herpes simplex virus, Influenza A virus, Respiratory syncytial virus B, and Drosophila C virus. These setups include selection of suitable reference files, read alignment software, and post-processing steps for each target virus. We demonstrate how to write such configuration files through the example of monkeypox in Figure 2. The configuration defines which alignment and diversity estimation method should be applied, which reference should be used, and which outputs and processing steps should be run. Further, for each method, users can specify the parameter choices.

2.4 Transparency

Transparency refers to the ability to easily comprehend a given workflow. This is particularly crucial for ensuring interpretability and facilitating efficient collaboration in

large-scale projects with many stakeholders. The documentation of V-pipe 3.0 is written in the form of dynamic scripts which allows testing of the configuration options in an automated fashion and making sure they always represent the latest release version and do not contain outdated information. Additionally, V-pipe 3.0 offers a range of tutorials that cover various applications, including the processing of SARS-CoV-2 or HIV samples, as well as a tutorial specifically designed for processing wastewater samples [29].

In order to facilitate prompt user access to new functionalities and accelerate the onboarding process for new users, we provide four deployment options: (1) a Bash script which automatically creates the required Conda environments, installs all dependencies, and initializes a project structure, (2) Snakemake's snakedeploy tool to install V-pipe 3.0 in the standardized Snakemake fashion, (3) a Docker container [30] which is automatically generated for every new release and for the master branch of the GitHub repository, and (4) execution within a workflow execution service, such as Sapporo [31], by fetching V-pipe from a tools repository service, such as WorkflowHub [32]. Further, the configuration definition summarizes the steps of the workflow in one single file and hence also facilitates information sharing between collaborators.

3 Applications

The development of V-pipe 3.0 was primarily driven by two large-scale SARS-CoV-2 surveillance projects. Continuous updates and extensions of the pipeline have been motivated by the evolving demands of the pandemic. With each workflow update, the entire sample cohort underwent reanalysis using the latest pipeline version. This approach guaranteed consistent results that align with the most recent advancements in the pipeline. In the following, we present how sustainable data processing using V-pipe 3.0 was key to the successful execution of the two surveillance projects,

and we demonstrate the benchmarking module by conducting a global haplotype reconstruction benchmarking study.

3.1 Swiss SARS-CoV-2 Sequencing Consortium

In the scope of the Swiss SARS-CoV-2 Sequencing Consortium [33], V-pipe 3.0 was consistently utilized to process sequencing data and generate consensus sequences. This continuous usage began with the first consortium sequencing run on 23 April 2020, and concluded when the consortium was dissolved in January 2023. V-pipe 3.0 demonstrated its adaptability by transitioning from its original focus on HIV to processing samples from SARS-CoV-2. The first Swiss SARS-CoV-2 case was reported on 25 February 2020 [34], and we submitted the first sequence processed by V-pipe 3.0 to GISAID on 25 May 2020 (accession number: EPI_ISL_451681, sampled on 12 March 2020). The fast development and changing demands in the SARS-CoV-2 pandemic required the rapid development of new tools that had to be integrated in the processing pipeline, for example, the frameshift insertion/deletion checks mentioned before. Apart from adaptability, portability and reproducibility were essential for this project, as it involved analyses conducted by different individuals from various academic groups on their own computing facilities. Since the consensus sequences and their Pango lineage [35] designations were reported to the Swiss Federal Office of Public Health to inform public health decision-making, reproducibility was essential to guarantee reliable, consistent, and trustworthy results. Further, scalability to maximize the use of computational resources made it possible to handle the large amounts of clinical SARS-CoV-2 samples throughout the pandemic [36], which resulted in 74,409 consensus sequences being submitted to GISAID [37] as of 21 Sep 2023 (accessed 21 Sep 2023). At the peak of our efforts, V-pipe 3.0 processed up to 1500 clinical samples on a weekly basis (Figure 3A), providing a substantial part to the national surveillance efforts of circulating SARS-CoV-2 variants in Switzerland [22–24].

3.2 Swiss surveillance of SARS-CoV-2 genomic variants in wastewater

Another successful application of V-pipe 3.0 has been the Swiss surveillance of SARS-CoV-2 genomic variants in wastewater [38] (Figure 3C). Wastewater samples contain mixtures of multiple SARS-CoV-2 lineages, and hence workflows targeting diversity analysis are prime candidates for handling them. V-pipe 3.0 was used to analyze the sequencing data and to estimate the abundances of the circulating SARS-CoV-2 variants in Switzerland. In particular, the wastewater analysis enabled the early detection of new variants of concern such as Alpha (B.1.1.7) [6]. Starting in December 2020, V-pipe 3.0 has been continuously used to process wastewater samples from 6-10 different locations 3-7 times per week [38] (Figure 3B). Since then, V-pipe 3.0 has been the core of the automated monitoring of the circulating SARS-CoV-2 genomic variants in Switzerland (Figure 3C). As of 23 May 2024, 7785 samples have already been submitted to the ENA project (PRJEB44932).

The complexity of the SARS-CoV-2 variant mixtures in wastewater samples required additions to the standard workflow, namely primer trimming and the newly developed methods COJAC [6] and LolliPop [39] for variant detection and time-series deconvolution of the variant mixtures, respectively. The modular and standard Snakemake structure of V-pipe 3.0 facilitated the integration of the new functionalities through adding new Snakemake rules for their execution. Lastly, the involvement of the large number of stakeholders and collaborators in the surveillance consortium of SARS-CoV-2 genomic variants in wastewater required transparency of the whole analysis pipeline. All stakeholders and developers had to be aware of the functionalities and steps of the data processing. This was possible through the modular structure and the clear configuration files used by V-pipe 3.0, as well as the fact that all parts of the pipeline are open source and their configuration automatically documented.

3.3 Global haplotype reconstruction benchmark

To showcase the strengths of V-pipe 3.0's benchmarking module, we designed a global haplotype reconstruction benchmarking study. Global haplotype reconstruction is a useful methodology in genetic research as it allows for a comprehensive understanding of the underlying genetic variations within a population. Due to the computational challenges involved in global haplotype reconstruction [40], it serves as a valuable application for the benchmarking module. Additionally, this benchmarking study provides an opportunity to evaluate new methods that could potentially be included in V-pipe 3.0. In our study, we compared the performance of the probabilistic method PredictHaplo and the graph-based methods CliqueSNV, HaploConduct, and HaploClique. We setup the benchmarking such that the methods were tested on two synthetic data sets and on one real data set.

Using the integrated synthetic data generation component of the module, we consider a genome of length $10,000$ bp, generate a population of 10 haplotypes (Population 1) and simulate Illumina reads of length 200bp (Section 7.2). We vary the coverage between $500, 1000, 5000, 10,000$ in order to investigate how well the methods are able to recover low-frequency haplotypes as the total coverage decreases.

We observe that PredictHaplo achieves perfect precision of 1 in all cases, CliqueSNV's mean precision is between 0.60 and 0.68 with a slight increase with higher coverage (Figure 4A). In terms of recall, CliqueSNV features the highest recall of $0.5 - 0.6$ which remains constant over all coverage values, while PredictHaplo's recall increases up to 0.30 for the highest coverage of $10,000$. Consequently, the recall performance of CliqueSNV is less dependent on the coverage level when compared to PredictHaplo. Across all coverage values, CliqueSNV and PredictHaplo consistently achieve N50 scores of $10,000$, covering the entire genome length. In contrast, both HaploClique and HaploConduct fail to cover even a quarter of the genome, and show a precision and recall of 0 in all cases. This indicates that all sequences predicted by

HaploClique and HaploConduct have relative edit distance greater than 0.01 to any true haplotype, and no true haplotypes are recovered. The poor performance (according to this measure) could be attributed to HaploClique being executed with restricted clique size and maximal clique size parameters, which may not be adequate for the assembly of longer regions. This parameter choice was necessary to prevent excessively long runtime and memory consumption. For all methods, we see a general trend of growing runtime with increasing coverage. CliqueSNV consistently requires the least amount of time to run, while PredictHaplo needs over an hour for the highest coverage (Figure 4A).

By varying the haplotype population in terms of number of haplotypes and pairwise distance while keeping the coverage constant, we generate five additional haplotype populations (populations 2-6, Figure 5C). Across all populations, we again observe perfect precision of 1 for PredictHaplo. For populations 3 and 4, CliqueSNV has nearly perfect precision of $0.83 - 1$. However, CliqueSNV is only able to detect haplotypes from the larger group of 20 haplotypes. Both CliqueSNV and PredictHaplo obtain their highest recall for populations 1 and 2 (Figure 4B), which are the two populations with only 10 haplotypes, and their lowest recall for populations 5 and 6 each with 55 haplotypes. This indicates that both tools are not able to appropriately deal with large haplotype populations. As before, CliqueSNV's generally higher recall than PredictHaplo's, is due to CliqueSNV predicting a larger amount of haplotypes than PredictHaplo. In all simulated populations, we observe that PredictHaplo predicts a single haplotype per cluster while CliqueSNV finds, if any, always multiple ones per cluster (Figure 4B). HaploClique and HaploConduct remain at a recall and precision of 0.

Next, we used the experimental HIV-5-strain mixture [15] to evaluated the methods on a real sequencing data. We observe that precision and recall remain in the range of $0.2 - 0.4$ for PredictHaplo. CliqueSNV and HaploConduct remain at 0 for precision

and recall. As before, PredictHaplo's and CliqueSNV's reconstructions cover nearly the whole genome while HaploConduct covers less than a fifth (Figure 4C).

In summary, our benchmarking studies demonstrates that CliqueSNV exhibits the shortest runtime and delivers the highest recall performance for the simulated samples, whereas PredictHaplo exhibits superior precision for the same samples. This can mostly be explained by CliqueSNV typically recovering a larger amount of haplotypes than PredictHaplo. PredictHaplo was better able to reconstruct global haplotypes with the real data set both in terms of precision and recall. Overall, the results of our benchmark study indicate that the performance of all methods is diverse and highlights the need of continuous benchmarking as new methods are developed.

The benchmarking study can be effortlessly reproduced due to its adherence to Snakemake guidelines. It can be easily customized for different scenarios by integrating a novel data generation script. Moreover, incorporating new methods into the study merely requires adding a short script to execute those methods. Thus, our benchmarking study itself aligns with sustainable data processing practices.

4 Comparison to other workflows

We compare V-pipe 3.0 to other relevant viral bioinformatics pipelines for within-sample diversity estimation, focusing on functionalities and sustainability (Table 2). The compared pipelines include nf-core/viralrecon [10], HAPHPIPE [11], ViralFlow [9] and the pipeline of the GalaxyProject SARS-CoV-2 analysis effort [41–43]. These pipelines are all open source, actively maintained, and provide within-sample diversity estimates for Illumina sequencing reads. Active maintenance is crucial in this rapidly evolving field as even frequently used methods are still in continuous development and contain bugs for corner cases that only become evident with the rise of massive data sets in recent years.

During the SARS-CoV-2 pandemic many processing pipelines have been developed, however the vast majority of those are specific to SARS-CoV-2, tailored to the ARTIC protocol [44] combined with Illumina sequencing, and only aim to produce consensus sequences. Since SARS-CoV-2 has limited genetic diversity and a well-known reference sequence, these pipelines cannot be easily adapted for the general case.

ViralFlow and the pipeline of the GalaxyProject SARS-CoV-2 analysis effort, however, also provides variant calling and downstream analysis for SARS-CoV-2 lineage assignment. In terms of functionality, all data processing pipelines enable *de novo* assembly, except for ViralFlow and the pipeline of the GalaxyProject SARS-CoV-2 analysis effort. HAPHPIPE and nf-core/viralrecon use SPAdes [45] for this purpose, while V-pipe 3.0 utilizes Vicuna [46]. For read alignment, consensus sequence generation, and single nucleotide variant calling, each pipeline offers different combinations of tools and methods. For instance, both ViralFlow and nf-core/viralrecon provide the option to use iVar's variant calling and consensus sequence generation [47]. HAPHPIPE uses GATK for variant calling [48], the pipeline of the GalaxyProject SARS-CoV-2 analysis effort uses LoFreq [49] for variant calling for Illumina and Medaka [50] for Nanopore sequencing data, and V-pipe 3.0 integrates three mutation callers: LoFreq [49], VILOCA [51] and ShoRAH [52], where ShoRAH and VILOCA also provides local haplotypes. V-Pipe 3.0 stands out with its integrated benchmarking module (Table 2, Section 7.1). This framework allows for simulation of sequencing reads from flexible haplotype populations and performance evaluation of various methods. In contrast, [53] presented a benchmarking workflow for a global haplotype caller that is not easily adaptable due to hard-coded simulation parameters in bash-scripts.

Apart from its functionalities, sustainability is an essential factor for data analysis of enduring impact. V-pipe 3.0, ViralFlow, nf-core/viralrecon and the GalaxyProject SARS-CoV-2 analysis effort ensure reproducibility and portability by providing software dependency definitions, automatically installing all necessary dependencies

upon pipeline installation or execution (Table 2). HAPHPIPE, on the other hand, requires manual installation of some software dependencies. In addition, V-pipe 3.0, ViralFlow, and nf-core/viralrecon offer container services like Docker, ensuring full pipeline portability and reproducibility (Table 2). All five pipelines are transparent and open source, utilizing publicly available tools and methods. They provide documentation for installation and execution. In addition, HAPHPIPE and V-pipe 3.0 offer tutorials and examples to aid users in applying the pipelines to their data. Both nf-core/viralrecon and V-pipe 3.0 have code structures that conform to recommended standards for Nextflow and Snakemake workflows, ensuring code readability for external users, which makes adding new features straightforward. ViralFlow and HAPHPIPE follow more custom code structures, making it challenging to add new features or modify the workflow, thus limiting their adaptability.

Overall, with their portability, automatic tests and gold standard code structure, the workflows V-pipe 3.0, nf-core/viralrecon, and the pipeline of the GalaxyProject SARS-CoV-2 analysis effort can provide sustainable data processing and analysis. While V-pipe 3.0 not only provides additional options for downstream analysis like analysis of co-occurrence of mutations on amplicons (COJAC), or kernel-based deconvolution of time-series mutation frequencies into variants (LolliPop), it also integrates the largest selection of tools for each processing step to ensure suitable processing for different samples. For example, for alignment V-pipe 3.0 supports BWA MEM [54], Bowtie 2 [55], ngshmmalgin [8] and minimap2 [56] which allows the processing of samples with very high diversity regions.

5 Discussion

We have presented V-pipe 3.0, a sustainable data analysis pipeline designed for analyzing next-generation sequencing data of viral genomes and inferring the genomic diversity of intra-host or environmental samples. V-pipe 3.0 has been designed to

be reproducible by following Snakemake's best-practice guidelines, adaptable by implementing virus-specific configuration files which can be quickly exchanged, and transparent by providing automatically tested usage examples, which are available online. We have demonstrated the effectiveness and utility of these developments by highlighting its application to two large-scale projects, where V-pipe 3.0 was used in a production setting to process thousands of samples over multiple years.

A core functionality of V-pipe 3.0 is the estimation of viral diversity from NGS data. A multitude of viral diversity estimation tools exist, making it challenging for users to determine the appropriate tool for their samples. Additionally, the choice of method depends on the desired downstream analysis of the results. To address this challenge, we have developed a versatile benchmarking module that facilitates the continuous assessment of the performance and limitations of existing diversity estimation methods. As this field is still quickly advancing, continuous benchmarking of new and established methods is needed. For this purpose, we focus on making the addition of new tools and test data sets to the workflow as straightforward as possible. Adding new methods is as easy as writing a single script which defines how to execute the tool and how to install it. New data sources can be either synthetic or derived from real experimental samples. In the synthetic case, different haplotype evolution modeling assumptions can be specified in a flexible way. Real data sources can be automatically downloaded and pre-processed as part of the workflow.

Given the mixed performance observed in our benchmark study for global haplotype reconstruction, it is evident that the current methods may not satisfy the demands of downstream applications. The issues with performance can be attributed not only to the limitations of inference methods but also to the complex population structures inherent to viruses. Consequently, the practical application of global haplotype reconstruction is heavily constrained by these poor performing and often non-scalable

methods, and would require improved scalable methods that explicitly account for the uncertainty of the results.

When comparing V-pipe 3.0 to other pipelines with similar purposes we found that, apart from V-pipe 3.0, only nf-core/viralrecon provides sustainable data processing taking into account reproducibility, portability, adaptability and transparency by following Nextflow's best-practice guidelines. V-pipe 3.0 sets itself apart from the other pipelines by offering a broader range of integrated tools and functionalities, supported by thorough documentation and tutorials that address various application settings.

6 Conclusions

In summary, we have developed V-pipe 3.0 a sustainable data analysis pipeline for within-sample diversity estimation that can be easily applied to large numbers of samples by other researchers while keeping its execution robust and its workflow structure open to modifications. We have created a benchmarking module for one of V-pipe 3.0's core functionalities which can be continuously updated when new methods and data sets appear. By continuing our close interactions and exchange with users through our mailing list, active GitHub discussions and workshops, we will further expand V-pipe 3.0 to support different kinds of sequencing data, make it more robust to unpredictable failure points in cluster environments and further improve interoperability with data providers and users.

7 Methods

In the following, we introduce V-pipe 3.0's benchmarking module and its application to the global haplotype reconstruction benchmarking study in detail.

7.1 Benchmarking module

V-pipe 3.0's benchmarking module allows the benchmarking of global haplotype reconstruction methods on real and simulated data. For simulated data the workflow consists of four steps: generation of haplotype populations, shotgun read simulation, methods execution and performance evaluation (Figure 5A). In the case of real data, the first two steps are replaced by a data downloading and alignment step.

Generation of synthetic data sets

The synthetic data sets are generated in two steps. First, viral haplotype populations are generated. In the second steps, reads are simulated (Figure 5A). If no reference sequence is provided by the user, it is generated by drawing bases uniformly at random for each position based on the user-provided genome length.

We integrated two options for the viral haplotype population generation based on user-specified mutation rates or pairwise distances. Incorporating new methods involves the addition of a new script to the module, which generates haplotypes in fasta format as output. In the case of haplotype generation based on mutation rates, substitutions, deletions and insertions are randomly introduced into the master sequence based on the user-specified rates μ. The frequency composition of those haplotypes in the population is derived from haplotype frequencies $f = (f_1, ..., f_K)$ provided by the user. These simulation settings allow testing the reconstruction limits of the different viral diversity estimation methods.

In the case of haplotype generation by pairwise distances, we simulate hierarchical relationships among the haplotypes by generating two groups of closely related haplotypes that share a common ancestor (Figure 5B). First, using the user-specified between-group pairwise distance d_{12} two haplotypes are generated from the reference sequence. Second, for each haplotype, child-haplotypes are generated by introducing

mutations based on the respective within-group pairwise distance (d_1 and d_2 respectively) and group size (n_1 and n_2 respectively). The frequency distribution of the generated haplotypes is obtained from a geometric series with a given ratio (default: 0.75), this results in a few high-frequency and many low-frequency haplotypes being present. Additionally the frequency distribution can also be drawn from a Dirichlet distribution with user-povided concentration parameters α_i.

Given a user-specified per-position coverage and read length, paired-end reads are simulated in shotgun-mode using the ART Illumina read simulator ([57], RRID: SCR_006538).

Integration of real data sets

In addition to synthetic data sets where the ground truth is known, real data sets are included in the benchmark. We test the global haplotype reconstruction methods on sequencing reads from the 5-virus-mix presented in [15] (SRA accession number: SRX342666). It provides Illumina MiSeq reads for a mixture of five HIV-1 strains: HXB2, 89.6, JR-CSF, NL4-3 and YU-2 and thus gives an estimate of the ground truth which can be used for performance evaluation. The benchmark workflow is designed to make the addition of further real data sets easily possible.

Performance evaluation

To evaluate the performance of each method in the global haplotype reconstruction benchmark, we compute precision and recall for the recovery of ground truth global haplotypes for each method in each condition. To do so, we consider the ground truth set of haplotype sequences and the set of sequences produced by a method. For each predicted sequence, we check if there exists a ground truth sequence with a relative edit

distance below a predefined threshold γ. We define the relative edit distance ED_{rel} as

$$ED_{rel} = \frac{ED}{\max(L_{pred}, L_{true})} \quad (1)$$

where ED is the edit distance between a predicted and ground truth haplotype which have lengths L_{pred} and L_{true} respectively. If $ED_{rel} < \gamma$, the predicted haplotype counts as a true positive, otherwise as a false positive. To compute the number of false negatives, we iterate over all ground truth sequences. We count a false negative if a ground truth sequence has no matching, i.e., relative edit distance below a certain threshold, predicted sequence. From this, we compute precision as $TP/(TP + FP)$ and recall as $TP/(TP+FN)$. We use $\gamma = 0.01$ as the relative edit distance threshold in the benchmark study.

Two-dimensional embeddings of haplotype sequences are generated by applying multidimensional scaling with precomputed edit distances between all sequences [58].

We use MetaQUAST to compute measures of assembly quality for the reconstructed haplotypes [59]. In particular, we compute the N50 score which, in this context, equals the length of the shortest haplotype, which together with all larger haplotypes, covers at least half the genome.

7.2 Global haplotype reconstruction benchmark study

We used the benchmarking module to benchmark global haplotype reconstruction methods. The scripts to reproduce the benchmarking study are available on GitHub [60].

Datasets

We generated two synthetic data sets applying the distance-based haplotype generation mode and used one real data set. In the first synthetic data set, we considered a genome of length 10000 with reads of length 200. We then generated two groups of

haplotypes such that group one has size $n_1 = 5$ and group two has size $n_2 = 5$, the average pairwise sequence distance within group one is $d_1 = 50$, the average pairwise sequence distance within group two is $d_2 = 20$, and the average pairwise sequence distance between the two groups is $d_{12} = 200$. We varied the coverage between $500, 1000, 5000, 10000$ in order to investigate how well the methods are able to recover low-frequency haplotypes as the coverage decreases. In the second synthetic data set, we considered a genome of length 10000 with reads of length 200 at a constant coverage of 1000. We then used the six haplotype population parameter settings as specified in Figure 5C in order to investigate how well the methods are able to recover different types of haplotype populations with different diversity levels. For the real data set, we used the 5-virus-mix which contains the HIV-1 strains HXB2, 89.6, JR-CSF, NL4-3 and YU-2 mixing in uniform proportions.

Global haplotype methods

We considered all methods discussed in [40] for which a Conda package is available. They are aBayesQR [61], CliqueSNV [62], HaploClique [63], HaploConduct [64], PEHaplo [65], PredictHaplo [26], QuasiRecomb [66], and RegressHaplo [67]. From the benchmark study we excluded aBayesQR because the program failed to parse the input sequencing reads, PEHaplo because it failed execution during the result assembly, QuasiRecomb as it terminated during startup and Regresshaplo, because not all dependencies of its Conda package were available. The remaining tools are HaploConduct, HaploClique, PredictHaplo and CliqueSNV which are all reference-based global haplotype reconstruction methods. This means that they rely on the existence of a viral reference sequence which is similar to the haplotypes expected to occur. The input reads are then typically mapped against this reference sequence which makes reconstructing global haplotypes easier, because read positions relative to the genome

are available, but also introduces a bias, as haplotypes which are dissimilar to the reference might not be captured. For the real data set, we had to exclude HaploClique for its excessive memory consumption.

Availability of Source Code and Requirements

- Project name: V-pipe
- Project home page: https://github.com/cbg-ethz/V-pipe
- Operating system(s): Platform independent
- Programming language: Python, Shell, CSS, Dockerfile, Jupyter Notebook
- License: Apache-2.0 license
- RRID: SCR_025399
- Biotools: biotools:v-pipe
- WorkflowHub DOI: 10.48546/WORKFLOWHUB.WORKFLOW.301.5

Data Availability

All supporting data and materials are available in the Software Heritage [68]. This includes details on how to reproduce the synthetic benchmark datasets and all the scripts to run the workflow. The sequencing data of the 5-virus-mix used in the global haplotype reconstruction is accessible on the Sequence Read Archive under the accession number SRX342666. The SARS-CoV-2 consensus sequences generated are available on GISAID with GISAID Identifier: EPI_SET_231013cd. The samples from the Swiss SARS-CoV-2 Wastewater Surveillance have been submitted to the ENA Project under the project id: PRJEB44932.

Competing interests

The authors declare that they have no competing interests.

Funding

LF was funded by European Union's Horizon 2020 research and innovation program, under the Marie Skłodowska-Curie Actions Innovative Training Networks grant agreement no. 955974 (VIROINF).

Authors' contributions

LF, KPJ, IT and NB worked on the conceptualization and design of the pipeline. IT, KJP, LF, AAB, NBorg, PIB, MC, CC, AD, MD, DD, AJ, BL, MO and US were involved in implementing or adding new methods or tools. KJP conducted the benchmark study. CC, DD, IT, LdP, TS, MC, FS, NB, LF, and KPJ were involved in the analysis and processing of the SARS-CoV-2 clinical and wastewater samples. DD, IT, NB, KJP, LF were involved in the visualization of the results. KPJ and LF were writing the original draft. NB, LdP, TS, FS were involved in reviewing and editing of the manuscript. All authors read and approved the final manuscript.

Acknowledgements

We gratefully acknowledge all data contributors, i.e., the authors and their originating laboratories responsible for obtaining the specimens, and their submitting laboratories for generating the genetic sequence and metadata and sharing via the GISAID Initiative [69].

References

[1] Pereira R, Oliveira J, Sousa M. Bioinformatics and computational tools for next-generation sequencing analysis in clinical genetics. Journal of clinical medicine. 2020;9(1):132.

[2] Barzon L, Lavezzo E, Costanzi G, Franchin E, Toppo S, Palù G. Next-generation sequencing technologies in diagnostic virology. Journal of Clinical Virology. 2013;58(2):346–350.

[3] Capobianchi M, Giombini E, Rozera G. Next-generation sequencing technology in clinical virology. Clinical Microbiology and Infection. 2013;19(1):15–22.

[4] Ko HY, Li YT, Chao DY, Chang YC, Li ZRT, Wang M, et al. Inter-and intra-host sequence diversity reveal the emergence of viral variants during an overwintering epidemic caused by dengue virus serotype 2 in southern Taiwan. PLoS neglected tropical diseases. 2018;12(10):e0006827.

[5] Bonnaud EM, Troupin C, Dacheux L, Holmes EC, Monchatre-Leroy E, Tanguy M, et al. Comparison of intra-and inter-host genetic diversity in rabies virus during experimental cross-species transmission. PLoS pathogens. 2019;15(6):e1007799.

[6] Jahn K, Dreifuss D, Topolsky I, Kull A, Ganesanandamoorthy P, Fernandez-Cassi X, et al. Early detection and surveillance of SARS-CoV-2 genomic variants in wastewater using COJAC. Nature Microbiology. 2022;7(8):1151–1160.

[7] Hillary LS, Maher KH, Lucaci A, Thorpe J, Distaso MA, Gaze WH, et al. Monitoring SARS-CoV-2 in municipal wastewater to evaluate the success of lockdown measures for controlling COVID-19 in the UK. Water Research. 2021;200:117214.

[8] Posada-Céspedes S, Seifert D, Topolsky I, Jablonski KP, Metzner KJ, Beerenwinkel N. V-pipe: a computational pipeline for assessing viral genetic diversity from high-throughput data. Bioinformatics. 2021;37(12):1673–1680.

[9] Dezordi FZ, Neto AMdS, Campos TdL, Jeronimo PMC, Aksenen CF, Almeida SP, et al. ViralFlow: a versatile automated workflow for SARS-CoV-2 genome

assembly, lineage assignment, mutations and intrahost variant detection. Viruses. 2022;14(2):217.

[10] Patel H, Varona S, Monzón S, Espinosa-Carrasco J, Heuer ML, nf-core bot, et al.: nf-core/viralrecon: nf-core/viralrecon v2.5 - Manganese Monkey. Zenodo. Available from: https://doi.org/10.5281/zenodo.6827984.

[11] Bendall ML, Gibson KM, Steiner MC, Rentia U, Pérez-Losada M, Crandall KA. HAPHPIPE: haplotype reconstruction and Phylodynamics for deep sequencing of Intrahost viral populations. Molecular biology and evolution. 2021;38(4):1677–1690.

[12] Knyazev S, Chhugani K, Sarwal V, Ayyala R, Singh H, Karthikeyan S, et al. Unlocking capacities of genomics for the COVID-19 response and future pandemics. Nature Methods. 2022;19(4):374–380.

[13] Leinonen R, Akhtar R, Birney E, Bower L, Cerdeno-Tárraga A, Cheng Y, et al. The European nucleotide archive. Nucleic acids research. 2010;39(suppl_1):D28–D31.

[14] Benson DA, Cavanaugh M, Clark K, Karsch-Mizrachi I, Lipman DJ, Ostell J, et al. GenBank. Nucleic acids research. 2012;41(D1):D36–D42.

[15] Giallonardo FD, Töpfer A, Rey M, Prabhakaran S, Duport Y, Leemann C, et al. Full-length haplotype reconstruction to infer the structure of heterogeneous virus populations. Nucleic acids research. 2014;42(14):e115–e115.

[16] Mölder F, Jablonski KP, Letcher B, Hall MB, Tomkins-Tinch CH, Sochat V, et al. Sustainable data analysis with Snakemake. F1000Research. 2021;10.

[17] Baker M. 1,500 scientists lift the lid on reproducibility. Nature. 2016;533(7604).

[18] Sayre F, Riegelman A. The reproducibility crisis and academic libraries. College & Research Libraries. 2018;79(1):2.

[19] GitHub Inc.: GitHub Actions Website. Accessed 2023-10-02. Available from: https://github.com/features/actions.

[20] V-pipe version 3.: GitHub Actions workflow scripts for installations and end-to-end tests. Accessed 2024-04-29. Available from: https://github.com/cbg-ethz/V-pipe/tree/master/.github/workflows.

[21] JSON Schema.: Website. Accessed 2023-10-03. Available from: https://json-schema.org/.

[22] Nadeau SA, Vaughan TG, Beckmann C, Topolsky I, Chen C, Hodcroft E, et al. Swiss public health measures associated with reduced SARS-CoV-2 transmission using genome data. medRxiv. 2021;.

[23] Chen C, Nadeau SA, Topolsky I, Manceau M, Huisman JS, Jablonski KP, et al. Quantification of the spread of SARS-CoV-2 variant B. 1.1. 7 in Switzerland. Epidemics. 2021;37:100480.

[24] Chen C, Nadeau SA, Topolsky I, Beerenwinkel N, Stadler T. Advancing genomic epidemiology by addressing the bioinformatics bottleneck: Challenges, design principles, and a Swiss example. Epidemics. 2022;39:100576.

[25] Kuipers J, Batavia AA, Jablonski KP, Bayer F, Borgsmüller N, Dondi A, et al. Within-patient genetic diversity of SARS-CoV-2. BioRxiv. 2020;.

[26] Prabhakaran S, Rey M, Zagordi O, Beerenwinkel N, Roth V. HIV haplotype inference using a propagating dirichlet process mixture model. IEEE/ACM transactions on computational biology and bioinformatics. 2013;11(1):182–191.

[27] Fuhrmann L, Jablonski KP, Beerenwinkel N. Quantitative measures of within-host viral genetic diversity. Current opinion in virology. 2021;49:157–163.

[28] Puller V, Neher R, Albert J. Estimating time of HIV-1 infection from next-generation sequence diversity. PLOS Computational Biology. 2017;13(10):e1005775.

[29] V-pipe version 3.: GitHub directory with tutorials to run V-pipe 3.0. Accessed 2024-04-29. Available from: https://github.com/cbg-ethz/V-pipe/tree/master/docs.

[30] Merkel D, et al. Docker: lightweight linux containers for consistent development and deployment. Linux j. 2014;239(2):2.

[31] Sapporo.: GitHub. Accessed 2023-10-03. Available from: https://github.com/sapporo-wes/sapporo.

[32] WorkflowHub.: Website. Accessed 2023-10-03. Available from: https://workflowhub.eu/.

[33] Swiss SARS-CoV-2 Sequencing Consortium.: Website. Accessed 2022-07-22. Available from: https://bsse.ethz.ch/cevo/research/sars-cov-2/swiss-sars-cov-2-sequencing-consortium.html.

[34] Swiss Federal Office of Public Health.: Press releases, 2020-02-25. Accessed 2023-01-18. Available from: https://www.admin.ch/gov/en/start/documentation/media-releases.msg-id-78233.html.

[35] Rambaut A, Holmes EC, O'Toole Á, Hill V, McCrone JT, Ruis C, et al. A dynamic nomenclature proposal for SARS-CoV-2 lineages to assist genomic epidemiology. Nature microbiology. 2020;5(11):1403–1407.

[36] Chen C, Nadeau S, Yared M, Voinov P, Xie N, Roemer C, et al. CoV-Spectrum: analysis of globally shared SARS-CoV-2 data to identify and characterize new variants. Bioinformatics. 2022;38(6):1735–1737.

[37] Khare S, Gurry C, Freitas L. B Schultz. M, Bach, G, Diallo, A, Akite, N, Ho, J, Tc Lee, R, Yeo, W, Core Curation Team, G, and Maurer-Stroh, S. 2021;p. 1049–1051.

[38] Beerenwinkel N.: Swiss Surveillance of SARS-CoV-2 genomic variants in wastewater. Accessed 2023-01-18. Available from: https://bsse.ethz.ch/cbg/research/computational-virology/sarscov2-variants-wastewater-surveillance.html.

[39] Dreifuss D, Topolsky I, Icer Baykal P, Beerenwinkel N. Tracking SARS-CoV-2 genomic variants in wastewater sequencing data with LolliPop. medRxiv. 2022;p. 2022–11.

[40] Jablonski KP, Beerenwinkel N. Computational Methods for Viral Quasispecies Assembly. In: Virus Bioinformatics. Chapman and Hall/CRC; 2021. p. 51–64.

[41] The Galaxy platform for accessible, reproducible and collaborative biomedical analyses: 2022 update. Nucleic Acids Research. 2022;50(W1):W345–W351.

[42] Baker D, Van Den Beek M, Blankenberg D, Bouvier D, Chilton J, Coraor N, et al. No more business as usual: Agile and effective responses to emerging pathogen threats require open data and open analytics. PLoS pathogens. 2020;16(8):e1008643.

[43] Galaxy.: GalaxyProject SARS-CoV-2 analysis effort workflows. Accessed 2024-04-30. Available from: https://galaxyproject.org/projects/covid19/workflows/.

[44] ARTIC protocol.: Website. Accessed 2023-10-03. Available from: https://artic.network/ncov-2019.

[45] Prjibelski A, Antipov D, Meleshko D, Lapidus A, Korobeynikov A. Using SPAdes de novo assembler. Current protocols in bioinformatics. 2020;70(1):e102.

[46] Yang X, Charlebois P, Gnerre S, Coole MG, Lennon NJ, Levin JZ, et al. De novo assembly of highly diverse viral populations. BMC genomics. 2012;13:1–13.

[47] Grubaugh ND, Gangavarapu K, Quick J, Matteson NL, De Jesus JG, Main BJ, et al. An amplicon-based sequencing framework for accurately measuring intrahost virus diversity using PrimalSeq and iVar. Genome biology. 2019;20(1):1–19.

[48] DePristo MA, Banks E, Poplin R, Garimella KV, Maguire JR, Hartl C, et al. A framework for variation discovery and genotyping using next-generation DNA sequencing data. Nature genetics. 2011;43(5):491–498.

[49] Wilm A, Aw PPK, Bertrand D, Yeo GHT, Ong SH, Wong CH, et al. LoFreq: a sequence-quality aware, ultra-sensitive variant caller for uncovering cell-population heterogeneity from high-throughput sequencing datasets. Nucleic acids research. 2012;40(22):11189–11201.

[50] ONT Research.: medaka: Sequence correction provided by ONT Research. Accessed 2024-05-15. Available from: https://github.com/nanoporetech/medaka.

[51] Fuhrmann L, Langer B, Topolsky I, Beerenwinkel N. VILOCA: Sequencing quality-aware haplotype reconstruction and mutation calling for short- and long-read data. bioRxiv. 2024;https://doi.org/10.1101/2024.06.06.597712.

[52] Zagordi O, Bhattacharya A, Eriksson N, Beerenwinkel N. ShoRAH: estimating the genetic diversity of a mixed sample from next-generation sequencing data. BMC bioinformatics. 2011;12(1):1–5.

[53] Eliseev A, Gibson KM, Avdeyev P, Novik D, Bendall ML, Pérez-Losada M, et al. Evaluation of haplotype callers for next-generation sequencing of viruses. Infection, Genetics and Evolution. 2020;82:104277.

[54] Li H. Aligning sequence reads, clone sequences and assembly contigs with BWA-MEM. arXiv preprint arXiv:13033997. 2013;.

[55] Langmead B, Salzberg SL. Fast gapped-read alignment with Bowtie 2. Nature methods. 2012;9(4):357–359.

[56] Li H. Minimap2: pairwise alignment for nucleotide sequences. Bioinformatics. 2018;34(18):3094–3100.

[57] Huang W, Li L, Myers JR, Marth GT. ART: a next-generation sequencing read simulator. Bioinformatics. 2012;28(4):593–594.

[58] Kruskal JB. Multidimensional scaling by optimizing goodness of fit to a nonmetric hypothesis. Psychometrika. 1964;29(1):1–27.

[59] Mikheenko A, Saveliev V, Gurevich A. MetaQUAST: evaluation of metagenome assemblies. Bioinformatics. 2016;32(7):1088–1090.

[60] V-pipe version 3.: Global haplotype reconstruction benchmarking study. Accessed 2023-10-02. Available from: https://github.com/cbg-ethz/V-pipe/tree/master/resources/auxiliary_workflows/benchmark/resources/multi_setup.

[61] Ahn S, Vikalo H. aBayesQR: a Bayesian method for reconstruction of viral populations characterized by low diversity. In: International Conference on Research

in Computational Molecular Biology. Springer; 2017. p. 353–369.

[62] Knyazev S, Tsyvina V, Shankar A, Melnyk A, Artyomenko A, Malygina T, et al. CliqueSNV: an efficient noise reduction technique for accurate assembly of viral variants from NGS data. bioRxiv. 2020;p. 264242.

[63] Töpfer A, Marschall T, Bull RA, Luciani F, Schönhuth A, Beerenwinkel N. Viral quasispecies assembly via maximal clique enumeration. PLoS computational biology. 2014;10(3):e1003515.

[64] Baaijens JA, Schönhuth A. Overlap graph-based generation of haplotigs for diploids and polyploids. Bioinformatics. 2019;35(21):4281–4289.

[65] Chen J, Zhao Y, Sun Y. De novo haplotype reconstruction in viral quasispecies using paired-end read guided path finding. Bioinformatics. 2018;34(17):2927–2935.

[66] Töpfer A, Zagordi O, Prabhakaran S, Roth V, Halperin E, Beerenwinkel N. Probabilistic inference of viral quasispecies subject to recombination. Journal of Computational Biology. 2013;20(2):113–123.

[67] Leviyang S, Griva I, Ita S, Johnson WE. A penalized regression approach to haplotype reconstruction of viral populations arising in early HIV/SIV infection. Bioinformatics. 2017;33(16):2455–2463.

[68] Fuhrmann L, Jablonski KP, Topolsky I, Batavia AA, Borgsmuller N, Baykal PI, et al .: V-pipe 3.0: a sustainable pipeline for within-sample viral genetic diversity estimation (Version 1). [Computer software]. Software Heritage. . Available from: https://archive.softwareheritage.org/swh:1:snp:a56f8dd7375288732aef62d97beb0675e1a1f422;origin=https://github.com/cbg-ethz/V-pipe.

[69] Elbe S, Buckland-Merrett G. Data, disease and diplomacy: GISAID's innovative contribution to global health. Global challenges. 2017;1(1):33–46.

[70] V-pipe version 3.: Mpox configuration example. Accessed 2023-10-03. Available from: https://github.com/cbg-ethz/V-pipe/blob/master/config/mpxv.yaml.

[71] Cantu VA, Sadural J, Edwards R. PRINSEQ++, a multi-threaded tool for fast and efficient quality control and preprocessing of sequencing datasets. PeerJ Preprints. 2019;7:e27553v1.

[72] Simon Andrews BB.: FastQC version 0.11.9. Accessed 2023-10-02. Available from: https://www.bioinformatics.babraham.ac.uk/projects/fastqc/.

[73] Danecek P, Marshall J, Danecek P, et al. HTSlib: C library for reading/writing high-throughput sequencing data. GigaScience. 2021;10:giab008.

[74] Vasimuddin M, Misra S, Li H, Aluru S. Efficient architecture-aware acceleration of BWA-MEM for multicore systems. In: 2019 IEEE international parallel and distributed processing symposium (IPDPS). IEEE; 2019. p. 314–324.

[75] Li H. A statistical framework for SNP calling, mutation discovery, association mapping and population genetical parameter estimation from sequencing data. Bioinformatics. 2011;27(21):2987–2993.

List of Figures

1 V-pipe 3.0 workflow overview. The data processing pipeline (left) provides four main steps: (1) preprocessing of the raw reads including quality control, (2) multiple sequence alignment, (3) estimation of viral diversity by SNV, local and global haplotype calling, and (4) if applicable, downstream analysis. The V-pipe 3.0 benchmarking module (right) supports the evaluation of viral diversity estimation methods on simulated data and on real experimental data where the ground truth diversity is known by the experimental design. For the simulated samples, first, ground truth haplotype populations are generated and based on those, sequencing reads are simulated. Then, the simulated and real samples are processed by the methods in the study, and last, the predicted viral diversity is compared to the ground truth viral diversity using different metrics for example precision, recall, f1, and N50 score. Left margins: V-pipe 3.0 is designed to facilitate efficient processing on personal computers as well as on computing clusters. It automatically sets up the necessary Conda environments, installs all dependencies, and initializes the project structure. It is also accessible through a Docker container, which includes all software dependencies. 36

2 Example configuration file for monkeypox virus. User-specified aligner, primer trimming method, and the method for the diversity estimation are defined in the `general` section. Input like reference genome, primer file, and the directory of the samples are specified in the `input` section. In section `preprocessing`, extra command line parameters are passed to the preprocessing step. In section `output`, users can define their desired output of the pipeline. This example configuration file is available on GitHub [70]. 37

3 Swiss surveillance of SARS-CoV-2 genomic variants using V-Pipe 3.0. **A)** Number of weekly submissions of SARS-CoV-2 consensus sequences from clinical samples to GISAID. **B)** Surveillance of SARS-CoV-2 variants in wastewater samples from ten locations in Switzerland with relative abundances of variants. **C)** Time-series of relative variant abundances with 95% confidence bands of wastewater samples from Zurich using V-pipe 3.0. 38

4 Benchmarking study for global haplotype reconstruction methods. **A)** Precision, recall, N50 score, and runtime for simulated samples of varying coverage of population 1. **B)** Left: MDS plots of one example simulation replicate per haplotype population. Each point represents a sequence. Symbol size corresponds to the frequency of the respective haplotype in the sample. HaploClique and HaploConduct were excluded due to their poor performance. Right: Precision and recall plots for each haplotype population. Each marker represents one replicate sample. **C)** N50, precision, recall, and f1 for PredictHaplo, CliqueSNV, and HaploConduct on a real HIV-5-virus mix. 39

5 **A)** Workflow for the performance evaluation of global haplotype reconstruction methods: 1. Generation of haplotype population based on user input, 2. Simulation of paired-end Illumina sequencing reads, 3. Run global haplotype reconstruction methods, 4. Performance evaluation. **B)** Generation of distance based haplotype populations: n_1: number of haplotypes in group one; n_2: number of haplotypes in group two; d_{12}: average pairwise distance between group one and two; d_1: average pairwise sequence distance within group one; d_2: average pairwise sequence distance within group two. **C)** Haplotype population parameter settings for the second synthetic dataset with constant coverage of 1000, and genome of length 10000. 40

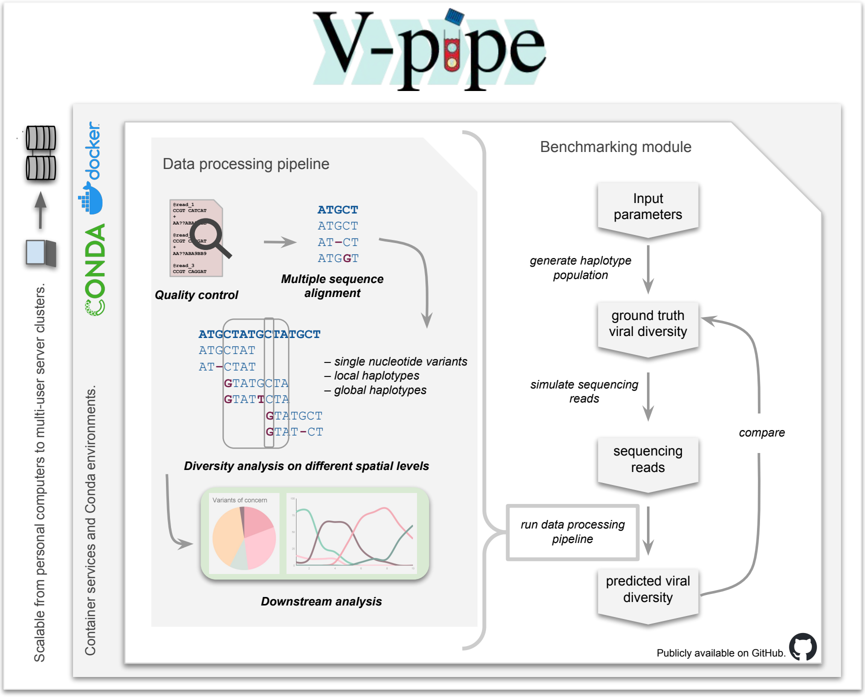

Fig. 1: V-pipe 3.0 workflow overview. The data processing pipeline (left) provides four main steps: (1) preprocessing of the raw reads including quality control, (2) multiple sequence alignment, (3) estimation of viral diversity by SNV, local and global haplotype calling, and (4) if applicable, downstream analysis. The V-pipe 3.0 benchmarking module (right) supports the evaluation of viral diversity estimation methods on simulated data and on real experimental data where the ground truth diversity is known by the experimental design. For the simulated samples, first, ground truth haplotype populations are generated and based on those, sequencing reads are simulated. Then, the simulated and real samples are processed by the methods in the study, and last, the predicted viral diversity is compared to the ground truth viral diversity using different metrics for example precision, recall, f1, and N50 score. Left margins: V-pipe 3.0 is designed to facilitate efficient processing on personal computers as well as on computing clusters. It automatically sets up the necessary Conda environments, installs all dependencies, and initializes the project structure. It is also accessible through a Docker container, which includes all software dependencies.

```yaml
name: MPXV

general:
    aligner: bwa
    primers_trimmer: samtools
    snv_caller: lofreq

input:
    reference: "{VPIPE_BASEDIR}/../resources/mpxv/MT903345.1.fasta"
    primers_file: "{VPIPE_BASEDIR}/../resources/mpxv/primers/MPXV-primer_genome-positions_subset.tsv"
    primers_bedfile: "{VPIPE_BASEDIR}/../resources/mpxv/primers/MPXV-primer_genome-positions_subset.bed"
    datadir: "{VPIPE_BASEDIR}/../resources/samples/"
    samples_file: samples.tsv

preprocessing:
    extra: -ns_max_n 4 -min_qual_mean 20 -trim_qual_left 20 -trim_qual_right 20 -trim_qual_window 10

output:
    trim_primers: true
    snv: true
    local: true
    global: false
    visualization: true
    QA: true
```

Fig. 2: Example configuration file for monkeypox virus. User-specified aligner, primer trimming method, and the method for the diversity estimation are defined in the `general` section. Input like reference genome, primer file, and the directory of the samples are specified in the `input` section. In section `preprocessing`, extra command line parameters are passed to the preprocessing step. In section `output`, users can define their desired output of the pipeline. This example configuration file is available on GitHub [70].

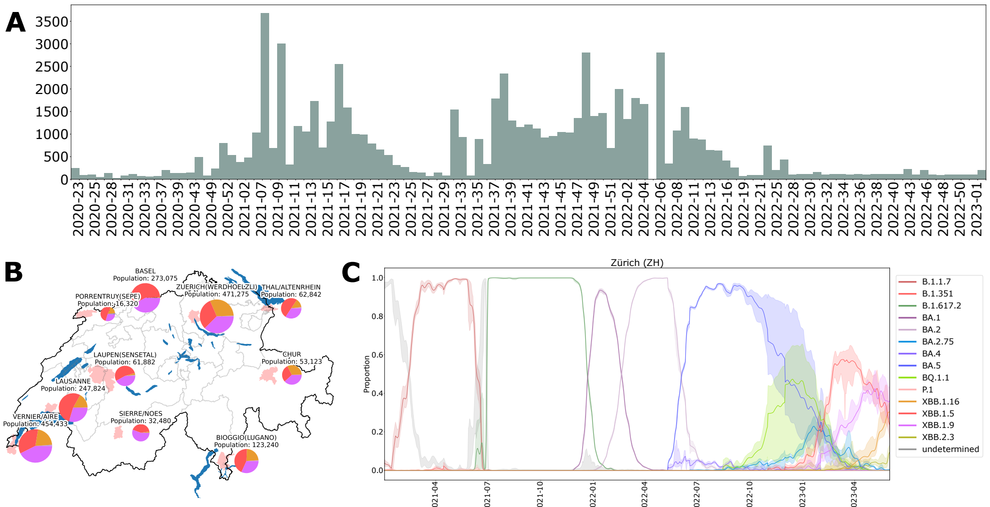

Fig. 3: Swiss surveillance of SARS-CoV-2 genomic variants using V-Pipe 3.0. **A)** Number of weekly submissions of SARS-CoV-2 consensus sequences from clinical samples to GISAID. **B)** Surveillance of SARS-CoV-2 variants in wastewater samples from ten locations in Switzerland with relative abundances of variants. **C)** Time-series of relative variant abundances with 95% confidence bands of wastewater samples from Zurich using V-pipe 3.0.

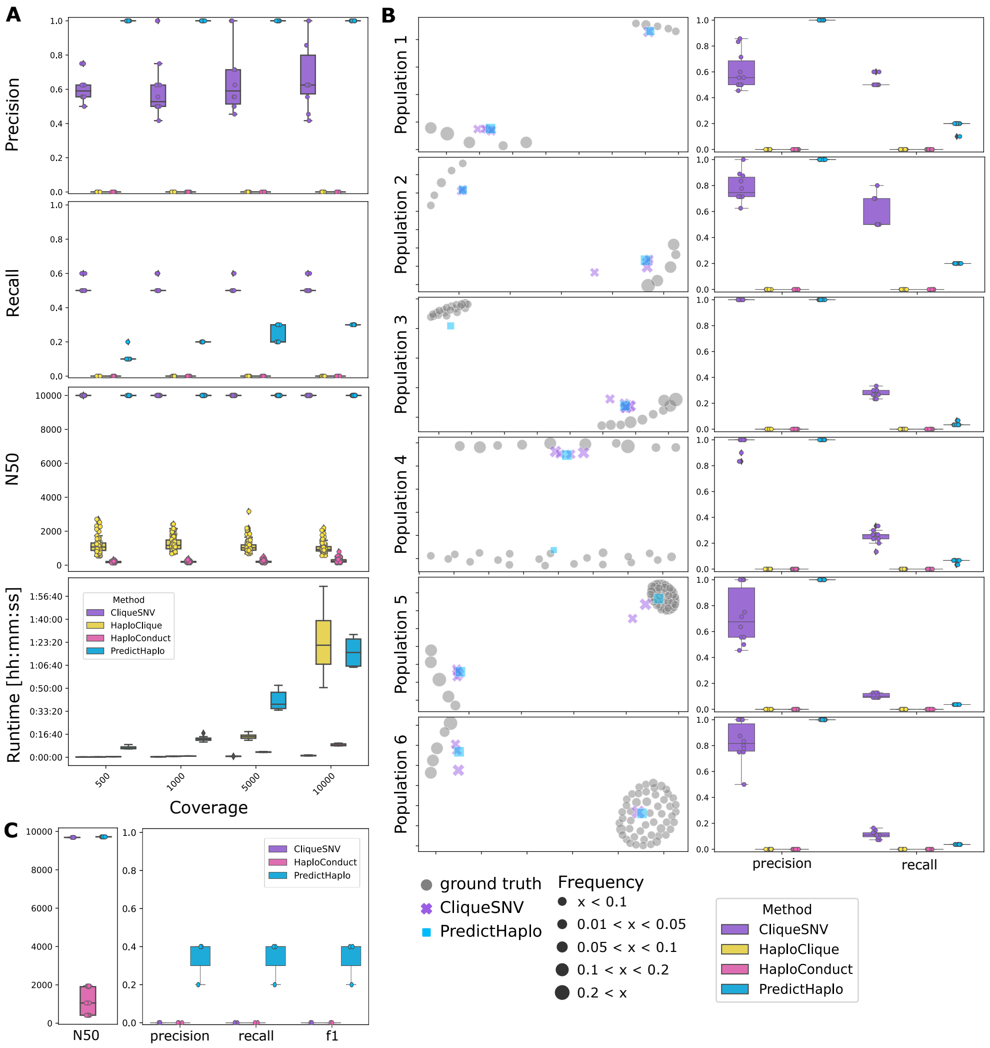

Fig. 4: Benchmarking study for global haplotype reconstruction methods. **A)** Precision, recall, N50 score, and runtime for simulated samples of varying coverage of population 1. **B)** Left: MDS plots of one example simulation replicate per haplotype population. Each point represents a sequence. Symbol size corresponds to the frequency of the respective haplotype in the sample. HaploClique and HaploConduct were excluded due to their poor performance. Right: Precision and recall plots for each haplotype population. Each marker represents one replicate sample. **C)** N50, precision, recall, and f1 for PredictHaplo, CliqueSNV, and HaploConduct on a real HIV-5-virus mix.

A

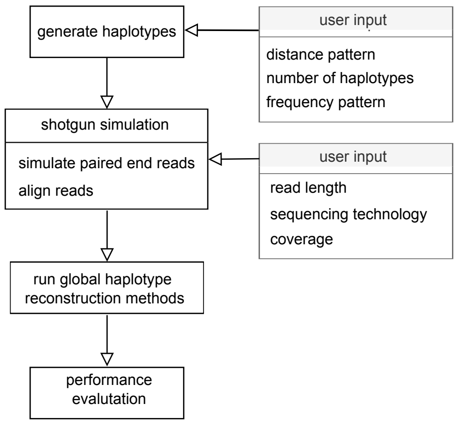

B

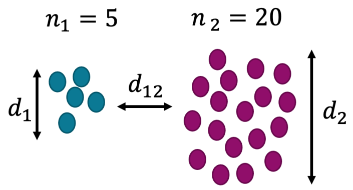

C

Population	n_1	n_2	d_{12}	d_1	d_2
1	5	5	200	50	20
2	5	5	400	100	100
3	10	20	200	50	20
4	10	20	400	100	100
5	5	50	200	50	20
6	5	50	400	100	100

Fig. 5: **A)** Workflow for the performance evaluation of global haplotype reconstruction methods: 1. Generation of haplotype population based on user input, 2. Simulation of paired-end Illumina sequencing reads, 3. Run global haplotype reconstruction methods, 4. Performance evaluation. **B)** Generation of distance based haplotype populations: n_1: number of haplotypes in group one; n_2: number of haplotypes in group two; d_{12}: average pairwise distance between group one and two; d_1: average pairwise sequence distance within group one; d_2: average pairwise sequence distance within group two. **C)** Haplotype population parameter settings for the second synthetic dataset with constant coverage of 1000, and genome of length 10000.

List of Tables

1 Methods and tools per data processing step that are integrated in V-Pipe 3.0 and the previous V-pipe workflow. Newly integrated tools into V-pipe 3.0 are marked in bold. 42
2 Comparison in terms of sustainability and functionalities of viral bioinformatics workflows for within-sample diversity estimation. Asteriks marks differences in the specfic tools integrated in V-pipe and V-pipe 3.0. 43

Data processing task	Tools in V-pipe 3.0	Tools in V-pipe
Quality control	PRINSEQ<br>FastQC<br>**'skip' quality control**	PRINSEQ ([71], RRID: SCR_005454)<br>FastQC ([72], RRID: SCR_014583)
De novo assembly	VICUNA	VICUNA ([46], RRID: SCR_006302)
Primer trimming	**IVar** ([47], RRID: SCR_024045)<br>**SAMtools** ([73], RRID: SCR_002105)	
Aligner	BWA MEM<br>Bowtie 2<br>ngshmmalign<br>**minimap2** ([56], RRID: SCR_018550)	BWA MEM ([74], RRID: SCR_022192)<br>Bowtie 2 ([55], RRID: SCR_016368)<br>ngshmmalign [8]
Paired-end reads merger	**SmallGenomeUtilites** [8]	
Consensus sequence generation	SmallGenomeUtilites<br>ngshmmalign<br>**BCFtools** ([73, 75], RRID: SCR_005227)	SmallGenomeUtilites [8]<br>ngshmmalign [8]
Frameshifts and stop codon diagnostics	**SmallGenomeUtilites** [8]	
Mutation calling	LoFreq<br>ShoRAH<br>**VILOCA** [51]	LoFreq ([49], RRID: SCR_013054)<br>ShoRAH ([52], RRID: SCR_005211)
Local haplotype reconstruction	ShoRAH<br>**VILOCA** [51]	ShoRAH [52]
Global haplotype reconstruction	**PredictHaplo** ([26], RRID: SCR_005207)<br>HaploConduct (SAVAGE)<br>HaploClique<br>**QuasiRecomb** ([66], RRID: SCR_008812)	HaploConduct (SAVAGE) [64]<br>HaploClique [63]
Wasterwater surveillance	**COJAC** [6]<br>**LolliPop** [39]	

Table 1: Methods and tools per data processing step that are integrated in V-Pipe 3.0 and the previous V-pipe workflow. Newly integrated tools into V-pipe 3.0 are marked in bold.

	V-pipe 3.0	V-pipe	ViralFlow	nf-core/viralrecon	HAPHPIPE	GalaxyProject SARS-CoV-2 analysis effort
**Reproducibility**						
Automatic installation of all software dependencies	✓	✓	✓	✓	✗	✓
Container Services (e.g. Docker)	✓	✗	✓	✓	✗	✓
Automatic pipeline installation tests	✓	✗	✗	✓	✗	not applicable
Automatic pipeline execution tests on experimental samples	✓	✗	✗	✓	✗	✓
**Scalability**						
Dynamic cluster resource allocation	✓	✓	✓	✓	✗	✓
**Adaptability**						
Applicable for general viruses	✓	✓	✗	✓	✓	✗
Modular execution	✓	✓	✗	✓	✓	✓
Development: feature adding	✓	✓	✗	✓	✗	✓
**Transparency**						
Open source	✓	✓	✓	✓	✓	✓
Readability: pipeline code structure follow standard	✓	✗	✗	✓	✗	✓
Documentation	✓	✓	✓	✓	✓	✓
Examples	✓	✓	✓	✓	✓	✓
Tutorials	✓	✗	✓	✗	✓	✓
**Functionalities**						
De novo assembly	✓	✓	✗	✓	✓	✗
Read alignment	✓	✓	✓	✓	✓	✓
Consensus sequence generation	✓	✓(*)	✓	✓	✓	✓
Mutation calling	✓	✓(*)	✓	✓	✓	✓
Local haplotype reconstruction	✓	✓(*)	✗	✗	✗	✗
Global haplotype reconstruction	✓	✓(*)	✗	✗	✓	✗
SARS-CoV-2 wastewater surveillance	✓	✗	✓	✗	✗	✓
Benchmarking module	✓	✓	✗	✗	✗	✗

Table 2: Comparison in terms of sustainability and functionalities of viral bioinformatics workflows for within-sample diversity estimation. Asteriks marks differences in the specfic tools integrated in V-pipe and V-pipe 3.0.

Manuscript Click here to access/download;Manuscript;V_pipe 2.pdf

V-pipe 3.0: a sustainable pipeline for within-sample viral genetic diversity estimation

Lara Fuhrmann [1,2†], Kim Philipp Jablonski [1,2†], Ivan Topolsky [1,2†], Aashil A Batavia [1,2], Nico Borgsmüller [1,2], Pelin Icer Baykal [1,2], Matteo Carrara [2,4], Chaoran Chen [1,2], Arthur Dondi [1,2], Monica Dragan [1,2], David Dreifuss [1,2], Anika John [1,2], Benjamin Langer[1], Michal Okoniewski [3], Louis du Plessis [1,2], Uwe Schmitt [3], Franziska Singer [4], Tanja Stadler [1,2], Niko Beerenwinkel [1,2*]

[1]Department of Biosystems Science and Engineering, ETH Zurich, Basel, 4056, Switzerland.
[2]SIB Swiss Institute of Bioinformatics, Lausanne, 1015, Switzerland.
[3]Scientific IT Services, ETH Zurich, Zurich, 8092, Swizterland.
[4]NEXUS Personalized Health Technologies, ETH Zurich, Basel, 4058, Swizterland.

*Corresponding author(s). E-mail(s): niko.beerenwinkel@bsse.ethz.ch;
Contributing authors: lara.fuhrmann@bsse.ethz.ch; kim.jablonski@bsse.ethz.ch; ivan.topolsky@bsse.ethz.ch; aashilbatavia@gmail.com; nico.borgsmueller@bsse.ethz.ch; pelin.icer@bsse.ethz.ch; carrara@nexus.ethz.ch; chaoran.chen@bsse.ethz.ch; arthur.dondi@bsse.ethz.ch; monica.dragan@bsse.ethz.ch; david.dreifuss@bsse.ethz.ch; anika.john@bsse.ethz.ch; blanger@student.ethz.ch; michal.okoniewski@id.ethz.ch; louis.duplessis@bsse.ethz.ch; uwe.schmitt@id.ethz.ch; singer@nexus.ethz.ch; tanja.stadler@bsse.ethz.ch;
†These authors contributed equally to this work.

Abstract

The large amount and diversity of viral genomic datasets generated by next-generation sequencing technologies poses a set of challenges for computational data analysis workflows, including rigorous quality control, scaling to large sample sizes, and tailored steps for specific applications. Here, we present V-pipe 3.0, a computational pipeline designed for analyzing next-generation sequencing data of short viral genomes. It is developed to enable reproducible, scalable, adaptable, and transparent inference of genetic diversity of viral samples. By presenting two large-scale data analysis projects, we demonstrate the effectiveness of V-pipe 3.0 in supporting sustainable viral genomic data science.

Keywords: next-generation sequencing, NGS data processing, sustainable data analysis workflow, benchmark, global haplotype reconstruction, viral genetic diversity

1 Background

With the advent of next-generation sequencing (NGS) technologies, large amounts of viral genomic data are being generated, which can no longer be easily analyzed on personal computers [1]. As this availability of high-coverage data sets brings interesting research opportunities but also computational challenges, many new processing and analysis tools are being developed. In particular, new possibilities of characterizing viral variants and analyzing the genetic diversity of viral sequencing samples have emerged [2, 3]. While inter-host variability describes how viral strains differ between separate hosts, intra-host, or within-host, variability measures the diversity of viral strains within a single host. Within-host genetic diversity is especially relevant to understanding disease progression and treatment options [4, 5]. In addition to clinical or experimental samples, there has been an increasing abundance of environmental samples also showing high within-sample variability, such as wastewater samples. These samples can contain a diverse array of viruses, enabling the monitoring of pathogens on a larger scale, encompassing cities, regions, and countries [6, 7].

For estimation of within-sample diversity from NGS samples, several data processing steps and tools are needed. Due to the complexity of the data, these tools should

be executed as part of a processing workflow. Typically they comprise tools for quality control, sequence alignment, consensus sequence assembly, diversity estimation, and result visualization. Various workflows have been proposed which try to accomplish these goals including V-pipe [8], ViralFlow [9], nf-core/viralrecon [10] and HAPHPIPE [11]. The adaptability of these workflows becomes crucial as different types of viruses require tailored analysis approaches. This need became evident during the SARS-CoV-2 pandemic, emphasizing the rapid emergence of specific requirements vital to public health [12]. For example, samples originating from diverse sources, such as clinical or wastewater settings, require application-specific processing steps that need to be supported in the same workflow.

Another effect of the SARS-CoV-2 pandemic is that a substantial increase in sequencing capacities has led to unprecedentedly large numbers of samples becoming publicly available, e.g., on the European Nucleotide Archive (ENA; [13]) or GenBank [14]. Analysis workflows need to be able to handle such large amounts of data in order to be beneficial to public health and epidemiological advances. Hence, it is critical for workflows to not only include a broad range of functionalities, but also to enable and promote sustainable data processing practices to ensure their effectiveness and long-term success.

NGS data processing workflows offer a range of diversity estimation approaches at different spatial genomic scales: mutation calling, local and global haplotype. Mutation calling refers to detecting single base pair mutations or variations at specific positions within the genome. Global haplotypes refer to the reconstruction of complete haplotypes that span the entire length of the viral genome. Midway between single mutations and global haplotypes, local haplotypes focus on identifying genomic variants spanning short genomic regions that are entirely covered by sequencing reads. The reconstruction of global haplotypes is the most challenging task as multiple reads

need to be assembled together to cover a whole genome, but it provides the most comprehensive measure of viral diversity [15]. Local haplotypes not only estimate local viral diversity directly, but they also provide the mutation calls of highest accuracy by leveraging locally co-occurring mutations.

As the methodologies for viral diversity estimation and data sources can be heterogeneous, understanding the performance of each tool and benchmarking them in a realistic way is difficult. Additionally, different methods may excel in different scenarios. Therefore, continuous benchmarking of these methods is crucial to identify the most suitable one for any given data source and scenario. Consequently, it is important to provide data analysis procedures as publicly available workflows designed in a sustainable manner. This approach facilitates continuous re-evaluation of the benchmarking workflow with new and updated parameter settings. This is needed as new methods are being developed which have to be compared to already existing ones and new test data sets become available, either new synthetic data sets with new simulation setups, or real data sets with new experimental setups. Finally, completely new application domains can appear which require adapting the existing benchmarking workflow.

Here, we present V-pipe 3.0, a sustainable data analysis workflow for diversity estimation from viral NGS samples. Sustainability comprises reproducibility, scalability, adaptability, and transparency of the workflow [16]. V-pipe 3.0 builds upon the foundation of V-pipe [8], but has undergone significant extensions and refinements to increase functionality and to adhere to sustainable data processing standards [16]. The development of V-pipe 3.0 was primarily driven to address the new demands and challenges that became evident in the SARS-CoV-2 pandemic. We highlight how the workflow has been designed to achieve these properties and describe how they have been crucial for the application of V-pipe 3.0 to large-scale data analysis projects. In

particular, we present a new and efficient workflow that enables the processing of hundreds of thousands of samples. We demonstrate how automated source code testing makes it possible to quickly make new functionalities and bug fixes available to end users and how its modular design allows to quickly implement application-specific features. Depending on the user input, V-pipe 3.0 dynamically utilizes different tools for each processing step. In addition to the NGS data processing pipeline, we have incorporated a sub-workflow for benchmarking. This benchmarking module allows users to compare viral diversity estimation methods using synthetic and experimental data. The module itself is sustainably implemented and it enables adding new methods and test data sets. We demonstrate its use by conducting a benchmarking study where we apply a set of global haplotype reconstruction methods to both synthetic and real data sets. Lastly, we compare V-pipe 3.0 to workflows for similar applications, provide an overview of their functionalities, and compare their features in terms of sustainability.

2 Results

V-pipe 3.0 is a bioinformatics workflow which combines various tools for analyzing viral NGS data (Table 1). It is based on V-pipe, a pipeline designed for analyzing NGS data of short viral genomes [8] and extends it not only in terms of functionalities (Table 1) but also by consistently implementing principles of sustainable data analysis (Table 2). In the initial step of the pipeline, the raw sequencing reads in fastq format undergo a quality control process. Following this, the reads are aligned, and subsequently, the user-specified diversity estimation methods are executed (Figure 1). To ensure sustainable data analysis, we followed the hierarchy of sustainability proposed in [16] and created a reproducible, scalable, adaptable, and transparent workflow. It has been widely recognized that these aspects are crucial to scientific progress but often lacking in current literature [17, 18]. In the following, we will provide a detailed explanation of the reimplementation and extensions that were undertaken during the development

of V-pipe 3.0. To demonstrate that V-pipe 3.0 effectively addresses the challenges of sustainable data analysis we follow the four aspects in Mölder et al.'s hierarchy [16].

2.1 Reproducibility

Reproducibility allows other researchers to execute an existing workflow and obtain the exact same results as the original workflow authors. To achieve this goal, we define all software dependencies in Conda environments, which makes V-pipe 3.0 portable between different computing platforms. That way, V-pipe 3.0 can be executed without complicated, manual installation procedures. To ensure successful installation and reproducible execution on different systems, we have written GitHub Actions [19], workflow scripts [20] which automatically perform test installations of V-pipe 3.0 on Mac OS and Linux systems and run end-to-end tests by executing tutorials with real example data. For each update of V-pipe 3.0 these workflow scripts are automatically executed and report about installation problems or issues on the test data.

Additionally, V-pipe 3.0 enables reproducible benchmarking of viral diversity estimation methods using the newly integrated benchmarking module, a critical capability considering the constant evolution of new methods and the availability of new datasets. The benchmarking module is a Snakemake based workflow which automatically applies a set of selected tools to various synthetic and real data sets, computes their respective performances in terms of precision and recall, and summarizes the results. The benchmarking workflow is itself sustainably implemented. Adding new tools and data sets to this benchmark is very easy and only requires the addition of a single file and no further modifications of the workflow. As a concrete demonstration of the effectiveness of the benchmarking module, we conducted a benchmarking study focused on global haplotype reconstruction (Section 3.3 below).

2.2 Scalability

Scalable workflows can handle and process increasing amounts of data without compromising on performance or efficiency. To achieve scalability, we utilize efficient programming techniques to execute jobs on a computing cluster, ensuring optimal performance. For example, we dynamically specify cluster resources to adapt to the specific data requirements, facilitating smoother deployment on new cluster environments and enable the parallel execution of unrelated data analysis steps. Furthermore, we validate user configuration files using JSON Schema [21] during startup to identify potential runtime errors early. Lastly, we split centralized tasks among multiple compute nodes and perform per-sample distributed computation of summary statistics. In order to make large-scale analyses of public data sets easier, V-pipe 3.0 includes an input data retrieval functionality which requires a set of SRA accession numbers [13] as input and automatically downloads all data files needed to run the whole workflow. Further, scripts are available which facilitate the unattended mass-import of raw files as produced by Illumina's demultiplexing software into the structure that V-pipe 3.0 expects as input. To help with common post-processing steps, we have added scripts to facilitate SRA and GISAID database upload of compressed raw reads and of generated consensus sequences, including the summary quality reports assessing the plausibility of frameshift-causing insertions and deletions. With these features, V-pipe 3.0 has been shown to handle more than 100,000 samples efficiently [22–25].

2.3 Adaptability

Adaptability refers to making it easy for other researchers to build upon an existing workflow and extend it for their application- and domain-specific needs. To demonstrate the ease with which new software components and scrips can be introduced we added two methods for viral diversity estimation: first, PredictHaplo [26] a well-performing global haplotype reconstruction method, and second, a script for the

computation of within-sample diversity indices [27], including Shannon Entropy and population nucleotide diversity. The indices are often applied to compare diversity between samples and have been used for the estimation of time since infection [28]. The addition of new methods requires only the definition of a Conda environment with the required software dependencies and the definition of a Snakemake rule executing the method or script. Additionally, we run automated integration and unit tests using GitHub Actions workflows [19, 20] on every commit submitted to the repository. This verifies the reliability, consistency and correctness of the overall workflow. We use data sets from different viruses in our tests to make sure that V-pipe 3.0 and the newly added features are running successfully from start to end.

Further, V-pipe 3.0 can be easily optimized for different viruses through its configuration setup. The base configuration is virus-agnostic while virus-specific settings (specific reference sequences, different alignment tools, etc.) can be easily plugged in. This allows a quick adaptation of V-pipe 3.0 to any virus, without requiring complex workflow changes. For example, we offer specific configuration setups for HIV, SARS-CoV-2, Poliovirus, Herpes simplex virus, Influenza A virus, Respiratory syncytial virus B, and Drosophila C virus. These setups include selection of suitable reference files, read alignment software, and post-processing steps for each target virus. We demonstrate how to write such configuration files through the example of monkeypox in Figure 2. The configuration defines which alignment and diversity estimation method should be applied, which reference should be used, and which outputs and processing steps should be run. Further, for each method, users can specify the parameter choices.

2.4 Transparency

Transparency refers to the ability to easily comprehend a given workflow. This is particularly crucial for ensuring interpretability and facilitating efficient collaboration in

large-scale projects with many stakeholders. The documentation of V-pipe 3.0 is written in the form of dynamic scripts which allows testing of the configuration options in an automated fashion and making sure they always represent the latest release version and do not contain outdated information. Additionally, V-pipe 3.0 offers a range of tutorials that cover various applications, including the processing of SARS-CoV-2 or HIV samples, as well as a tutorial specifically designed for processing wastewater samples [29].

In order to facilitate prompt user access to new functionalities and accelerate the onboarding process for new users, we provide four deployment options: (1) a Bash script which automatically creates the required Conda environments, installs all dependencies, and initializes a project structure, (2) Snakemake's snakedeploy tool to install V-pipe 3.0 in the standardized Snakemake fashion, (3) a Docker container [30] which is automatically generated for every new release and for the master branch of the GitHub repository, and (4) execution within a workflow execution service, such as Sapporo [31], by fetching V-pipe from a tools repository service, such as WorkflowHub [32]. Further, the configuration definition summarizes the steps of the workflow in one single file and hence also facilitates information sharing between collaborators.

3 Applications

The development of V-pipe 3.0 was primarily driven by two large-scale SARS-CoV-2 surveillance projects. Continuous updates and extensions of the pipeline have been motivated by the evolving demands of the pandemic. With each workflow update, the entire sample cohort underwent reanalysis using the latest pipeline version. This approach guaranteed consistent results that align with the most recent advancements in the pipeline. In the following, we present how sustainable data processing using V-pipe 3.0 was key to the successful execution of the two surveillance projects,

and we demonstrate the benchmarking module by conducting a global haplotype reconstruction benchmarking study.

3.1 Swiss SARS-CoV-2 Sequencing Consortium

In the scope of the Swiss SARS-CoV-2 Sequencing Consortium [33], V-pipe 3.0 was consistently utilized to process sequencing data and generate consensus sequences. This continuous usage began with the first consortium sequencing run on 23 April 2020, and concluded when the consortium was dissolved in January 2023. V-pipe 3.0 demonstrated its adaptability by transitioning from its original focus on HIV to processing samples from SARS-CoV-2. The first Swiss SARS-CoV-2 case was reported on 25 February 2020 [34], and we submitted the first sequence processed by V-pipe 3.0 to GISAID on 25 May 2020 (accession number: EPI_ISL_451681, sampled on 12 March 2020). The fast development and changing demands in the SARS-CoV-2 pandemic required the rapid development of new tools that had to be integrated in the processing pipeline, for example, the frameshift insertion/deletion checks mentioned before. Apart from adaptability, portability and reproducibility were essential for this project, as it involved analyses conducted by different individuals from various academic groups on their own computing facilities. Since the consensus sequences and their Pango lineage [35] designations were reported to the Swiss Federal Office of Public Health to inform public health decision-making, reproducibility was essential to guarantee reliable, consistent, and trustworthy results. Further, scalability to maximize the use of computational resources made it possible to handle the large amounts of clinical SARS-CoV-2 samples throughout the pandemic [36], which resulted in 74,409 consensus sequences being submitted to GISAID [37] as of 21 Sep 2023 (accessed 21 Sep 2023). At the peak of our efforts, V-pipe 3.0 processed up to 1500 clinical samples on a weekly basis (Figure 3A), providing a substantial part to the national surveillance efforts of circulating SARS-CoV-2 variants in Switzerland [22–24].

3.2 Swiss surveillance of SARS-CoV-2 genomic variants in wastewater

Another successful application of V-pipe 3.0 has been the Swiss surveillance of SARS-CoV-2 genomic variants in wastewater [38] (Figure 3C). Wastewater samples contain mixtures of multiple SARS-CoV-2 lineages, and hence workflows targeting diversity analysis are prime candidates for handling them. V-pipe 3.0 was used to analyze the sequencing data and to estimate the abundances of the circulating SARS-CoV-2 variants in Switzerland. In particular, the wastewater analysis enabled the early detection of new variants of concern such as Alpha (B.1.1.7) [6]. Starting in December 2020, V-pipe 3.0 has been continuously used to process wastewater samples from 6-10 different locations 3-7 times per week [38] (Figure 3B). Since then, V-pipe 3.0 has been the core of the automated monitoring of the circulating SARS-CoV-2 genomic variants in Switzerland (Figure 3C). As of 23 May 2024, 7785 samples have already been submitted to the ENA project (PRJEB44932).

The complexity of the SARS-CoV-2 variant mixtures in wastewater samples required additions to the standard workflow, namely primer trimming and the newly developed methods COJAC [6] and LolliPop [39] for variant detection and time-series deconvolution of the variant mixtures, respectively. The modular and standard Snakemake structure of V-pipe 3.0 facilitated the integration of the new functionalities through adding new Snakemake rules for their execution. Lastly, the involvement of the large number of stakeholders and collaborators in the surveillance consortium of SARS-CoV-2 genomic variants in wastewater required transparency of the whole analysis pipeline. All stakeholders and developers had to be aware of the functionalities and steps of the data processing. This was possible through the modular structure and the clear configuration files used by V-pipe 3.0, as well as the fact that all parts of the pipeline are open source and their configuration automatically documented.

3.3 Global haplotype reconstruction benchmark

To showcase the strengths of V-pipe 3.0's benchmarking module, we designed a global haplotype reconstruction benchmarking study. Global haplotype reconstruction is a useful methodology in genetic research as it allows for a comprehensive understanding of the underlying genetic variations within a population. Due to the computational challenges involved in global haplotype reconstruction [40], it serves as a valuable application for the benchmarking module. Additionally, this benchmarking study provides an opportunity to evaluate new methods that could potentially be included in V-pipe 3.0. In our study, we compared the performance of the probabilistic method PredictHaplo and the graph-based methods CliqueSNV, HaploConduct, and HaploClique. We setup the benchmarking such that the methods were tested on two synthetic data sets and on one real data set.

Using the integrated synthetic data generation component of the module, we consider a genome of length $10,000$ bp, generate a population of 10 haplotypes (Population 1) and simulate Illumina reads of length 200bp (Section 7.2). We vary the coverage between $500, 1000, 5000, 10,000$ in order to investigate how well the methods are able to recover low-frequency haplotypes as the total coverage decreases.

We observe that PredictHaplo achieves perfect precision of 1 in all cases, CliqueSNV's mean precision is between 0.60 and 0.68 with a slight increase with higher coverage (Figure 4A). In terms of recall, CliqueSNV features the highest recall of $0.5 - 0.6$ which remains constant over all coverage values, while PredictHaplo's recall increases up to 0.30 for the highest coverage of $10,000$. Consequently, the recall performance of CliqueSNV is less dependent on the coverage level when compared to PredictHaplo. Across all coverage values, CliqueSNV and PredictHaplo consistently achieve N50 scores of $10,000$, covering the entire genome length. In contrast, both HaploClique and HaploConduct fail to cover even a quarter of the genome, and show a precision and recall of 0 in all cases. This indicates that all sequences predicted by

HaploClique and HaploConduct have relative edit distance greater than 0.01 to any true haplotype, and no true haplotypes are recovered. The poor performance (according to this measure) could be attributed to HaploClique being executed with restricted clique size and maximal clique size parameters, which may not be adequate for the assembly of longer regions. This parameter choice was necessary to prevent excessively long runtime and memory consumption. For all methods, we see a general trend of growing runtime with increasing coverage. CliqueSNV consistently requires the least amount of time to run, while PredictHaplo needs over an hour for the highest coverage (Figure 4A).

By varying the haplotype population in terms of number of haplotypes and pairwise distance while keeping the coverage constant, we generate five additional haplotype populations (populations 2-6, Figure 5C). Across all populations, we again observe perfect precision of 1 for PredictHaplo. For populations 3 and 4, CliqueSNV has nearly perfect precision of $0.83 - 1$. However, CliqueSNV is only able to detect haplotypes from the larger group of 20 haplotypes. Both CliqueSNV and PredictHaplo obtain their highest recall for populations 1 and 2 (Figure 4B), which are the two populations with only 10 haplotypes, and their lowest recall for populations 5 and 6 each with 55 haplotypes. This indicates that both tools are not able to appropriately deal with large haplotype populations. As before, CliqueSNV's generally higher recall than PredictHaplo's, is due to CliqueSNV predicting a larger amount of haplotypes than PredictHaplo. In all simulated populations, we observe that PredictHaplo predicts a single haplotype per cluster while CliqueSNV finds, if any, always multiple ones per cluster (Figure 4B). HaploClique and HaploConduct remain at a recall and precision of 0.

Next, we used the experimental HIV-5-strain mixture [15] to evaluated the methods on a real sequencing data. We observe that precision and recall remain in the range of $0.2 - 0.4$ for PredictHaplo. CliqueSNV and HaploConduct remain at 0 for precision

and recall. As before, PredictHaplo's and CliqueSNV's reconstructions cover nearly the whole genome while HaploConduct covers less than a fifth (Figure 4C).

In summary, our benchmarking studies demonstrates that CliqueSNV exhibits the shortest runtime and delivers the highest recall performance for the simulated samples, whereas PredictHaplo exhibits superior precision for the same samples. This can mostly be explained by CliqueSNV typically recovering a larger amount of haplotypes than PredictHaplo. PredictHaplo was better able to reconstruct global haplotypes with the real data set both in terms of precision and recall. Overall, the results of our benchmark study indicate that the performance of all methods is diverse and highlights the need of continuous benchmarking as new methods are developed.

The benchmarking study can be effortlessly reproduced due to its adherence to Snakemake guidelines. It can be easily customized for different scenarios by integrating a novel data generation script. Moreover, incorporating new methods into the study merely requires adding a short script to execute those methods. Thus, our benchmarking study itself aligns with sustainable data processing practices.

4 Comparison to other workflows

We compare V-pipe 3.0 to other relevant viral bioinformatics pipelines for within-sample diversity estimation, focusing on functionalities and sustainability (Table 2). The compared pipelines include nf-core/viralrecon [10], HAPHPIPE [11], ViralFlow [9] and the pipeline of the GalaxyProject SARS-CoV-2 analysis effort [41–43]. These pipelines are all open source, actively maintained, and provide within-sample diversity estimates for Illumina sequencing reads. Active maintenance is crucial in this rapidly evolving field as even frequently used methods are still in continuous development and contain bugs for corner cases that only become evident with the rise of massive data sets in recent years.

During the SARS-CoV-2 pandemic many processing pipelines have been developed, however the vast majority of those are specific to SARS-CoV-2, tailored to the ARTIC protocol [44] combined with Illumina sequencing, and only aim to produce consensus sequences. Since SARS-CoV-2 has limited genetic diversity and a well-known reference sequence, these pipelines cannot be easily adapted for the general case.

ViralFlow and the pipeline of the GalaxyProject SARS-CoV-2 analysis effort, however, also provides variant calling and downstream analysis for SARS-CoV-2 lineage assignment. In terms of functionality, all data processing pipelines enable *de novo* assembly, except for ViralFlow and the pipeline of the GalaxyProject SARS-CoV-2 analysis effort. HAPHPIPE and nf-core/viralrecon use SPAdes [45] for this purpose, while V-pipe 3.0 utilizes Vicuna [46]. For read alignment, consensus sequence generation, and single nucleotide variant calling, each pipeline offers different combinations of tools and methods. For instance, both ViralFlow and nf-core/viralrecon provide the option to use iVar's variant calling and consensus sequence generation [47]. HAPHPIPE uses GATK for variant calling [48], the pipeline of the GalaxyProject SARS-CoV-2 analysis effort uses LoFreq [49] for variant calling for Illumina and Medaka [50] for Nanopore sequencing data, and V-pipe 3.0 integrates three mutation callers: LoFreq [49], VILOCA [51] and ShoRAH [52], where ShoRAH and VILOCA also provides local haplotypes. V-Pipe 3.0 stands out with its integrated benchmarking module (Table 2, Section 7.1). This framework allows for simulation of sequencing reads from flexible haplotype populations and performance evaluation of various methods. In contrast, [53] presented a benchmarking workflow for a global haplotype caller that is not easily adaptable due to hard-coded simulation parameters in bash-scripts.

Apart from its functionalities, sustainability is an essential factor for data analysis of enduring impact. V-pipe 3.0, ViralFlow, nf-core/viralrecon and the GalaxyProject SARS-CoV-2 analysis effort ensure reproducibility and portability by providing software dependency definitions, automatically installing all necessary dependencies

upon pipeline installation or execution (Table 2). HAPHPIPE, on the other hand, requires manual installation of some software dependencies. In addition, V-pipe 3.0, ViralFlow, and nf-core/viralrecon offer container services like Docker, ensuring full pipeline portability and reproducibility (Table 2). All five pipelines are transparent and open source, utilizing publicly available tools and methods. They provide documentation for installation and execution. In addition, HAPHPIPE and V-pipe 3.0 offer tutorials and examples to aid users in applying the pipelines to their data. Both nf-core/viralrecon and V-pipe 3.0 have code structures that conform to recommended standards for Nextflow and Snakemake workflows, ensuring code readability for external users, which makes adding new features straightforward. ViralFlow and HAPHPIPE follow more custom code structures, making it challenging to add new features or modify the workflow, thus limiting their adaptability.

Overall, with their portability, automatic tests and gold standard code structure, the workflows V-pipe 3.0, nf-core/viralrecon, and the pipeline of the GalaxyProject SARS-CoV-2 analysis effort can provide sustainable data processing and analysis. While V-pipe 3.0 not only provides additional options for downstream analysis like analysis of co-occurrence of mutations on amplicons (COJAC), or kernel-based deconvolution of time-series mutation frequencies into variants (LolliPop), it also integrates the largest selection of tools for each processing step to ensure suitable processing for different samples. For example, for alignment V-pipe 3.0 supports BWA MEM [54], Bowtie 2 [55], ngshmmalgin [8] and minimap2 [56] which allows the processing of samples with very high diversity regions.

5 Discussion

We have presented V-pipe 3.0, a sustainable data analysis pipeline designed for analyzing next-generation sequencing data of viral genomes and inferring the genomic diversity of intra-host or environmental samples. V-pipe 3.0 has been designed to

be reproducible by following Snakemake's best-practice guidelines, adaptable by implementing virus-specific configuration files which can be quickly exchanged, and transparent by providing automatically tested usage examples, which are available online. We have demonstrated the effectiveness and utility of these developments by highlighting its application to two large-scale projects, where V-pipe 3.0 was used in a production setting to process thousands of samples over multiple years.

A core functionality of V-pipe 3.0 is the estimation of viral diversity from NGS data. A multitude of viral diversity estimation tools exist, making it challenging for users to determine the appropriate tool for their samples. Additionally, the choice of method depends on the desired downstream analysis of the results. To address this challenge, we have developed a versatile benchmarking module that facilitates the continuous assessment of the performance and limitations of existing diversity estimation methods. As this field is still quickly advancing, continuous benchmarking of new and established methods is needed. For this purpose, we focus on making the addition of new tools and test data sets to the workflow as straightforward as possible. Adding new methods is as easy as writing a single script which defines how to execute the tool and how to install it. New data sources can be either synthetic or derived from real experimental samples. In the synthetic case, different haplotype evolution modeling assumptions can be specified in a flexible way. Real data sources can be automatically downloaded and pre-processed as part of the workflow.

Given the mixed performance observed in our benchmark study for global haplotype reconstruction, it is evident that the current methods may not satisfy the demands of downstream applications. The issues with performance can be attributed not only to the limitations of inference methods but also to the complex population structures inherent to viruses. Consequently, the practical application of global haplotype reconstruction is heavily constrained by these poor performing and often non-scalable

methods, and would require improved scalable methods that explicitly account for the uncertainty of the results.

When comparing V-pipe 3.0 to other pipelines with similar purposes we found that, apart from V-pipe 3.0, only nf-core/viralrecon provides sustainable data processing taking into account reproducibility, portability, adaptability and transparency by following Nextflow's best-practice guidelines. V-pipe 3.0 sets itself apart from the other pipelines by offering a broader range of integrated tools and functionalities, supported by thorough documentation and tutorials that address various application settings.

6 Conclusions

In summary, we have developed V-pipe 3.0 a sustainable data analysis pipeline for within-sample diversity estimation that can be easily applied to large numbers of samples by other researchers while keeping its execution robust and its workflow structure open to modifications. We have created a benchmarking module for one of V-pipe 3.0's core functionalities which can be continuously updated when new methods and data sets appear. By continuing our close interactions and exchange with users through our mailing list, active GitHub discussions and workshops, we will further expand V-pipe 3.0 to support different kinds of sequencing data, make it more robust to unpredictable failure points in cluster environments and further improve interoperability with data providers and users.

7 Methods

In the following, we introduce V-pipe 3.0's benchmarking module and its application to the global haplotype reconstruction benchmarking study in detail.

7.1 Benchmarking module

V-pipe 3.0's benchmarking module allows the benchmarking of global haplotype reconstruction methods on real and simulated data. For simulated data the workflow consists of four steps: generation of haplotype populations, shotgun read simulation, methods execution and performance evaluation (Figure 5A). In the case of real data, the first two steps are replaced by a data downloading and alignment step.

Generation of synthetic data sets

The synthetic data sets are generated in two steps. First, viral haplotype populations are generated. In the second steps, reads are simulated (Figure 5A). If no reference sequence is provided by the user, it is generated by drawing bases uniformly at random for each position based on the user-provided genome length.

We integrated two options for the viral haplotype population generation based on user-specified mutation rates or pairwise distances. Incorporating new methods involves the addition of a new script to the module, which generates haplotypes in fasta format as output. In the case of haplotype generation based on mutation rates, substitutions, deletions and insertions are randomly introduced into the master sequence based on the user-specified rates μ. The frequency composition of those haplotypes in the population is derived from haplotype frequencies $f = (f_1, ..., f_K)$ provided by the user. These simulation settings allow testing the reconstruction limits of the different viral diversity estimation methods.

In the case of haplotype generation by pairwise distances, we simulate hierarchical relationships among the haplotypes by generating two groups of closely related haplotypes that share a common ancestor (Figure 5B). First, using the user-specified between-group pairwise distance d_{12} two haplotypes are generated from the reference sequence. Second, for each haplotype, child-haplotypes are generated by introducing

mutations based on the respective within-group pairwise distance (d_1 and d_2 respectively) and group size (n_1 and n_2 respectively). The frequency distribution of the generated haplotypes is obtained from a geometric series with a given ratio (default: 0.75), this results in a few high-frequency and many low-frequency haplotypes being present. Additionally the frequency distribution can also be drawn from a Dirichlet distribution with user-povided concentration parameters α_i.

Given a user-specified per-position coverage and read length, paired-end reads are simulated in shotgun-mode using the ART Illumina read simulator ([57], RRID: SCR_006538).

Integration of real data sets

In addition to synthetic data sets where the ground truth is known, real data sets are included in the benchmark. We test the global haplotype reconstruction methods on sequencing reads from the 5-virus-mix presented in [15] (SRA accession number: SRX342666). It provides Illumina MiSeq reads for a mixture of five HIV-1 strains: HXB2, 89.6, JR-CSF, NL4-3 and YU-2 and thus gives an estimate of the ground truth which can be used for performance evaluation. The benchmark workflow is designed to make the addition of further real data sets easily possible.

Performance evaluation

To evaluate the performance of each method in the global haplotype reconstruction benchmark, we compute precision and recall for the recovery of ground truth global haplotypes for each method in each condition. To do so, we consider the ground truth set of haplotype sequences and the set of sequences produced by a method. For each predicted sequence, we check if there exists a ground truth sequence with a relative edit

distance below a predefined threshold γ. We define the relative edit distance ED_{rel} as

$$ED_{rel} = \frac{ED}{\max(L_{pred}, L_{true})} \tag{1}$$

where ED is the edit distance between a predicted and ground truth haplotype which have lengths L_{pred} and L_{true} respectively. If $ED_{rel} < \gamma$, the predicted haplotype counts as a true positive, otherwise as a false positive. To compute the number of false negatives, we iterate over all ground truth sequences. We count a false negative if a ground truth sequence has no matching, i.e., relative edit distance below a certain threshold, predicted sequence. From this, we compute precision as $TP/(TP + FP)$ and recall as $TP/(TP + FN)$. We use $\gamma = 0.01$ as the relative edit distance threshold in the benchmark study.

Two-dimensional embeddings of haplotype sequences are generated by applying multidimensional scaling with precomputed edit distances between all sequences [58].

We use MetaQUAST to compute measures of assembly quality for the reconstructed haplotypes [59]. In particular, we compute the N50 score which, in this context, equals the length of the shortest haplotype, which together with all larger haplotypes, covers at least half the genome.

7.2 Global haplotype reconstruction benchmark study

We used the benchmarking module to benchmark global haplotype reconstruction methods. The scripts to reproduce the benchmarking study are available on GitHub [60].

Datasets

We generated two synthetic data sets applying the distance-based haplotype generation mode and used one real data set. In the first synthetic data set, we considered a genome of length 10000 with reads of length 200. We then generated two groups of

haplotypes such that group one has size $n_1 = 5$ and group two has size $n_2 = 5$, the average pairwise sequence distance within group one is $d_1 = 50$, the average pairwise sequence distance within group two is $d_2 = 20$, and the average pairwise sequence distance between the two groups is $d_{12} = 200$. We varied the coverage between $500, 1000, 5000, 10000$ in order to investigate how well the methods are able to recover low-frequency haplotypes as the coverage decreases. In the second synthetic data set, we considered a genome of length 10000 with reads of length 200 at a constant coverage of 1000. We then used the six haplotype population parameter settings as specified in Figure 5C in order to investigate how well the methods are able to recover different types of haplotype populations with different diversity levels. For the real data set, we used the 5-virus-mix which contains the HIV-1 strains HXB2, 89.6, JR-CSF, NL4-3 and YU-2 mixing in uniform proportions.

Global haplotype methods

We considered all methods discussed in [40] for which a Conda package is available. They are aBayesQR [61], CliqueSNV [62], HaploClique [63], HaploConduct [64], PEHaplo [65], PredictHaplo [26], QuasiRecomb [66], and RegressHaplo [67]. From the benchmark study we excluded aBayesQR because the program failed to parse the input sequencing reads, PEHaplo because it failed execution during the result assembly, QuasiRecomb as it terminated during startup and Regresshaplo, because not all dependencies of its Conda package were available. The remaining tools are HaploConduct, HaploClique, PredictHaplo and CliqueSNV which are all reference-based global haplotype reconstruction methods. This means that they rely on the existence of a viral reference sequence which is similar to the haplotypes expected to occur. The input reads are then typically mapped against this reference sequence which makes reconstructing global haplotypes easier, because read positions relative to the genome

are available, but also introduces a bias, as haplotypes which are dissimilar to the reference might not be captured. For the real data set, we had to exclude HaploClique for its excessive memory consumption.

Availability of Source Code and Requirements

- Project name: V-pipe
- Project home page: https://github.com/cbg-ethz/V-pipe
- Operating system(s): Platform independent
- Programming language: Python, Shell, CSS, Dockerfile, Jupyter Notebook
- License: Apache-2.0 license
- RRID: SCR_025399
- Biotools: biotools:v-pipe
- WorkflowHub DOI: 10.48546/WORKFLOWHUB.WORKFLOW.301.5

Data Availability

All supporting data and materials are available in the Software Heritage [68]. This includes details on how to reproduce the synthetic benchmark datasets and all the scripts to run the workflow. The sequencing data of the 5-virus-mix used in the global haplotype reconstruction is accessible on the Sequence Read Archive under the accession number SRX342666. The SARS-CoV-2 consensus sequences generated are available on GISAID with GISAID Identifier: EPI_SET_231013cd. The samples from the Swiss SARS-CoV-2 Wastewater Surveillance have been submitted to the ENA Project under the project id: PRJEB44932.

Competing interests

The authors declare that they have no competing interests.

Funding

LF was funded by European Union's Horizon 2020 research and innovation program, under the Marie Skłodowska-Curie Actions Innovative Training Networks grant agreement no. 955974 (VIROINF).

Authors' contributions

LF, KPJ, IT and NB worked on the conceptualization and design of the pipeline. IT, KJP, LF, AAB, NBorg, PIB, MC, CC, AD, MD, DD, AJ, BL, MO and US were involved in implementing or adding new methods or tools. KJP conducted the benchmark study. CC, DD, IT, LdP, TS, MC, FS, NB, LF, and KPJ were involved in the analysis and processing of the SARS-CoV-2 clinical and wastewater samples. DD, IT, NB, KJP, LF were involved in the visualization of the results. KPJ and LF were writing the original draft. NB, LdP, TS, FS were involved in reviewing and editing of the manuscript. All authors read and approved the final manuscript.

Acknowledgements

We gratefully acknowledge all data contributors, i.e., the authors and their originating laboratories responsible for obtaining the specimens, and their submitting laboratories for generating the genetic sequence and metadata and sharing via the GISAID Initiative [69].

References

[1] Pereira R, Oliveira J, Sousa M. Bioinformatics and computational tools for next-generation sequencing analysis in clinical genetics. Journal of clinical medicine. 2020;9(1):132.

[2] Barzon L, Lavezzo E, Costanzi G, Franchin E, Toppo S, Palù G. Next-generation sequencing technologies in diagnostic virology. Journal of Clinical Virology. 2013;58(2):346–350.

[3] Capobianchi M, Giombini E, Rozera G. Next-generation sequencing technology in clinical virology. Clinical Microbiology and Infection. 2013;19(1):15–22.

[4] Ko HY, Li YT, Chao DY, Chang YC, Li ZRT, Wang M, et al. Inter-and intra-host sequence diversity reveal the emergence of viral variants during an overwintering epidemic caused by dengue virus serotype 2 in southern Taiwan. PLoS neglected tropical diseases. 2018;12(10):e0006827.

[5] Bonnaud EM, Troupin C, Dacheux L, Holmes EC, Monchatre-Leroy E, Tanguy M, et al. Comparison of intra-and inter-host genetic diversity in rabies virus during experimental cross-species transmission. PLoS pathogens. 2019;15(6):e1007799.

[6] Jahn K, Dreifuss D, Topolsky I, Kull A, Ganesanandamoorthy P, Fernandez-Cassi X, et al. Early detection and surveillance of SARS-CoV-2 genomic variants in wastewater using COJAC. Nature Microbiology. 2022;7(8):1151–1160.

[7] Hillary LS, Maher KH, Lucaci A, Thorpe J, Distaso MA, Gaze WH, et al. Monitoring SARS-CoV-2 in municipal wastewater to evaluate the success of lockdown measures for controlling COVID-19 in the UK. Water Research. 2021;200:117214.

[8] Posada-Céspedes S, Seifert D, Topolsky I, Jablonski KP, Metzner KJ, Beerenwinkel N. V-pipe: a computational pipeline for assessing viral genetic diversity from high-throughput data. Bioinformatics. 2021;37(12):1673–1680.

[9] Dezordi FZ, Neto AMdS, Campos TdL, Jeronimo PMC, Aksenen CF, Almeida SP, et al. ViralFlow: a versatile automated workflow for SARS-CoV-2 genome

assembly, lineage assignment, mutations and intrahost variant detection. Viruses. 2022;14(2):217.

[10] Patel H, Varona S, Monzón S, Espinosa-Carrasco J, Heuer ML, nf-core bot, et al.: nf-core/viralrecon: nf-core/viralrecon v2.5 - Manganese Monkey. Zenodo. Available from: https://doi.org/10.5281/zenodo.6827984.

[11] Bendall ML, Gibson KM, Steiner MC, Rentia U, Pérez-Losada M, Crandall KA. HAPHPIPE: haplotype reconstruction and Phylodynamics for deep sequencing of Intrahost viral populations. Molecular biology and evolution. 2021;38(4):1677–1690.

[12] Knyazev S, Chhugani K, Sarwal V, Ayyala R, Singh H, Karthikeyan S, et al. Unlocking capacities of genomics for the COVID-19 response and future pandemics. Nature Methods. 2022;19(4):374–380.

[13] Leinonen R, Akhtar R, Birney E, Bower L, Cerdeno-Tárraga A, Cheng Y, et al. The European nucleotide archive. Nucleic acids research. 2010;39(suppl_1):D28–D31.

[14] Benson DA, Cavanaugh M, Clark K, Karsch-Mizrachi I, Lipman DJ, Ostell J, et al. GenBank. Nucleic acids research. 2012;41(D1):D36–D42.

[15] Giallonardo FD, Töpfer A, Rey M, Prabhakaran S, Duport Y, Leemann C, et al. Full-length haplotype reconstruction to infer the structure of heterogeneous virus populations. Nucleic acids research. 2014;42(14):e115–e115.

[16] Mölder F, Jablonski KP, Letcher B, Hall MB, Tomkins-Tinch CH, Sochat V, et al. Sustainable data analysis with Snakemake. F1000Research. 2021;10.

[17] Baker M. 1,500 scientists lift the lid on reproducibility. Nature. 2016;533(7604).

[18] Sayre F, Riegelman A. The reproducibility crisis and academic libraries. College & Research Libraries. 2018;79(1):2.

[19] GitHub Inc.: GitHub Actions Website. Accessed 2023-10-02. Available from: https://github.com/features/actions.

[20] V-pipe version 3.: GitHub Actions workflow scripts for installations and end-to-end tests. Accessed 2024-04-29. Available from: https://github.com/cbg-ethz/V-pipe/tree/master/.github/workflows.

[21] JSON Schema.: Website. Accessed 2023-10-03. Available from: https://json-schema.org/.

[22] Nadeau SA, Vaughan TG, Beckmann C, Topolsky I, Chen C, Hodcroft E, et al. Swiss public health measures associated with reduced SARS-CoV-2 transmission using genome data. medRxiv. 2021;.

[23] Chen C, Nadeau SA, Topolsky I, Manceau M, Huisman JS, Jablonski KP, et al. Quantification of the spread of SARS-CoV-2 variant B. 1.1. 7 in Switzerland. Epidemics. 2021;37:100480.

[24] Chen C, Nadeau SA, Topolsky I, Beerenwinkel N, Stadler T. Advancing genomic epidemiology by addressing the bioinformatics bottleneck: Challenges, design principles, and a Swiss example. Epidemics. 2022;39:100576.

[25] Kuipers J, Batavia AA, Jablonski KP, Bayer F, Borgsmüller N, Dondi A, et al. Within-patient genetic diversity of SARS-CoV-2. BioRxiv. 2020;.

[26] Prabhakaran S, Rey M, Zagordi O, Beerenwinkel N, Roth V. HIV haplotype inference using a propagating dirichlet process mixture model. IEEE/ACM transactions on computational biology and bioinformatics. 2013;11(1):182–191.

[27] Fuhrmann L, Jablonski KP, Beerenwinkel N. Quantitative measures of within-host viral genetic diversity. Current opinion in virology. 2021;49:157–163.

[28] Puller V, Neher R, Albert J. Estimating time of HIV-1 infection from next-generation sequence diversity. PLOS Computational Biology. 2017;13(10):e1005775.

[29] V-pipe version 3.: GitHub directory with tutorials to run V-pipe 3.0. Accessed 2024-04-29. Available from: https://github.com/cbg-ethz/V-pipe/tree/master/docs.

[30] Merkel D, et al. Docker: lightweight linux containers for consistent development and deployment. Linux j. 2014;239(2):2.

[31] Sapporo.: GitHub. Accessed 2023-10-03. Available from: https://github.com/sapporo-wes/sapporo.

[32] WorkflowHub.: Website. Accessed 2023-10-03. Available from: https://workflowhub.eu/.

[33] Swiss SARS-CoV-2 Sequencing Consortium.: Website. Accessed 2022-07-22. Available from: https://bsse.ethz.ch/cevo/research/sars-cov-2/swiss-sars-cov-2-sequencing-consortium.html.

[34] Swiss Federal Office of Public Health.: Press releases, 2020-02-25. Accessed 2023-01-18. Available from: https://www.admin.ch/gov/en/start/documentation/media-releases.msg-id-78233.html.

[35] Rambaut A, Holmes EC, O'Toole Á, Hill V, McCrone JT, Ruis C, et al. A dynamic nomenclature proposal for SARS-CoV-2 lineages to assist genomic epidemiology. Nature microbiology. 2020;5(11):1403–1407.

[36] Chen C, Nadeau S, Yared M, Voinov P, Xie N, Roemer C, et al. CoV-Spectrum: analysis of globally shared SARS-CoV-2 data to identify and characterize new variants. Bioinformatics. 2022;38(6):1735–1737.

[37] Khare S, Gurry C, Freitas L. B Schultz. M, Bach, G, Diallo, A, Akite, N, Ho, J, Tc Lee, R, Yeo, W, Core Curation Team, G, and Maurer-Stroh, S. 2021;p. 1049–1051.

[38] Beerenwinkel N.: Swiss Surveillance of SARS-CoV-2 genomic variants in wastewater. Accessed 2023-01-18. Available from: https://bsse.ethz.ch/cbg/research/computational-virology/sarscov2-variants-wastewater-surveillance.html.

[39] Dreifuss D, Topolsky I, Icer Baykal P, Beerenwinkel N. Tracking SARS-CoV-2 genomic variants in wastewater sequencing data with LolliPop. medRxiv. 2022;p. 2022–11.

[40] Jablonski KP, Beerenwinkel N. Computational Methods for Viral Quasispecies Assembly. In: Virus Bioinformatics. Chapman and Hall/CRC; 2021. p. 51–64.

[41] The Galaxy platform for accessible, reproducible and collaborative biomedical analyses: 2022 update. Nucleic Acids Research. 2022;50(W1):W345–W351.

[42] Baker D, Van Den Beek M, Blankenberg D, Bouvier D, Chilton J, Coraor N, et al. No more business as usual: Agile and effective responses to emerging pathogen threats require open data and open analytics. PLoS pathogens. 2020;16(8):e1008643.

[43] Galaxy.: GalaxyProject SARS-CoV-2 analysis effort workflows. Accessed 2024-04-30. Available from: https://galaxyproject.org/projects/covid19/workflows/.

[44] ARTIC protocol.: Website. Accessed 2023-10-03. Available from: https://artic.network/ncov-2019.

[45] Prjibelski A, Antipov D, Meleshko D, Lapidus A, Korobeynikov A. Using SPAdes de novo assembler. Current protocols in bioinformatics. 2020;70(1):e102.

[46] Yang X, Charlebois P, Gnerre S, Coole MG, Lennon NJ, Levin JZ, et al. De novo assembly of highly diverse viral populations. BMC genomics. 2012;13:1–13.

[47] Grubaugh ND, Gangavarapu K, Quick J, Matteson NL, De Jesus JG, Main BJ, et al. An amplicon-based sequencing framework for accurately measuring intrahost virus diversity using PrimalSeq and iVar. Genome biology. 2019;20(1):1–19.

[48] DePristo MA, Banks E, Poplin R, Garimella KV, Maguire JR, Hartl C, et al. A framework for variation discovery and genotyping using next-generation DNA sequencing data. Nature genetics. 2011;43(5):491–498.

[49] Wilm A, Aw PPK, Bertrand D, Yeo GHT, Ong SH, Wong CH, et al. LoFreq: a sequence-quality aware, ultra-sensitive variant caller for uncovering cell-population heterogeneity from high-throughput sequencing datasets. Nucleic acids research. 2012;40(22):11189–11201.

[50] ONT Research.: medaka: Sequence correction provided by ONT Research. Accessed 2024-05-15. Available from: https://github.com/nanoporetech/medaka.

[51] Fuhrmann L, Langer B, Topolsky I, Beerenwinkel N. VILOCA: Sequencing quality-aware haplotype reconstruction and mutation calling for short- and long-read data. bioRxiv. 2024;https://doi.org/10.1101/2024.06.06.597712.

[52] Zagordi O, Bhattacharya A, Eriksson N, Beerenwinkel N. ShoRAH: estimating the genetic diversity of a mixed sample from next-generation sequencing data. BMC bioinformatics. 2011;12(1):1–5.

[53] Eliseev A, Gibson KM, Avdeyev P, Novik D, Bendall ML, Pérez-Losada M, et al. Evaluation of haplotype callers for next-generation sequencing of viruses. Infection, Genetics and Evolution. 2020;82:104277.

[54] Li H. Aligning sequence reads, clone sequences and assembly contigs with BWA-MEM. arXiv preprint arXiv:13033997. 2013;.

[55] Langmead B, Salzberg SL. Fast gapped-read alignment with Bowtie 2. Nature methods. 2012;9(4):357–359.

[56] Li H. Minimap2: pairwise alignment for nucleotide sequences. Bioinformatics. 2018;34(18):3094–3100.

[57] Huang W, Li L, Myers JR, Marth GT. ART: a next-generation sequencing read simulator. Bioinformatics. 2012;28(4):593–594.

[58] Kruskal JB. Multidimensional scaling by optimizing goodness of fit to a nonmetric hypothesis. Psychometrika. 1964;29(1):1–27.

[59] Mikheenko A, Saveliev V, Gurevich A. MetaQUAST: evaluation of metagenome assemblies. Bioinformatics. 2016;32(7):1088–1090.

[60] V-pipe version 3.: Global haplotype reconstruction benchmarking study. Accessed 2023-10-02. Available from: https://github.com/cbg-ethz/V-pipe/tree/master/resources/auxiliary_workflows/benchmark/resources/multi_setup.

[61] Ahn S, Vikalo H. aBayesQR: a Bayesian method for reconstruction of viral populations characterized by low diversity. In: International Conference on Research

in Computational Molecular Biology. Springer; 2017. p. 353–369.

[62] Knyazev S, Tsyvina V, Shankar A, Melnyk A, Artyomenko A, Malygina T, et al. CliqueSNV: an efficient noise reduction technique for accurate assembly of viral variants from NGS data. bioRxiv. 2020;p. 264242.

[63] Töpfer A, Marschall T, Bull RA, Luciani F, Schönhuth A, Beerenwinkel N. Viral quasispecies assembly via maximal clique enumeration. PLoS computational biology. 2014;10(3):e1003515.

[64] Baaijens JA, Schönhuth A. Overlap graph-based generation of haplotigs for diploids and polyploids. Bioinformatics. 2019;35(21):4281–4289.

[65] Chen J, Zhao Y, Sun Y. De novo haplotype reconstruction in viral quasispecies using paired-end read guided path finding. Bioinformatics. 2018;34(17):2927–2935.

[66] Töpfer A, Zagordi O, Prabhakaran S, Roth V, Halperin E, Beerenwinkel N. Probabilistic inference of viral quasispecies subject to recombination. Journal of Computational Biology. 2013;20(2):113–123.

[67] Leviyang S, Griva I, Ita S, Johnson WE. A penalized regression approach to haplotype reconstruction of viral populations arising in early HIV/SIV infection. Bioinformatics. 2017;33(16):2455–2463.

[68] Fuhrmann L, Jablonski KP, Topolsky I, Batavia AA, Borgsmuller N, Baykal PI, et al .: V-pipe 3.0: a sustainable pipeline for within-sample viral genetic diversity estimation (Version 1). [Computer software]. Software Heritage. . Available from: https://archive.softwareheritage.org/swh:1:snp:a56f8dd7375288732aef62d97beb0675e1a1f422;origin=https://github.com/cbg-ethz/V-pipe.

[69] Elbe S, Buckland-Merrett G. Data, disease and diplomacy: GISAID's innovative contribution to global health. Global challenges. 2017;1(1):33–46.

[70] V-pipe version 3.: Mpox configuration example. Accessed 2023-10-03. Available from: https://github.com/cbg-ethz/V-pipe/blob/master/config/mpxv.yaml.

[71] Cantu VA, Sadural J, Edwards R. PRINSEQ++, a multi-threaded tool for fast and efficient quality control and preprocessing of sequencing datasets. PeerJ Preprints. 2019;7:e27553v1.

[72] Simon Andrews BB.: FastQC version 0.11.9. Accessed 2023-10-02. Available from: https://www.bioinformatics.babraham.ac.uk/projects/fastqc/.

[73] Danecek P, Marshall J, Danecek P, et al. HTSlib: C library for reading/writing high-throughput sequencing data. GigaScience. 2021;10:giab008.

[74] Vasimuddin M, Misra S, Li H, Aluru S. Efficient architecture-aware acceleration of BWA-MEM for multicore systems. In: 2019 IEEE international parallel and distributed processing symposium (IPDPS). IEEE; 2019. p. 314–324.

[75] Li H. A statistical framework for SNP calling, mutation discovery, association mapping and population genetical parameter estimation from sequencing data. Bioinformatics. 2011;27(21):2987–2993.

List of Figures

1 V-pipe 3.0 workflow overview. The data processing pipeline (left) provides four main steps: (1) preprocessing of the raw reads including quality control, (2) multiple sequence alignment, (3) estimation of viral diversity by SNV, local and global haplotype calling, and (4) if applicable, downstream analysis. The V-pipe 3.0 benchmarking module (right) supports the evaluation of viral diversity estimation methods on simulated data and on real experimental data where the ground truth diversity is known by the experimental design. For the simulated samples, first, ground truth haplotype populations are generated and based on those, sequencing reads are simulated. Then, the simulated and real samples are processed by the methods in the study, and last, the predicted viral diversity is compared to the ground truth viral diversity using different metrics for example precision, recall, f1, and N50 score. Left margins: V-pipe 3.0 is designed to facilitate efficient processing on personal computers as well as on computing clusters. It automatically sets up the necessary Conda environments, installs all dependencies, and initializes the project structure. It is also accessible through a Docker container, which includes all software dependencies. 36

2 Example configuration file for monkeypox virus. User-specified aligner, primer trimming method, and the method for the diversity estimation are defined in the `general` section. Input like reference genome, primer file, and the directory of the samples are specified in the `input` section. In section `preprocessing`, extra command line parameters are passed to the preprocessing step. In section `output`, users can define their desired output of the pipeline. This example configuration file is available on GitHub [70]. 37

3 Swiss surveillance of SARS-CoV-2 genomic variants using V-Pipe 3.0. **A)** Number of weekly submissions of SARS-CoV-2 consensus sequences from clinical samples to GISAID. **B)** Surveillance of SARS-CoV-2 variants in wastewater samples from ten locations in Switzerland with relative abundances of variants. **C)** Time-series of relative variant abundances with 95% confidence bands of wastewater samples from Zurich using V-pipe 3.0. 38

4 Benchmarking study for global haplotype reconstruction methods. **A)** Precision, recall, N50 score, and runtime for simulated samples of varying coverage of population 1. **B)** Left: MDS plots of one example simulation replicate per haplotype population. Each point represents a sequence. Symbol size corresponds to the frequency of the respective haplotype in the sample. HaploClique and HaploConduct were excluded due to their poor performance. Right: Precision and recall plots for each haplotype population. Each marker represents one replicate sample. **C)** N50, precision, recall, and f1 for PredictHaplo, CliqueSNV, and HaploConduct on a real HIV-5-virus mix. 39

5 **A)** Workflow for the performance evaluation of global haplotype reconstruction methods: 1. Generation of haplotype population based on user input, 2. Simulation of paired-end Illumina sequencing reads, 3. Run global haplotype reconstruction methods, 4. Performance evaluation. **B)** Generation of distance based haplotype populations: n_1: number of haplotypes in group one; n_2: number of haplotypes in group two; d_{12}: average pairwise distance between group one and two; d_1: average pairwise sequence distance within group one; d_2: average pairwise sequence distance within group two. **C)** Haplotype population parameter settings for the second synthetic dataset with constant coverage of 1000, and genome of length 10000. 40

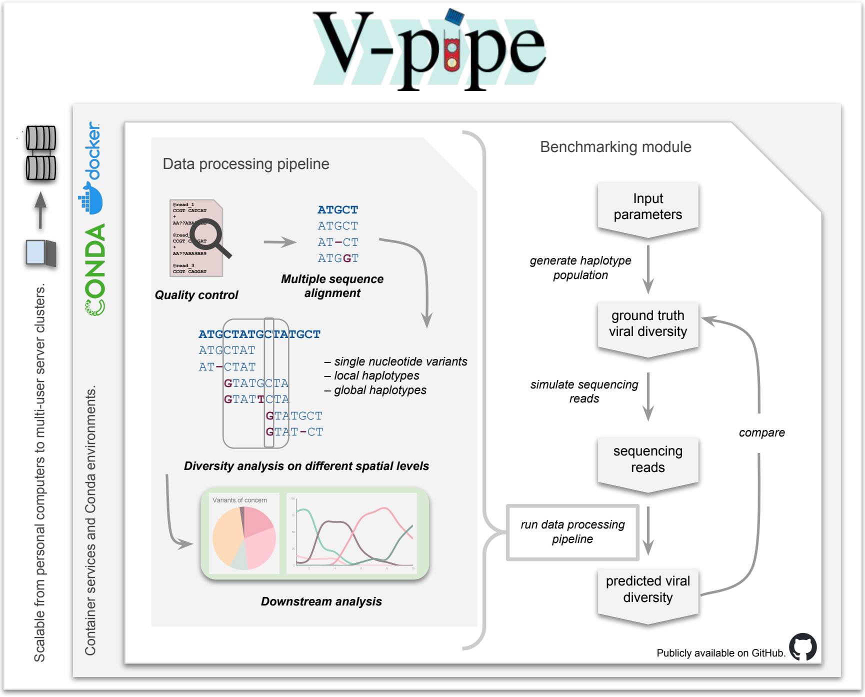

Fig. 1: V-pipe 3.0 workflow overview. The data processing pipeline (left) provides four main steps: (1) preprocessing of the raw reads including quality control, (2) multiple sequence alignment, (3) estimation of viral diversity by SNV, local and global haplotype calling, and (4) if applicable, downstream analysis. The V-pipe 3.0 benchmarking module (right) supports the evaluation of viral diversity estimation methods on simulated data and on real experimental data where the ground truth diversity is known by the experimental design. For the simulated samples, first, ground truth haplotype populations are generated and based on those, sequencing reads are simulated. Then, the simulated and real samples are processed by the methods in the study, and last, the predicted viral diversity is compared to the ground truth viral diversity using different metrics for example precision, recall, f1, and N50 score. Left margins: V-pipe 3.0 is designed to facilitate efficient processing on personal computers as well as on computing clusters. It automatically sets up the necessary Conda environments, installs all dependencies, and initializes the project structure. It is also accessible through a Docker container, which includes all software dependencies.

```yaml
name: MPXV

general:
    aligner: bwa
    primers_trimmer: samtools
    snv_caller: lofreq

input:
    reference: "{VPIPE_BASEDIR}/../resources/mpxv/MT903345.1.fasta"
    primers_file: "{VPIPE_BASEDIR}/../resources/mpxv/primers/MPXV-primer_genome-positions_subset.tsv"
    primers_bedfile: "{VPIPE_BASEDIR}/../resources/mpxv/primers/MPXV-primer_genome-positions_subset.bed"
    datadir: "{VPIPE_BASEDIR}/../resources/samples/"
    samples_file: samples.tsv

preprocessing:
    extra: -ns_max_n 4 -min_qual_mean 20 -trim_qual_left 20 -trim_qual_right 20 -trim_qual_window 10

output:
    trim_primers: true
    snv: true
    local: true
    global: false
    visualization: true
    QA: true
```

Fig. 2: Example configuration file for monkeypox virus. User-specified aligner, primer trimming method, and the method for the diversity estimation are defined in the `general` section. Input like reference genome, primer file, and the directory of the samples are specified in the `input` section. In section `preprocessing`, extra command line parameters are passed to the preprocessing step. In section `output`, users can define their desired output of the pipeline. This example configuration file is available on GitHub [70].

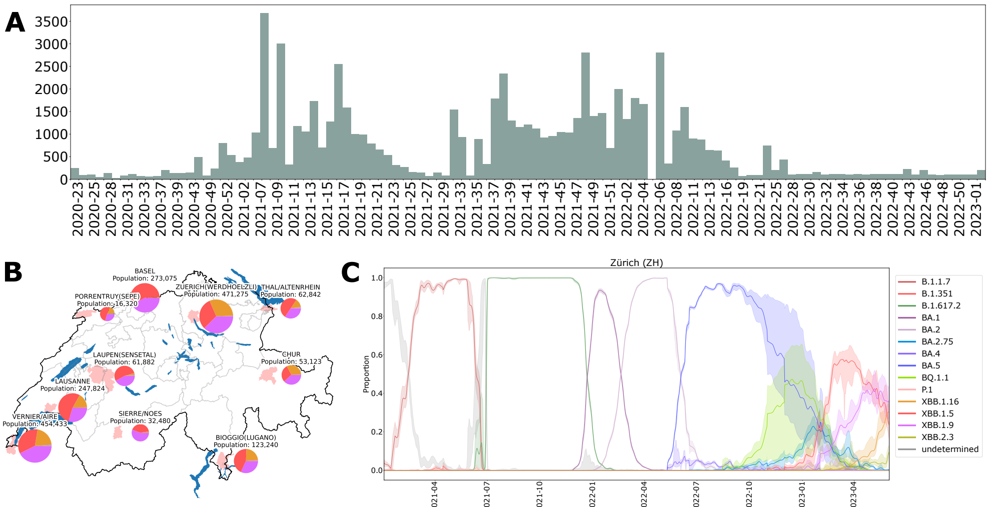

Fig. 3: Swiss surveillance of SARS-CoV-2 genomic variants using V-Pipe 3.0. **A)** Number of weekly submissions of SARS-CoV-2 consensus sequences from clinical samples to GISAID. **B)** Surveillance of SARS-CoV-2 variants in wastewater samples from ten locations in Switzerland with relative abundances of variants. **C)** Time-series of relative variant abundances with 95% confidence bands of wastewater samples from Zurich using V-pipe 3.0.

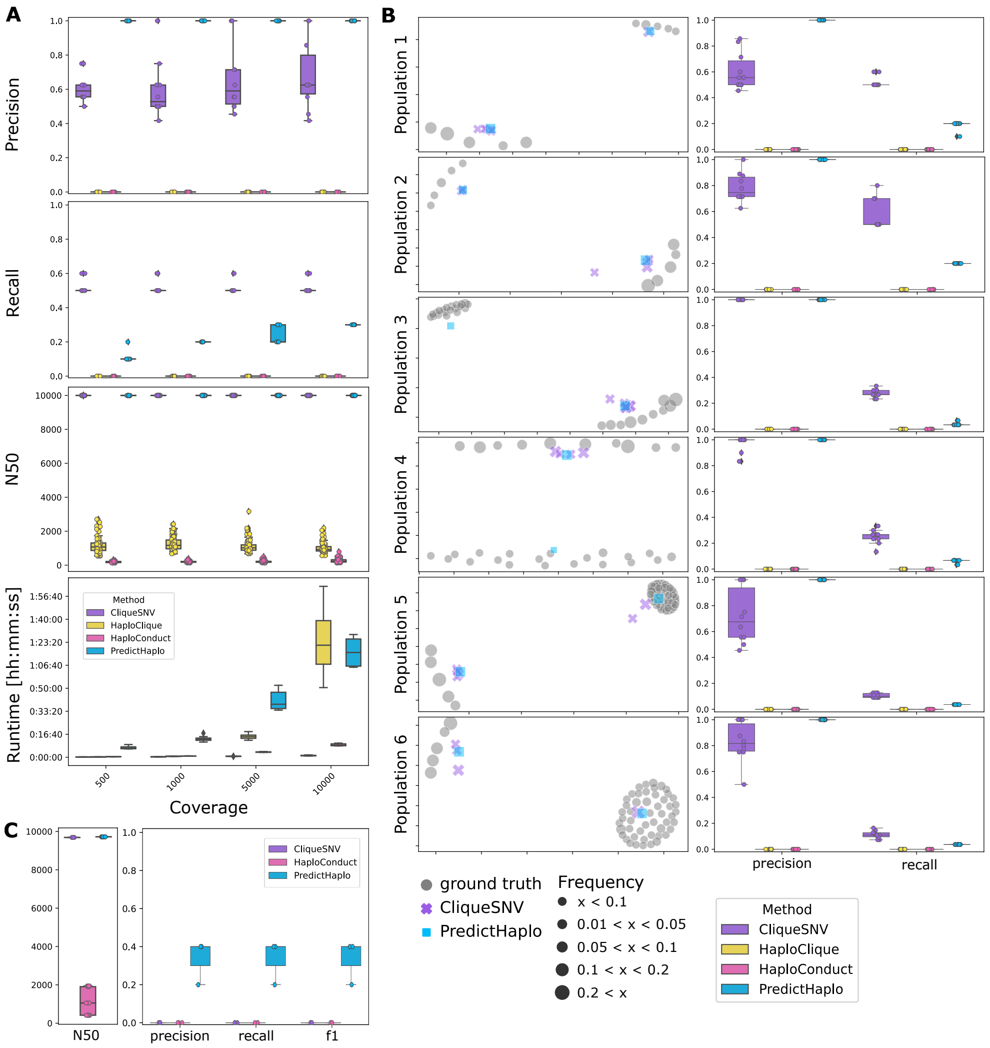

Fig. 4: Benchmarking study for global haplotype reconstruction methods. **A)** Precision, recall, N50 score, and runtime for simulated samples of varying coverage of population 1. **B)** Left: MDS plots of one example simulation replicate per haplotype population. Each point represents a sequence. Symbol size corresponds to the frequency of the respective haplotype in the sample. HaploClique and HaploConduct were excluded due to their poor performance. Right: Precision and recall plots for each haplotype population. Each marker represents one replicate sample. **C)** N50, precision, recall, and f1 for PredictHaplo, CliqueSNV, and HaploConduct on a real HIV-5-virus mix.

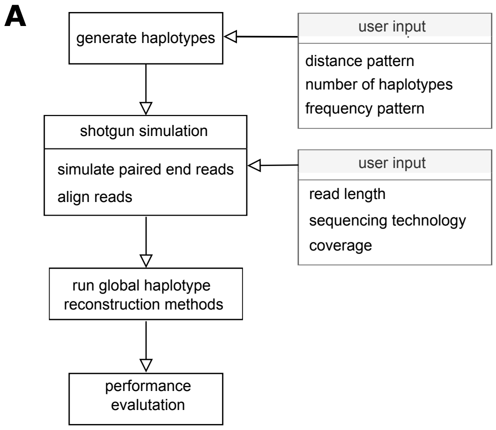

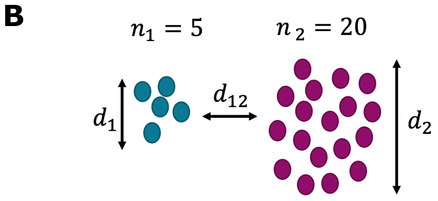

C

Population	n_1	n_2	d_{12}	d_1	d_2
1	5	5	200	50	20
2	5	5	400	100	100
3	10	20	200	50	20
4	10	20	400	100	100
5	5	50	200	50	20
6	5	50	400	100	100

Fig. 5: **A)** Workflow for the performance evaluation of global haplotype reconstruction methods: 1. Generation of haplotype population based on user input, 2. Simulation of paired-end Illumina sequencing reads, 3. Run global haplotype reconstruction methods, 4. Performance evaluation. **B)** Generation of distance based haplotype populations: n_1: number of haplotypes in group one; n_2: number of haplotypes in group two; d_{12}: average pairwise distance between group one and two; d_1: average pairwise sequence distance within group one; d_2: average pairwise sequence distance within group two. **C)** Haplotype population parameter settings for the second synthetic dataset with constant coverage of 1000, and genome of length 10000.

List of Tables

1 Methods and tools per data processing step that are integrated in V-Pipe 3.0 and the previous V-pipe workflow. Newly integrated tools into V-pipe 3.0 are marked in bold. 42
2 Comparison in terms of sustainability and functionalities of viral bioinformatics workflows for within-sample diversity estimation. Asteriks marks differences in the specfic tools integrated in V-pipe and V-pipe 3.0. 43

Data processing task	Tools in V-pipe 3.0	Tools in V-pipe
Quality control	PRINSEQ<br>FastQC<br>**'skip' quality control**	PRINSEQ ([71], RRID: SCR_005454)<br>FastQC ([72], RRID: SCR_014583)
De novo assembly	VICUNA	VICUNA ([46], RRID: SCR_006302)
Primer trimming	**IVar** ([47], RRID: SCR_024045)<br>**SAMtools** ([73], RRID: SCR_002105)	
Aligner	BWA MEM<br>Bowtie 2<br>ngshmmalign<br>**minimap2** ([56], RRID: SCR_018550)	BWA MEM ([74], RRID: SCR_022192)<br>Bowtie 2 ([55], RRID: SCR_016368)<br>ngshmmalign [8]
Paired-end reads merger	**SmallGenomeUtilites** [8]	
Consensus sequence generation	SmallGenomeUtilites<br>ngshmmalign<br>**BCFtools** ([73, 75], RRID: SCR_005227)	SmallGenomeUtilites [8]<br>ngshmmalign [8]
Frameshifts and stop codon diagnostics	**SmallGenomeUtilites** [8]	
Mutation calling	LoFreq<br>ShoRAH<br>**VILOCA** [51]	LoFreq ([49], RRID: SCR_013054)<br>ShoRAH ([52], RRID: SCR_005211)
Local haplotype reconstruction	ShoRAH<br>**VILOCA** [51]	ShoRAH [52]
Global haplotype reconstruction	**PredictHaplo** ([26], RRID: SCR_005207)<br>HaploConduct (SAVAGE)<br>HaploClique<br>**QuasiRecomb** ([66], RRID: SCR_008812)	 HaploConduct (SAVAGE) [64]<br>HaploClique [63]
Wasterwater surveillance	**COJAC** [6]<br>**LolliPop** [39]	

Table 1: Methods and tools per data processing step that are integrated in V-Pipe 3.0 and the previous V-pipe workflow. Newly integrated tools into V-pipe 3.0 are marked in bold.

	V-pipe 3.0	V-pipe	ViralFlow	nf-core/viralrecon	HAPHPIPE	GalaxyProject SARS-CoV-2 analysis effort
**Reproducibility**						
Automatic installation of all software dependencies	✓	✓	✓	✓	✗	✓
Container Services (e.g. Docker)	✓	✗	✓	✓	✗	✓
Automatic pipeline installation tests	✓	✗	✗	✓	✗	not applicable
Automatic pipeline execution tests on experimental samples	✓	✗	✗	✓	✗	✓
**Scalability**						
Dynamic cluster resource allocation	✓	✓	✓	✓	✗	✓
**Adaptability**						
Applicable for general viruses	✓	✓	✗	✓	✓	✗
Modular execution	✓	✓	✗	✓	✓	✓
Development: feature adding	✓	✓	✗	✓	✗	✓
**Transparency**						
Open source	✓	✓	✓	✓	✓	✓
Readability: pipeline code structure follow standard	✓	✗	✗	✓	✗	✓
Documentation	✓	✓	✓	✓	✓	✓
Examples	✓	✓	✓	✓	✓	✓
Tutorials	✓	✗	✓	✗	✓	✓
**Functionalities**						
De novo assembly	✓	✓	✗	✓	✓	✗
Read alignment	✓	✓	✓	✓	✓	✓
Consensus sequence generation	✓	✓(*)	✓	✓	✓	✓
Mutation calling	✓	✓(*)	✓	✓	✓	✓
Local haplotype reconstruction	✓	✓(*)	✗	✗	✗	✗
Global haplotype reconstruction	✓	✓(*)	✗	✗	✓	✗
SARS-CoV-2 wastewater surveillance	✓	✗	✓	✗	✗	✓
Benchmarking module	✓	✓	✗	✗	✗	✗

Table 2: Comparison in terms of sustainability and functionalities of viral bioinformatics workflows for within-sample diversity estimation. Asteriks marks differences in the specfic tools integrated in V-pipe and V-pipe 3.0.

Manuscript

Click here to access/download
Supplementary Material
Supplementary_File1.pdf
