# Supplementary material for: V-pipe 3.0: a sustainable pipeline for within-sample viral genetic diversity estimation
Source: Gigascience. 2024 Sep 30;13:giae065. doi: 10.1093/gigascience/giae065 (PMC11440432; doi:10.1093/gigascience/giae065)
Supplement: giae065_Response_to_Reviewer_Comments_Original_Submission [file giae065_response_to_reviewer_comments_original_submission.pdf]

# Author's Response To Reviewer Comments

We are first addressing the editor's comments, and then the specific reviewer's comments. Our answers are marked with “A:”.

# Editor's comments
– The 2 synthetic benchmark datasets generated should be made be made available.

A: The synthetic benchmark datasets can be reproduced using the Snakemake workflow available on GitHub [cite]. To ensure full reproducibility we have fixed the random seed for the generation of the synthetic benchmark datasets. We have added a step-by-step guide (https://github.com/cbg-ethz/V-pipe/tree/master/resources/auxiliary_workflows/benchmark/resources/multi_setup) to the GitHub repository that is explaining how to reproduce those datasets.

– The relevant command line scripts (or suitable methods document) used to run the comparative workflow/software tools including all parameters and versions should be made be made available.

A: The scripts to run the methods including all parameters and versions can be found on GitHub. We have added a README.md document (https://github.com/cbg-ethz/V-pipe/tree/master/resources/auxiliary_workflows/benchmark/resources/multi_setup) explaining the exact location of those scripts, including a step-by-step guide on how to reproduce the analysis including Figure 3 from the manuscript.

– For full transparency it would be very useful to provide a direct link to the 5-virus-mix sequence data mentioned here "We test the global haplotype reconstruction methods on sequencing reads from the 5-virus-mix presented in [15]." This would enable users to more easily locate and download the correct sequence data (I was unable to find it).

A: We have added the accession number of the 5-virus-mix sequencing data to the main text (line 504) and also to the ‘Availability of data and materials’ section (line 564).

– register V-pipe 3.0 in the bio.tools and SciCrunch.org databases to receive RRID (Research Resource Identification Initiative ID) and biotoolsID identifiers, and include these in your manuscript.

A: We have added the biotoolsID and the RRID to the manuscript in the section “Availability of data and materials”. While trying to update the entry on bio.tools the “Save” button gave us a “403 Request Blocked” error, we have contacted the developers but have not yet received an answer. We will update as soon as possible.

– Computational workflows should be registered in workflowhub.eu and the DOIs cited in the relevant places in the manuscript.

A: We have updated our entry on workflowhub.eu and have added the DOI in the section “Availability of data and materials”.

# Reviewers comments

Reviewer #1:
The manuscript showcases a computational pipeline designed for analyzing next generation sequencing data of short viral genomes, namely V-pipe 3.0. After an overview of the challenge the tool is addressing, i.e. the necessity of continuous benchmarking of various methods due to their diverse performance across different scenarios,the paper continues with a detailed listing of the results, highlighting the key elements of Reproducibility, Scalability, Adaptability and Transparency. The next section provides some details on the three applications / demonstrations of V-Pipe 3.0, i.e the Swiss SARS-CoV-2 Sequencing Consortium,

the Swiss surveillance of SARS-CoV-2 genomic variants in wastewater and the Global haplotype reconstruction benchmark. This is followed by a comprehensive comparison of V-Pipe 3.0 то other relevant viral bioinformatics pipelines for within sample diversity estimation, focusing on functionalities and sustainability, and specifically nf-core/viralrecon, HAPHPIPE and ViralFlow, as well as a section discussing the main advantages of V-Pipe 3.0 as well as the rationale for some of the identified drawbacks. The paper concludes with a thorough description of the underlying methods of V-Pipe 3.0 as well as on the data used.

Overall the paper gives a very good presentation of V-Pipe, and makes a strong case about its use and value in a real-world challenge. An overall comment is that there is some confusion on the role of V-Pipe 3.0 as a workflow - i.e. whether it's a dynamic system that uses different tools per step based on user input, or if it's an automated systems that benchmarks the analysis using (e.g.) synthetic data as the baseline.

A: Thank you for your comment. Indeed, the role of V-pipe 3.0 as a workflow and the new benchmarking module should be differentiated better. The benchmarking module is a new feature that was introduced in V-pipe 3.0. We have added the following sentence about V-pipe 3.0 to lines 113-116: “Depending on the user input, V-pipe 3.0 dynamically utilizes different tools for each processing step. In addition to the NGS data processing pipeline, we have incorporated a sub-workflow for benchmarking. This benchmarking module allows users to compare viral diversity estimation methods using synthetic and experimental data.”

In either case, there are also a few unclear points in the manuscript itself that could be further improved. Specifically:
-- It is not clear how V-pipe 3.0 differs from V-pipe. Although there is an indication of significant differences, an overview of the new features implemented in this version and/or a small introductory paragraph would be useful.

A: We have extended Table 1 to include V-pipe and highlight the new features. Additionally, we also include V-pipe in Table 2 and highlight the differences in terms of sustainability. Thank you for your suggestions, we believe they improve the clarity of the manuscript significantly.

-- In the "Results" section, lines 130 - 225 appear to refer to the implemented methodology and might be better served as part of the "Methods" section

A: While we acknowledge that this section of the Results may appear technical, we believe that its inclusion in the Results section is essential in this case as it reflects the outcomes of our development choice for the pipeline.

-- In the "Results" section, lines 135 - 138 implied that GitHub Actions are used to ensure Reproducibility of the workflow. Some more elaboration on this would be very useful, as GitHub actions are commonly used to automate processes (such as testing, conflict resolution etc). In particular, an reproducibility issue that might not be resolvable by GitHub actions are dependency conflicts that are specific to the particular system that is being tested.

A: Thank you for this comment. We have written workflow scripts that are executed via GitHub Actions to perform installation and execution tests. To improve clarity, we have added the following sentence in lines 142-147 including a direct reference to the definition of those workflow scripts:
“To ensure successful installation and reproducible execution on different systems, we have written GitHub Actions [19], workflow scripts [20] which automatically perform test installations of V-pipe 3.0 on Mac OS and Linux systems and run end-to-end tests by executing tutorials with real example data. For each update of V-pipe 3.0 these workflow scripts are automatically executed and report about installation problems or issues on the test data.”

-- In the "Results" section, lines 139 - 146, it's not clear how the benchmark study contributes to the overall reproducibility of V-pipe 3.0. Some further explanation of the rationale would be very useful here.

A: Thank you for this comment. We agree that this paragraph can be improved. We have rewritten the paragraph (lines 148-158):

“Additionally, V-pipe 3.0 enables reproducible benchmarking of viral diversity estimation methods using the newly integrated benchmarking module, a critical capability considering the constant evolution of new methods and the availability of new datasets. The benchmarking module is a Snakemake based workflow which automatically applies a set of selected tools to various synthetic and real data sets, computes their respective performances in terms of precision and recall, and summarizes the results. The benchmarking workflow is itself sustainably implemented. Adding new tools and data sets to this benchmark is very easy and only requires the addition of a single file and no further modifications of the workflow. As a concrete demonstration of the effectiveness of the benchmarking module, we conducted a benchmarking study focused on global haplotype reconstruction (Section 3.3 below).”

-- In the "Results" section, lines 179 - 183, it is not clear how Git and GitHub ensure adaptability of any new features that are implemented. Usually a version control system/automation system, can facilitate the integration of new features, but it's not readily evident how it supports/ensures/facilitates adaptability. Maybe a definition of "adaptability" in this particular context could also help.

A: Thank you for this comment! We agree that the mentioning of version control at this point is rather confusing. We have removed lines 179-183 and added a compromised version in lines 194-198 to make the point that the integration of new features is tracked and tested to ensure smooth integration of new features. Below is the sentence we added to lines 190-194:
“Additionally, we run automated integration and unit tests using GitHub Actions workflows [19, 20] on every commit submitted to the repository. This verifies the reliability, consistency and correctness of the overall workflow. We use datasets from different viruses in our tests to make sure that V-pipe 3.0 and the newly added features are running successfully from start to end.”

-- In the "Applications" section, it is not clear which version of V-Pipe was used for the overall analysis (V-pipe or V-pipe 3.0), especially in the wastewater use case.

A: This comment is very valid. Indeed V-pipe 3.0 was developed in the course of the SARS-CoV-2 pandemic to address the new challenges not only in terms of scalability and the need of new features, but also the need to produce reproducible and transparent results. To clarify this we have added a sentence to the introduction lines 102-105:
“V-pipe 3.0 builds upon the foundation of V-pipe [8], but has undergone significant extensions and refinements to increase functionality and to adhere to sustainable data processing standards [16]. The development of V-pipe 3.0 was primarily driven to address the new demands and challenges that became evident in the SARS-CoV-2 pandemic.”
Additionally, we clarify in the section “Applications” that the whole cohort of samples was reanalysed upon each update of V-pipe. Hence, the results presented were generated with the newest release of V-pipe 3.0. We have added the following sentences to the manuscript for clarification (lines 228-233):
“The development of V-pipe 3.0 was primarily driven by two large-scale SARS-CoV-2 surveillance projects. Continuous updates and extensions of the pipeline have been motivated by the evolving demands of the pandemic. With each workflow update, the entire sample cohort underwent reanalysis using the latest pipeline version. This approach guaranteed consistent results that align with the most recent advancements in the pipeline.”

-- In the section "Comparison to other workflows" it is not very clear which tools are implemented within V-pipe 3.0, which differences there are with previous version (V-pipe) and how these differ to other pipelines. A table that is summarizing these details and highlighting the differences would be very useful here.

A: Thank you for your comment. We have included a column listing the tools from the previous V-pipe in Table 1, with new features added in V-pipe 3.0 highlighted in bold. Furthermore, the original V-pipe has been incorporated into the comparison table (Table 2) to emphasize distinctions in reproducibility, scalability, adaptability, and transparency.

Moreover, there are a few minor points that would enhance the readers' understanding:
-- (minor) In the Section "2.1 Reproducibility", it's mentioned that all software dependencies are defined in Conda environments, making V-pipe 3.0 portable between different computing platforms. Is there a particular reason why V-Pipe itself isn't implemented as a conda package directly?

A: As V-pipe 3.0 is a Snakemake workflow it is rather unintuitive to package it as a Conda package. Snakemake itself manages the workflow's execution and dependencies, and it is primarily focused on defining and running workflows rather than serving as a distributable package. Hence, we found that packaging it into a Conda environment may introduce unnecessary complexity without significant advantages in our specific context.

-- (minor) More often that not, the pandemic is named as COVID19, in contrast to the virus that is named "SARS-CoV-2". It may be useful to amend/update the references to the "SARS-CoV-2 pandemic" accordingly.

A: We agree that the use of the “SARS-CoV-2 pandemic” is more appropriate.

Reviewer #2:
V-pipe 3.0 is introduced as an advanced computational pipeline tailored for the analysis of next-generation sequencing data from short viral genomes. Designed to meet the challenges posed by the vast and diverse datasets generated by these technologies, V-pipe 3.0 emphasizes reproducibility, scalability, adaptability, and transparency. It achieves this by adhering to Snakemake's best practices, allowing easy swapping of virus-specific configuration files, and providing thoroughly tested examples online. The utility of V-pipe 3.0 is showcased through its application in two extensive data analysis projects, proving its efficacy in sustainable viral genomic data science.
Central to V-pipe 3.0 is its capacity for estimating viral diversity from sequencing data. A versatile benchmarking module has been developed to continuously assess various diversity estimation methods, accommodating the rapid advancements within this field. The pipeline simplifies the inclusion of new tools and datasets, supporting both synthetic and real experimental data. However, challenges in global haplotype reconstruction highlight the need for scalable methods that can accurately reflect the complex population structures of viruses and manage the uncertainties in the results.
Some additional clarification in the manuscript would be appreciated.

1) I'm curious about how the efficiency is attained.

A: To achieve scalability on extremely large cohorts, efficient resource usage (memory, cpu) per sample is necessary. To achieve this several measures were implemented, firstly, V-pipe 3.0 dynamically specifies cluster resources to adapt to the specific data requirements, second, V-pipe 3.0 enables the parallel execution of unrelated data analysis steps, third, V-pipe 3.0 validates user configuration files using JSON Schema during startup to identify potential runtime errors early and lastly, V-pipe 3.0 splits centralized tasks among multiple compute nodes and perform per-sample distributed computation of summary statistic (see Section Results - Scalability lines 163-171).

2) Is it possible to utilize V-pipe for analyzing other microorganisms?

A: Yes, this depends on the tools selected. While bacteria may require tools different from the currently implemented ones, V-pipe 3.0 has successfully processed sequencing data from various different viruses, including, for example, the DNA virus HSV2 (Lezcano, Oscar M., et al. Parallel evolution and enhanced virulence upon in vivo passage of an RNA virus in Drosophila melanogaster. Virus Evolution 9.2 (2023)).

3) The authors might consider directing readers to the following review article for reference:
https://genomebiology.biomedcentral.com/articles/10.1186/s13059-021-02328-9

A: Thank you for recommending this review article. However, we feel that directing readers to chromosome-scale haplotype reconstruction methods might lead to confusion, as this topic is not addressed by V-pipe 3.0.

4) Identifying specific genes or genome regions with high polymorphism across different populations would be fascinating. How does V-pipe handle analysis in these highly variable regions?

A: V-pipe 3.0 enables the analysis of highly variable regions by integrating tools that can handle such

scenarios. For example, the alignment tool ngshmmalign is especially useful if long deletions and high heterogeneity are present (Posada-Céspedes, Susana, et al. V-pipe: a computational pipeline for assessing viral genetic diversity from high-throughput data. Bioinformatics 37.12 (2021): 1673-1680).
Ultilimety, the handling of V-pipe 3.0 depends on the users tools choice, hence we provide various tools to choose from in the workflow that have their advantages and disadvantages in different settings (see section 4).
We have added a sentence in line 412-414 to emphasize this: “For example, for alignment V-pipe 3.0 supports BWA MEM, Bowtie 2, ngshmmalgin and minimap2 which allows the processing of samples with very high diversity regions.”. Further, our variety of haplotype reconstruction tools as listed in Table 1 enable the analysis of very diverse datasets.

Reviewer #3:
Review of Article: "V-pipe 3.0: A Sustainable Workflow for Diversity Estimation from Viral NGS Samples"
General Assessment
The article presents V-pipe 3.0, a Snakemake-based bioinformatics workflow designed for analyzing intra-sample variability in viral Next-Generation Sequencing (NGS) data. While the authors emphasize sustainability and scalability, several critical scientific concerns undermine the credibility and utility of the presented work.

Scientific Concerns
1. Poor Availability of Hypotheses, Results and Interpretations/Conclusions
The article lacks clarity regarding the actual results obtained from the implementation of V-pipe 3.0. While the authors mention the application of the workflow in large-scale projects ("Applications" 3.1, 3.2 and 3.3), specific outcomes, such as the accuracy of genetic evolution or diversity estimations or the identification of viral variants, are poorly presented.
Readers require concrete questions, findings and statistical analyses to assess the efficacy and reliability of the workflow. Without transparent reporting of results, the scientific value of the article diminishes significantly.
2. Ill-defined Hypotheses and Scientific Questions
The article fails to clearly articulate the underlying hypotheses or scientific questions addressed by V-pipe 3.0. While it outlines the technical aspects and functionalities of the workflow, the overarching scientific goals remain obscure.
Without well-defined research questions, it becomes challenging for readers to contextualize the significance of the presented work within the broader field of virology or computational biology.

A: Thank you for your comment. In this manuscript, we have presented V-pipe 3.0, a robust data processing pipeline for the analysis of heterogeneous viral NGS samples. Our primary objective is to establish sustainable data processing practices (section 1), resulting in the development of a versatile pipeline that caters to a comprehensive spectrum of diversity estimation tasks of mixed samples. Sustainability is demonstrated in terms of reproducibility (section 2.1), scalability which relies on the efficacy of the workflow (section 2.2), adaptability (section 2.3), and transparency (section 2.4). We demonstrate its applicability and practicalness by providing three applications (section 3). Two large-scale projects are presented where V-pipe 3.0 proved to enable sustainable and reliable data processing (see section 3.1 and 3.2), and the global haplotype reconstruction study is exemplary of the newly integrated benchmarking module (see section 3.3). We illustrate the practical utility and reliability of our pipeline, as well as the transparency of the results. These applications provide valuable insights into the pipeline's capabilities in addressing biological and virological research inquiries, solidifying the claim of a reliable solution for viral diversity estimation. Hence the results of this manuscript are not the results of the large-scale projects but rather the implementation and successful application to the large-scale projects.

3. Unverifiable Conclusions
The article concludes with a comparison of V-pipe 3.0 to other workflows, highlighting its purported advantages in terms of sustainability and functionality. However, the evaluation lacks empirical evidence or comparative analyses to substantiate these claims.
Readers are unable to independently verify the superiority of V-pipe 3.0 over alternative workflows, thereby undermining the credibility of the conclusions drawn by the authors.
There is a missing mark that is difficult to understand in this comparison, which casts a shadow over its

relevance. Several Galaxy workflows were used, very early during the SARS-CoV-2 pandemic (first publication in August 2020 in PLoS Pathogens), to monitor the emergence of viral variants or assess intra-host variations. These workflows are still actively used and regularly updated (https://galaxyproject.org/projects/covid19/).

A: Thank you for your comments and suggestions. In crafting the comparison table (Table 2), we meticulously applied transparent criteria as listed in the table and as further elucidated with explanations and illustrative examples in the main text, lines 355-406. These criteria can be independently checked by anyone who is interested by reading the corresponding code repositories, documentation, and research articles of the workflows included in the comparison.
To the best of our knowledge the submitted Table 2 is not missing any marks and is complete.
Thank you for making us aware of the Galaxy workflows, indeed they belong in our comparison table. Our inclusion criteria for a workflow to be integrated into the comparison is that it needs to be open source, actively maintained, and provide within-sample diversity estimates for Illumina sequencing reads. Hence, we have included the GalaxyProject SARS-CoV-2 analysis effort workflows (https://galaxyproject.org/projects/covid19/workflows/). Since they are usable together we are treating them together as one workflow. We have added also the original V-pipe pipeline to the comparison for clarification of the differences to V-pipe 3.0 as suggested by Reviewer #1. Hence, two new columns were added to Table 2: "V-pipe" and "GalaxyProject SARS-CoV-2 analysis effort".
Additionally, we have adapted the corresponding Section 4, lines 356-414, in the manuscript to highlight the properties of the GalaxyProject workflows. We believe that these additions are substantially improving the value of the comparison. Thank you for your suggestions.

Technical Concerns
A limited number of target users
V-pipe can only be installed by researchers familiar with the use of a Bash script in a Linux terminal and the concepts of environments and dependencies. Once installed, the workflow must be configured to adapt it to specific needs, recognizing that most virologists have very different goals from national-level monitoring of intra- or inter-host variations. However, this configuration involves editing a yaml script and clearly requires relatively advanced programming skills given that V-pipe is complex software. Finally, even if a configuration file was built by a computer scientist, the correct execution of V-pipe, the access to the results as well as the verification that these results are computationally consistent can only be done by a person who is familiar with a Linux console and command lines to interact with. In the end, unfortunately, I do not believe that V-pipe will be useful to a large audience among virologists.

A: Indeed, V-pipe 3.0 requires computer literacy and basic command line knowledge. Virologists without any bioinformatics knowledge are not our target audience. However, in depth knowledge about environments and dependencies is not needed as V-pipe 3.0 is automatically generating, managing and activating the necessary environments. This is the advantage of workflow systems like Snakemeke which is the system V-pipe 3.0 is built on.
Also the yaml-file configuration does not require any advanced programming knowledge, but solely requires users to essentially be able to use a text editor. Subsequently, freely available online yaml validation tools can be used that can help users validate their modified yaml files.
In order to access the results no linux console or command line knowledge is needed as results and also verification of the successful execution can be accessed through simply clicking through the result directories and opening the respective error text files.
We do not require users to check for "computational consistency". We test parts of the computational consistency automatically as described in Section "Reproducibility" which is tested upstream before release (see lines 142-147).

As it stands, V-pipe workflows are difficult to reproduce.
Given the objectives stated in this work, I obviously expected to be able to reproduce the analyses presented in section 3 (Applications), or to be able to run - with V-pipe - a pilot analysis used for comparison with other workflows. Unless I am mistaken, I have found, neither in the manuscript nor in the GitHub repository, any configuration file and the associated input data to reproduce, at least partially, even one figure of the article.

A: Thank you for making us aware that the benchmark study is not easy to find on the GitHub repository. We are sorry to hear that you were unable to find the Snakemake workflow reproducing the benchmark study. In the original submission it was linked in the section "Availability of data and materials." Now, we added the reference also to the section "Methods - Global haplotype reconstruction benchmark study". In this GitHub repository directory, readers can find all configuration scripts needed to reproduce the analysis. This includes configuration files defining the simulation of the simulated data and scripts for the execution of the methods. Further the notebooks to generate the figures from Figure 3 in the manuscript are also available in this repository under "workflow/notebooks", following the Snakemake workflow directory standards.
Additionally, we have added a step-by-step guide to reproduce the figures from Figure 3 in the manuscript which is also available in the GitHub repository directory (V-pipe/resources/auxiliary_workflows/benchmark/resources/multi_setup) under README.md.

If we focus only on the V-pipe GitHub repository, we find the necessary documentation to install it easily (with the caveats mentioned above) but there is no obvious example to test the use of V-pipe.

A: Thank you for your comment. We have two tutorials available on our GitHub repository that enable users to test the use of V-pipe 3.0 on two experimental datasets. We have updated the GitHub README such that the tutorials are now linked more prominently. Additionally, we have added a direct link to the two tutorials to the section "Results - Transparency" in line 216.

I'm lucky enough to be very familiar with GitHub Actions and so I looked for a testing workflow in these Actions that could serve as an example for a local test of V-pipe. I found the script "V-pipe/tests/regression_tests.sh", but its contents did not reassure me at all about the transparency of V-pipe workflows.

A: Thank you for this comment. As also reviewer #1 mentioned, the use of GitHub Actions for our testing needs to be described better. We have written workflow scripts that are executed via GitHub Actions to perform installation and execution tests. To improve clarity, we have added the following sentence in lines 142-147 including a direct reference to the definition of those workflow scripts (they are not in "V-pipe/tests/regression_tests.sh"):
"To ensure successful installation and reproducible execution on different systems, we have written GitHub Actions [19], workflow scripts [20] which automatically perform test installations of V-pipe 3.0 on Mac OS and Linux systems and run end-to-end tests by executing tutorials with real example data. For each update of V-pipe 3.0 these workflow scripts are automatically executed and report about installation problems or issues on the test data."
Additionally, we added a README.md file to the GitHub directory "https://github.com/cbg-ethz/V-pipe/tree/master/tests" that points interested readers to the correct directory of all the workflow testing scripts. Those are in "https://github.com/cbg-ethz/V-pipe/tree/master/.github/workflows". We have also added a README.md file for clarity to this directory: "https://github.com/cbg-ethz/V-pipe/blob/master/.github/workflows/README.md"

Clearly missing from the GitHub repository is a concrete example of using V-pipe with real input data and a step-by-step explanation of configuring Snakemake and running a workflow.

A: Thank you for pointing out that our tutorials with step-by-step explanations are not easily visible on our GitHub repository. We have two tutorials available on our GitHub repository that enable users to test the use of V-pipe 3.0 on two experimental datasets. These tutorials are step-by-step guides explaining the installation process, organization of the data, preparing the data, preparing the configuration, running V-pipe and analyzing the output.
We have updated the GitHub README such that the tutorials are now linked more prominently. Additionally, we have added a direct link to the two tutorials to the section "Results - Transparency" in line 216.

Recommendations
1. Enhanced Results Reporting
- The authors should provide detailed summaries of empirical findings obtained through the application of

V-pipe 3.0. This includes accuracy metrics, performance benchmarks, and comparative analyses with existing methods.
- Statistical measures, such as precision, recall, and computational efficiency, should be clearly presented to enable rigorous evaluation by the scientific community.

A: In this manuscript, we have presented a workflow that enables sustainable data analysis, as detailed in the "Results" section. As V-pipe 3.0 offers various options of tools for each data processing step, the performance (like precision, recall, accuracy, etc) is given by the chosen tool. It is known that different tools excel in distinct scenarios, each with its set of advantages and limitations. Hence, it is the tools that need to prove their performance with statistical metrics such as precision and recall. The primary objective of a pipeline like V-pipe 3.0 is to ensure sustainable data processing. We have demonstrated sustainability of the pipeline by following the four aspects in Mölder's hierarchy: reproducibility (section 2.1), scalability (section 2.2), adaptability (section 2.3) and transparency (section 2.4). For each of the four aspects, we demonstrate the various developmental choices made to guarantee and achieve sustainability of V-pipe 3.0. Additionally, we prove the practicality of V-pipe 3.0 in real life scenarios by presenting three applications (section 3).

2. Clarification of Research Objectives
- Prioritize the elucidation of specific research questions or hypotheses addressed by V-pipe 3.0. Clearly articulate the scientific motivations behind the development and implementation of the workflow.
- By defining clear objectives, the authors can enhance the relevance and impact of their work within the field of viral genomics and bioinformatics.

A: Thank you for your comment. The primary motivation of the development of V-pipe 3.0 were the new challenges and demands that became evident with the onset of the SARS-CoV-2 pandemic. On the one hand this was the need of new features, like primer trimming or the frameshifts and stop codon diagnostics (Table 1). On the other hand, the large amount of newly available data and also the impact of analysis results, as we reported to public health decision makers, required sustainable data analysis. We have clarified this in the introduction in lines 102-106:
"V-pipe 3.0 builds upon the foundation of V-pipe [8], but has undergone significant extensions and refinements to increase functionality and to adhere to sustainable data processing standards [16]. The development of V-pipe 3.0 was primarily driven to address the new demands and challenges that became evident in the SARS-CoV-2 pandemic."
Additionally, we emphasize this objective by adding the following sentences to the Applications section in lines 228-233 for further clarification:
"The development of V-pipe 3.0 was primarily driven by two large-scale SARS-CoV-2 surveillance projects. Continuous updates and extensions of the pipeline have been motivated by the evolving demands of the pandemic. With each workflow update, the entire sample cohort underwent reanalysis using the latest pipeline version. This approach guaranteed consistent results that align with the most recent advancements in the pipeline."

3. Empirical Validation of Conclusions
- Substantiate claims regarding the superiority of V-pipe 3.0 through rigorous empirical validation. Conduct thorough comparative analyses with alternative workflows, incorporating diverse datasets and performance metrics.
- Transparently report the results of benchmarking studies and provide access to raw data and code repositories to facilitate reproducibility and independent verification.

A: Thank you for this comment. With V-pipe 3.0 providing multiple tool options for each data processing step, the choice of tool determines performance metrics such as precision and recall. Also other workflows like HAPHPIPE (see section 4) provide various methods and tools per processing step. Hence a computational comparative analysis would not be meaningful as performance depends on the user-selected tools. In order to still provide an informative comparison, we have included the comparison table Table 2. This table allows readers to compare workflows with comparable purpose, to get an overview over the implemented features and also the sustainability of those workflows. Following your suggestion, we have also extended this table to include another relevant workflow that is hosted by Galaxy.
Additionally, to address your suggestion regarding the transparency of the benchmark study results, we

have added a step-by-step guide on how to reproduce the benchmarking study to GitHub repository: https://github.com/cbg-ethz/V-pipe/tree/master/resources/auxiliary_workflows/benchmark/resources/multi_setup

Conclusion
While the article introduces an ambitious bioinformatics workflow for analyzing viral NGS data, several scientific deficiencies undermine its credibility and utility. By addressing concerns related to results reporting, hypothesis formulation, and conclusion verification, the authors can enhance the scientific rigor and impact of their work.
In its current state, implementation of V-pipe in research projects is lacking detailed instructions if a large audience of biologists is targeted. Ultimately the question is not so much whether V-pipe workflows are reproducible, scalable, adaptable and transparent, but rather for whom. In my opinion, additional work on the general architecture of the software and its documentation can still significantly improve these 4 aspects in order to reach a wider audience of virologists.

A: We would like to thank the reviewer for their useful comments. We followed your suggestions on extending Table 2 and included the GalaxyProject SARS-CoV-2 analysis effort workflows (https://galaxyproject.org/projects/covid19/workflows/) into our comparison. Further, for transparency of the benchmark study results, we have added a step-by-step guide to the GitHub as a README in the directory https://github.com/cbg-ethz/V-pipe/tree/master/resources/auxiliary_workflows/benchmark/resources/multi_setup. This gives clear instructions on how to reproduce the analysis and the figures. All configuration files to produce the simulated data and all scripts executing the methods are available in this directory. Additionally, instead of only referring to the GitHub repository in the section “Availability of data and materials”, we have also added a direct link to the section “Methods”. Further to improve user experience we have updated the README on the V-pipe repository (https://github.com/cbg-ethz/V-pipe) with a more prominent link to the tutorials with two experimental datasets. Additionally, we have added a direct link to the two tutorials to the section “Results - Transparency” in line 216.
Moreover, we have improved the explanations on our testing structure using GitHub Actions. We have written workflow scripts that are executed via GitHub Actions to perform installation and execution tests, this is elaborated in lines 142-147 including a direct link to the definition of those workflow scripts (https://github.com/cbg-ethz/V-pipe/tree/master/.github/workflows). To guide interested readers better, we have also added READMEs to the GitHub directory explaining which tests are run.
