# Supplementary material for: V-pipe 3.0: a sustainable pipeline for within-sample viral genetic diversity estimation
Source: Gigascience. 2024 Sep 30;13:giae065. doi: 10.1093/gigascience/giae065 (PMC11440432; doi:10.1093/gigascience/giae065)
Supplement: giae065_Reviewer_1_Report_Original_Submission — Fotis Psomopoulos, Ph.D. -- 1/8/2024 Reviewed [file giae065_reviewer_1_report_original_submission.pdf]

**Reviewer Report**

**Title: V-pipe 3.0: a sustainable pipeline for within-sample viral genetic diversity estimation**

**Version: Original Submission** **Date:** 1/8/2024

**Reviewer name: Fotis Psomopoulos, Ph.D.**

**Reviewer Comments to Author:**

The manuscript showcases a computational pipeline designed for analyzing next generation sequencing data of short viral genomes, namely V-pipe 3.0. After an overview of the challenge the tool is addressing, i.e. the necessity of continuous benchmarking of various methods due to their diverse performance across different scenarios,the paper continues with a detailed listing of the results, highlighting the key elements of Reproducibility, Scalability, Adaptability and Transparency. The next section provides some details on the three applications / demonstrations of V-Pipe 3.0, i.e the Swiss SARS-CoV-2 Sequencing Consortium, the Swiss surveillance of SARS-CoV-2 genomic variants in wastewater and the Global haplotype reconstruction benchmark. This is followed by a comprehensive comparison of V-Pipe 3.0 Ï„Î¿ other relevant viral bioinformatics pipelines for within sample diversity estimation, focusing on functionalities and sustainability, and specifically nf-core/viralrecon, HAPHPIPE and ViralFlow, as well as a section discussing the main advantages of V-Pipe 3.0 as well as the rationale for some of the identified drawbacks. The paper concludes with a thorough description of the underlying methods of V-Pipe 3.0 as well as on the data used.
Overall the paper gives a very good presentation of V-Pipe, and makes a strong case about its use and value in a real-world challenge. An overall comment is that there is some confusion on the role of V-Pipe 3.0 as a workflow - i.e. whether it's a dynamic system that uses different tools per step based on user input, or if it's an automated systems that benchmarks the analysis using (e.g.) synthetic data as the baseline. In either case, there are also a few unclear points in the manuscript itself that could be further improved. Specifically:
-- It is not clear how V-pipe 3.0 differs from V-pipe. Although there is an indication of significant differences, an overview of the new features implemented in this version and/or a small introductory paragraph would be useful.
-- In the "Results" section, lines 130 - 225 appear to refer to the implemented methodology and might be better served as part of the "Methods" section
-- In the "Results" section, lines 135 - 138 implied that GitHub Actions are used to ensure Reproducibility of the workflow. Some more elaboration on this would be very useful, as GitHub actions are commonly used to automate processes (such as testing, conflict resolution etc). In particular, an reproducibility issue that might not be resolvable by GitHub actions are dependency conflicts that are specific to the particular system that is being tested.
-- In the "Results" section, lines 139 - 146, it's not clear how the benchmark study contributes to the overall reproducibility of V-pipe 3.0. Some further explanation of the rationale would be very useful here.
-- In the "Results" section, lines 179 - 183, it is not clear how Git and GitHub ensure adaptability of any

new features that are implemented. Usually a version control system/automation system, can facilitate the integration of new features, but it's not readily evident how it supports/ensures/facilitates adaptability. Maybe a definition of "adaptability" in this particular context could also help.
-- In the "Applications" section, it is not clear which version of V-Pipe was used for the overall analysis (V-pipe or V-pipe 3.0), especially in the wastewater use case.
-- In the section "Comparison to other workflows" it is not very clear which tools are implemented within V-pipe 3.0, which differences there are with previous version (V-pipe) and how these differ to other pipelines. A table that is summarizing these details and highlighting the differences would be very useful here.
Moreover, there are a few minor points that would enhance the readers' understanding:
-- (minor) In the Section "2.1 Reproducibility", it's mentioned that all software dependencies are defined in Conda environments, making V-pipe 3.0 portable between different computing platforms. Is there a particular reason why V-Pipe itself isn't implemented as a conda package directly?
-- (minor) More often that not, the pandemic is named as COVID19, in contrast to the virus that is named "SARS-CoV-2". It may be useful to amend/update the references to the "SARS-CoV-2 pandemic" accordingly.

**Methods**

Are the methods appropriate to the aims of the study, are they well described, and are necessary controls included? Choose an item.

**Conclusions**

Are the conclusions adequately supported by the data shown? Choose an item.

**Reporting Standards**

Does the manuscript adhere to the journal's guidelines on minimum standards of reporting? Choose an item.

Choose an item.

**Statistics**

Are you able to assess all statistics in the manuscript, including the appropriateness of statistical tests used? Choose an item.

**Quality of Written English**

Please indicate the quality of language in the manuscript: Choose an item.

**Declaration of Competing Interests**

Please complete a declaration of competing interests, considering the following questions:

- Have you in the past five years received reimbursements, fees, funding, or salary from an organisation that may in any way gain or lose financially from the publication of this manuscript, either now or in the future?
- Do you hold any stocks or shares in an organisation that may in any way gain or lose financially from the publication of this manuscript, either now or in the future?
- Do you hold or are you currently applying for any patents relating to the content of the manuscript?
- Have you received reimbursements, fees, funding, or salary from an organization that holds or has applied for patents relating to the content of the manuscript?
- Do you have any other financial competing interests?
- Do you have any non-financial competing interests in relation to this paper?

If you can answer no to all of the above, write 'I declare that I have no competing interests' below. If your reply is yes to any, please give details below.

I declare that I have no competing interests

I agree to the open peer review policy of the journal. I understand that my name will be included on my report to the authors and, if the manuscript is accepted for publication, my named report including any attachments I upload will be posted on the website along with the authors' responses. I agree for my report to be made available under an Open Access Creative Commons CC-BY license (http://creativecommons.org/licenses/by/4.0/). I understand that any comments which I do not wish to be included in my named report can be included as confidential comments to the editors, which will not be published.

Choose an item.

To further support our reviewers, we have joined with Publons, where you can gain additional credit to further highlight your hard work (see: https://publons.com/journal/530/gigascience). On publication of this paper, your review will be automatically added to Publons, you can then choose whether or not to claim your Publons credit. I understand this statement.

Yes Choose an item.
