# Supplementary material for: V-pipe 3.0: a sustainable pipeline for within-sample viral genetic diversity estimation
Source: Gigascience. 2024 Sep 30;13:giae065. doi: 10.1093/gigascience/giae065 (PMC11440432; doi:10.1093/gigascience/giae065)
Supplement: giae065_Reviewer_2_Report_Original_Submission — Shilpa Garg -- 3/13/2024 Reviewed [file giae065_reviewer_2_report_original_submission.pdf]

Reviewer Report

Title: V-pipe 3.0: a sustainable pipeline for within-sample viral genetic diversity estimation

Version: Original Submission **Date:** 3/13/2024

Reviewer name: Shilpa Garg

Reviewer Comments to Author:

V-pipe 3.0 is introduced as an advanced computational pipeline tailored for the analysis of next-generation sequencing data from short viral genomes. Designed to meet the challenges posed by the vast and diverse datasets generated by these technologies, V-pipe 3.0 emphasizes reproducibility, scalability, adaptability, and transparency. It achieves this by adhering to Snakemake's best practices, allowing easy swapping of virus-specific configuration files, and providing thoroughly tested examples online. The utility of V-pipe 3.0 is showcased through its application in two extensive data analysis projects, proving its efficacy in sustainable viral genomic data science.
Central to V-pipe 3.0 is its capacity for estimating viral diversity from sequencing data. A versatile benchmarking module has been developed to continuously assess various diversity estimation methods, accommodating the rapid advancements within this field. The pipeline simplifies the inclusion of new tools and datasets, supporting both synthetic and real experimental data. However, challenges in global haplotype reconstruction highlight the need for scalable methods that can accurately reflect the complex population structures of viruses and manage the uncertainties in the results.
Some additional clarification in the manuscript would be appreciated.
1) I'm curious about how the efficiency is attained.
2) Is it possible to utilize V-pipe for analyzing other microorganisms?
3) The authors might consider directing readers to the following review article for reference:
https://genomebiology.biomedcentral.com/articles/10.1186/s13059-021-02328-9
4) Identifying specific genes or genome regions with high polymorphism across different populations would be fascinating. How does V-pipe handle analysis in these highly variable regions?

Methods

Are the methods appropriate to the aims of the study, are they well described, and are necessary controls included? Choose an item.

Conclusions

Are the conclusions adequately supported by the data shown? Choose an item.

Reporting Standards

Does the manuscript adhere to the journal's guidelines on [minimum standards of reporting?](#) Choose an item.

Choose an item.

Statistics

Are you able to assess all statistics in the manuscript, including the appropriateness of statistical tests used? Choose an item.

Quality of Written English

Please indicate the quality of language in the manuscript: Choose an item.

Declaration of Competing Interests

Please complete a declaration of competing interests, considering the following questions:

- Have you in the past five years received reimbursements, fees, funding, or salary from an organisation that may in any way gain or lose financially from the publication of this manuscript, either now or in the future?
- Do you hold any stocks or shares in an organisation that may in any way gain or lose financially from the publication of this manuscript, either now or in the future?
- Do you hold or are you currently applying for any patents relating to the content of the manuscript?
- Have you received reimbursements, fees, funding, or salary from an organization that holds or has applied for patents relating to the content of the manuscript?
- Do you have any other financial competing interests?
- Do you have any non-financial competing interests in relation to this paper?

If you can answer no to all of the above, write 'I declare that I have no competing interests' below. If your reply is yes to any, please give details below.

I declare that I have no competing interests.

I agree to the open peer review policy of the journal. I understand that my name will be included on my report to the authors and, if the manuscript is accepted for publication, my named report including any attachments I upload will be posted on the website along with the authors' responses. I agree for my report to be made available under an Open Access Creative Commons CC-BY license (http://creativecommons.org/licenses/by/4.0/). I understand that any comments which I do not wish to be included in my named report can be included as confidential comments to the editors, which will not be published.

Choose an item.

To further support our reviewers, we have joined with Publons, where you can gain additional credit to further highlight your hard work (see: https://publons.com/journal/530/gigascience). On publication of this paper, your review will be automatically added to Publons, you can then choose whether or not to claim your Publons credit. I understand this statement.

Yes Choose an item.
