# Supplementary material for: V-pipe 3.0: a sustainable pipeline for within-sample viral genetic diversity estimation
Source: Gigascience. 2024 Sep 30;13:giae065. doi: 10.1093/gigascience/giae065 (PMC11440432; doi:10.1093/gigascience/giae065)
Supplement: giae065_Reviewer_3_Report_Original_Submission — Christophe Antoniewski -- 3/19/2024 Reviewed [file giae065_reviewer_3_report_original_submission.pdf]

Reviewer Report

Title: V-pipe 3.0: a sustainable pipeline for within-sample viral genetic diversity estimation

Version: Original Submission **Date:** 3/19/2024

Reviewer name: Christophe Antoniewski

Reviewer Comments to Author:

Review of Article: "V-pipe 3.0: A Sustainable Workflow for Diversity Estimation from Viral NGS Samples"
General Assessment
The article presents V-pipe 3.0, a Snakemake-based bioinformatics workflow designed for analyzing intra-sample variability in viral Next-Generation Sequencing (NGS) data. While the authors emphasize sustainability and scalability, several critical scientific concerns undermine the credibility and utility of the presented work.
Scientific Concerns
1. Poor Availability of Hypotheses, Results and Interpretations/Conclusions
The article lacks clarity regarding the actual results obtained from the implementation of V-pipe 3.0. While the authors mention the application of the workflow in large-scale projects ("Applications" 3.1, 3.2 and 3.3), specific outcomes, such as the accuracy of genetic evolution or diversity estimations or the identification of viral variants, are poorly presented.
Readers require concrete questions, findings and statistical analyses to assess the efficacy and reliability of the workflow. Without transparent reporting of results, the scientific value of the article diminishes significantly.
2. Ill-defined Hypotheses and Scientific Questions
The article fails to clearly articulate the underlying hypotheses or scientific questions addressed by V-pipe 3.0. While it outlines the technical aspects and functionalities of the workflow, the overarching scientific goals remain obscure.
Without well-defined research questions, it becomes challenging for readers to contextualize the significance of the presented work within the broader field of virology or computational biology.
3. Unverifiable Conclusions
The article concludes with a comparison of V-pipe 3.0 to other workflows, highlighting its purported advantages in terms of sustainability and functionality. However, the evaluation lacks empirical evidence or comparative analyses to substantiate these claims.
Readers are unable to independently verify the superiority of V-pipe 3.0 over alternative workflows, thereby undermining the credibility of the conclusions drawn by the authors.
There is a missing mark that is difficult to understand in this comparison, which casts a shadow over its relevance. Several Galaxy workflows were used, very early during the SARS-CoV-2 pandemic (first publication in August 2020 in PLoS Pathogens), to monitor the emergence of viral variants or assess intra-host variations. These workflows are still actively used and regularly updated (https://galaxyproject.org/projects/covid19/).
Technical Concerns

A limited number of target users
V-pipe can only be installed by researchers familiar with the use of a Bash script in a Linux terminal and the concepts of environments and dependencies. Once installed, the workflow must be configured to adapt it to specific needs, recognizing that most virologists have very different goals from national-level monitoring of intra- or inter-host variations. However, this configuration involves editing a yaml script and clearly requires relatively advanced programming skills given that V-pipe is complex software. Finally, even if a configuration file was built by a computer scientist, the correct execution of V-pipe, the access to the results as well as the verification that these results are computationally consistent can only be done by a person who is familiar with a Linux console and command lines to interact with. In the end, unfortunately, I do not believe that V-pipe will be useful to a large audience among virologists.
As it stands, V-pipe workflows are difficult to reproduce.
Given the objectives stated in this work, I obviously expected to be able to reproduce the analyses presented in section 3 (Applications), or to be able to run - with V-pipe - a pilot analysis used for comparison with other workflows. Unless I am mistaken, I have found, neither in the manuscript nor in the GitHub repository, any configuration file and the associated input data to reproduce, at least partially, even one figure of the article.
If we focus only on the V-pipe GitHub repository, we find the necessary documentation to install it easily (with the caveats mentioned above) but there is no obvious example to test the use of V-pipe. I'm lucky enough to be very familiar with GitHub Actions and so I looked for a testing workflow in these Actions that could serve as an example for a local test of V-pipe. I found the script "V-pipe/tests/regression_tests.sh", but its contents did not reassure me at all about the transparency of V-pipe workflows. Clearly missing from the GitHub repository is a concrete example of using V-pipe with real input data and a step-by-step explanation of configuring Snakemake and running a workflow.
Recommendations
1. Enhanced Results Reporting
- The authors should provide detailed summaries of empirical findings obtained through the application of V-pipe 3.0. This includes accuracy metrics, performance benchmarks, and comparative analyses with existing methods.
- Statistical measures, such as precision, recall, and computational efficiency, should be clearly presented to enable rigorous evaluation by the scientific community.
2. Clarification of Research Objectives
- Prioritize the elucidation of specific research questions or hypotheses addressed by V-pipe 3.0. Clearly articulate the scientific motivations behind the development and implementation of the workflow.
- By defining clear objectives, the authors can enhance the relevance and impact of their work within the field of viral genomics and bioinformatics.
3. Empirical Validation of Conclusions
- Substantiate claims regarding the superiority of V-pipe 3.0 through rigorous empirical validation. Conduct thorough comparative analyses with alternative workflows, incorporating diverse datasets and performance metrics.
- Transparently report the results of benchmarking studies and provide access to raw data and code repositories to facilitate reproducibility and independent verification.
Conclusion

While the article introduces an ambitious bioinformatics workflow for analyzing viral NGS data, several scientific deficiencies undermine its credibility and utility. By addressing concerns related to results reporting, hypothesis formulation, and conclusion verification, the authors can enhance the scientific rigor and impact of their work.
In its current state, implementation of V-pipe in research projects is lacking detailed instructions if a large audience of biologists is targeted. Ultimately the question is not so much whether V-pipe workflows are reproducible, scalable, adaptable and transparent, but rather for whom. In my opinion, additional work on the general architecture of the software and its documentation can still significantly improve these 4 aspects in order to reach a wider audience of virologists.

Methods

Are the methods appropriate to the aims of the study, are they well described, and are necessary controls included? Choose an item.

Conclusions

Are the conclusions adequately supported by the data shown? Choose an item.

Reporting Standards

Does the manuscript adhere to the journal's guidelines on minimum standards of reporting? Choose an item.

Choose an item.

Statistics

Are you able to assess all statistics in the manuscript, including the appropriateness of statistical tests used? Choose an item.

Quality of Written English

Please indicate the quality of language in the manuscript: Choose an item.

Declaration of Competing Interests

Please complete a declaration of competing interests, considering the following questions:

- Have you in the past five years received reimbursements, fees, funding, or salary from an organisation that may in any way gain or lose financially from the publication of this manuscript, either now or in the future?
- Do you hold any stocks or shares in an organisation that may in any way gain or lose financially from the publication of this manuscript, either now or in the future?

- Do you hold or are you currently applying for any patents relating to the content of the manuscript?
- Have you received reimbursements, fees, funding, or salary from an organization that holds or has applied for patents relating to the content of the manuscript?
- Do you have any other financial competing interests?
- Do you have any non-financial competing interests in relation to this paper?

If you can answer no to all of the above, write 'I declare that I have no competing interests' below. If your reply is yes to any, please give details below.

I declare that I have no competing interests

I agree to the open peer review policy of the journal. I understand that my name will be included on my report to the authors and, if the manuscript is accepted for publication, my named report including any attachments I upload will be posted on the website along with the authors' responses. I agree for my report to be made available under an Open Access Creative Commons CC-BY license (http://creativecommons.org/licenses/by/4.0/). I understand that any comments which I do not wish to be included in my named report can be included as confidential comments to the editors, which will not be published.

Choose an item.

To further support our reviewers, we have joined with Publons, where you can gain additional credit to further highlight your hard work (see: https://publons.com/journal/530/gigascience). On publication of this paper, your review will be automatically added to Publons, you can then choose whether or not to claim your Publons credit. I understand this statement.

Yes Choose an item.
